# Unspecific Peroxygenase‐Based Chemo‐Enzymatic Synthesis of Amino Alcohols for Pharmaceutical Preparation

**DOI:** 10.1002/cbic.70537

**Published:** 2026-09-20

**Authors:** Simon Last, Alina Gazizova, Katharina Wittmann, Niklas Dietz, Martin J. Weissenborn, Jan von Langermann

**Affiliations:** ^1^ Institute for Chemistry Biocatalysis Group Otto‐von‐Guericke‐University Magdeburg Germany; ^2^ Institute of Chemistry Organic Chemistry Group Martin‐Luther University Halle‐Wittenberg Halle (Saale) Germany

**Keywords:** amino alcohols, building blocks, chemo‐enzymatic conversion, pharmaceuticals, unspecific peroxygenase

## Abstract

This study explores the use of unspecific peroxygenases for the enantioselective preparation of amino alcohol‐based pharmaceuticals and their respective precursors. The aim of this work was to develop a practical concept by simplifying the otherwise complex synthesis approach, thereby expanding the application of unspecific peroxygenases toward chemo‐enzymatic synthesis. To introduce hydroxy groups at specific positions within protected amines, the unspecific peroxygenase (UPO) was used, which facilitated the enantioselective synthesis toward shorter overall reaction pathways and thus may serve as an addition to the preparative toolbox with this type of enzyme.

## Graphical abstract

The conversion of amines using nonspecific peroxygenases has always presented a challenge. The use of specific protecting groups, combined with the integration of enzymatic catalysis into conventional chemical methods, opens up new possibilities for the direct and enantioselective synthesis of valuable pharmaceuticals in an innovative, cost‐effective and environmentally friendly manner.

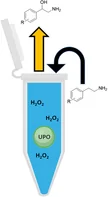

## 1 Introduction

The pharmaceutical industry remains one of the largest and most influential branches in the world across many countries and markets [1]. The number of active substances currently authorized and used to treat a broad range of diseases continues to rise worldwide. For this reason, the pharmaceutical industry is constantly on the lookout for new active ingredients and innovative synthetic routes for their production, often suffering from a lack of enantioselectivity. One promising approach involves combining biocatalytic methods with conventional chemical techniques to take advantage of the strengths of both procedures [2]. For example, enzymes enable specific groups to be incorporated into a molecule in an often highly enantioselective and straightforward manner [3]. Thus, unspecific peroxygenases (UPOs, EC 1.11.2.1) are capable of oxyfunctionalizing an otherwise unreactive C─H bond using only hydrogen peroxide as a cosubstrate, producing water as the sole by‐product, while giving high enantiomeric excesses (ee) [4, 5, 6, 7, 8, 9, 10, 11, 12, 13]. These originally fungal heme‐thiolate enzymes are able to convert a wide variety of substrates but normally suffer from their inability to convert primary amines toward valuable amino alcohols [4, 14, 15]. This is due to complexation of the iron ion contained in the active site of the enzyme with free amines and subsequent inactivation of the enzyme due to the rivaling binding to the nitrogen, which renders any further conversion via oxyfunctionalization impossible [14]. The introduction of a suitable protecting group to the respective amine can solve this problem, opening up new reaction paths, leaving only the control of the provided amount of H_2_O_2_ as the remaining challenge, which was already addressed in a previous study [16].

**FIGURE 1:**
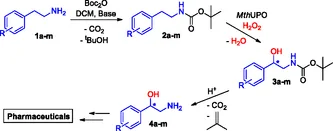
General reaction scheme for the chemo‐enzymatic synthesis of pharmaceuticals.

In this work, the *tert*‐butyloxycarbonyl protecting group (Boc group) was used for this purpose (Figure 1) [17]. The Boc group allows for the selective chemical reaction using aggressive agents, which would otherwise also attack at the amino position and is commonly used in organic synthesis [18, 19]. The Boc group supports the conversion of normally inaccessible amine‐containing substrates through the UPO by removing electron density from the amine [20]. Following successful enzymatic conversion, the protecting group can be easily removed in the presence of an acid to yield amino alcohols. These compounds are either already pharmaceutically active by themselves or can be considered as precursor compounds for further syntheses.

Approximately 40% of all pharma‐ and agrochemicals contain at least one structural equivalent of amines of any kind [21]. In particular, amino alcohols, especially if they are enantiomerically pure, are of great interest to the pharmaceutical industry. Consequently, any new method for synthesizing such compounds can be regarded as a highly valuable addition to the preparative toolbox.

Amino alcohols are typically obtained by the reduction of toxic cyanohydrins, azido‐, or nitroaldol compounds [22, 23, 24]. However, they generally require expensive transition‐metal catalysts (Pd, Ru, etc.), involving a certain cost in industry and adding preparatory difficulties in order to avoid catalyst deactivation [25, 28]. Cyanohydrins can also be synthesized and converted into amino alcohols with the aid of enzymes [29, 30, 31]. The same applies to azidoaldols, also showing good conversion values and high enantioselectivity [32]. Another alternative synthetic route involves the ring opening of epoxides with ammonia or amines, which also gives good yields [2, 33]. It is also possible to perform the ring‐opening reaction using certain enzymes, allowing for the usage of different nucleophiles and yielding various substituted alcohols [34, 35].

Using the right enzymes not only makes it possible to avoid hazardous chemicals for the sake of cost‐effectiveness and environmental protection but it can also simplify otherwise complicated reaction processes. One option is the use of transaminases (EC 2.6.1.‐), transferring amino groups from a donor molecule to an acceptor molecule, thereby achieving high selectivity [36, 37]. However, transaminases often suffer from an unfavorable chemical reaction equilibrium, requiring additional adjustments to the reaction system [38, 39]. By comparison, the use of a UPO requires only the presence of H_2_O_2_ and produces water as its sole by‐product [40]; it should therefore be the preferred option, as it represents the more environmentally friendly alternative, also offering a favorable chemical equilibrium, high selectivity, and good ee.

The combination of enzymes for the targeted modification of molecules, followed by conventional chemical methods, is designed to reduce the overall number of reaction steps and can, consequently, also help to cut production costs for sought‐after components. Aromatic ethylamine derivatives were used as model compounds in this study; these were first protected with a Boc group, converted into the respective amino alcohols by the UPO, deprotected, and finally processed into various pharmaceutical compounds.

## 2 Results and Discussion

### 2.1 Substrate Screening

As ethylamine derivatives are widely found in a wide range of pharmaceuticals, compounds with this structural feature were selected as substrates for this study and tested for their compatibility with unspecific peroxygenases (compare Figure 1). The main focus was on finding a compound which, on the one hand, would be unable to form a durable complex with the iron present in the enzyme’s active site—which would lead to the deactivation of UPO—and, on the other hand, exhibit the highest possible activity in order to achieve high conversion values and enantioselectivity. Similar studies have already been carried out by Püllmann et al., who reacted phthalimide‐protected amines with *Mth*UPO [4]. However, both the yield and the enantioselectivity appeared to leave room for improvement. For this purpose, two unspecific peroxygenases were tested—*Mth*UPO from *Myceliophthora thermophila* and *Tte*UPO from *Thielavia terrestris*.

The investigated substrates were selected, as described previously, based on their chemical structure, which is also found in many potential pharmaceuticals. Table 1 shows both the structures of the compounds investigated and the conversion observed for the respective enzymes after a specific period of time. The compounds were each equipped with a Boc group at the amine position before being added to the enzyme‐containing reaction solution. Concentrations of 10 mM was used in each case, and the samples were incubated for 6 h at 30 °C and 800 rpm in a shaking incubator. For solubility reasons, the reaction medium contained 10 vol% of acetonitrile. Each reaction mixture contained 350 µL of a concentrated enzyme solution (1.44 U/mL). To avoid deactivation of the UPO, the required hydrogen peroxide was not added directly to the reaction solution but was generated in situ using an L‐amino acid oxidase (*hc*LAAO4, EC 1.4.3.2) from *Hebeloma cylindrosporum* [16, 41]. L‐alanine was chosen as substrate for this second enzyme, which can convert an L‐amino acid into the corresponding α‐keto acid while producing hydrogen peroxide for the UPO. The test reactions were stopped after a duration of 6 h in order to compare the conversion values of the respective substrates. Over a period of 24 h, complete conversions were achieved, providing oxyfunctionalized intermediates for further reactions.

**TABLE 1:** Overview of the substrates screened for *Mth*UPO and *Tte*UPO, with conversions shown as a percentage. Values for ee given beneath respective conversion. 1 mL scale, 10 mM per substrate, 350 µL of respective UPO (1.44 U/mL), 6 h, 30 °C, 800 rpm. Each compound was equipped with a Boc protecting group on the amine position.

#	Substrate	*Mth*UPO	*Tte*UPO
a	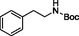	24% 82%(*S*)	8% n.d.
b	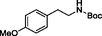	22% n.d.	3% n.d.
c	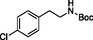	21% n.d.	9% n.d.
d	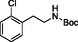	—	—
e	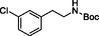	43% >99.9%(*S*)	4% n.d.
f	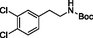	31% 99.6%(*S*)	12% n.d.
g	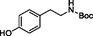	—	6% n.d.
h	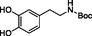	—	—
i	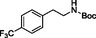	—	6% n.d.
j	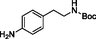	—	12% n.d.
k	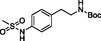	—	—
l	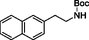	—	—
m	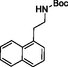	—	—

It became apparent that although *Tte*UPO has a significantly broader substrate range, it is only capable of generating quite low turnover compared to *Mth*UPO for the Boc‐protected substrates. For this reason, *Mth*UPO was used in all subsequent experiments. From the substrates for which a reaction was observed, the three best were selected for further investigation. These are *tert‍*‐‍butyl (phenethyl)carbamate (**2a**), *tert*‍‐‍butyl (3‐chlorophenethyl)‐carbamate (**2e**), as well as *tert*‐butyl (3,4‐dichlorophenethyl)‐carbamate (**2f**).

One reason for the altered reaction behavior and the resulting compatibility of the chlorinated substrates with the enzyme can be attributed to the inductive effect of the chlorine atoms, which influences the other substituents on an aromatic compound. The electron‐withdrawing effect in the meta‐ or para‐position can facilitate the cleavage of a C─H bond in the benzylic position. Only the analog **2d**, chlorinated in the ortho‐position, exhibits no activity whatsoever, which is due to the proximity of the chlorine to the benzylic position in the molecule and the resulting steric hindrance.

In the case of *tert*‐butyl (phenethyl)carbamate (**2a**), the increased reactivity compared to the free amine is due solely to the presence of the Boc protecting group. In this example, this behavior is the result of the mesomeric effect exerted by the carbonyl group contained in the electron‐withdrawing protecting group. This removes electron density from the amine, with the result that‍—‍together with steric hindrance caused by the protecting group—the amine is no longer able to form a durable complex with the iron present in the enzyme’s active site. An enzymatic reaction becomes possible without the heme center being blocked, and the product can leave the active site after the oxyfunctionalization. The same applies to the compounds **2e** and **2f**, which additionally benefit from the electron‐withdrawing effects of the chlorine substituents on the aromatic ring.

Some of the other substrates shown also contain substituents that exert a negative inductive effect—such as amino or hydroxy groups. Nevertheless, these could not be converted by *Mth*UPO and could only be converted to a limited extent by *Tte*UPO. This pattern can be explained either by the active site’s susceptibility to these groups or by the varying strength of the inductive effect of these groups due to their differing electronegativity. Thus, substrates that possess exposed groups with high electronegativity and reactive lone pairs on the aromatic ring, which can coordinate with the enzyme’s metal center, will always lead to the inhibition of UPO. This is also consistent with the findings of previous studies [4]. Although *Tte*UPO exhibits activity toward *tert*‐butyl (4‐aminophenethyl)carbamate (**2j**), which is attributable to the specific modification of this concrete enzyme, this activity is not high enough to be of relevance to this study. Similarly, the lack of activity for the remaining substrates can be traced back to the structure of the enzymes’ active site. This could be addressed through further modifications like additional introductions of protection groups to shield interfering groups but is not the aim or focus of this work.

With regard to the synthesis of amino alcohols, it could be noted that the introduction and removal of a protecting group are steps that could also be avoided using other methods. Whilst this is certainly true, it should be pointed out that other methods, such as the chemical reduction of suitable precursor compounds, generally yield significantly lower ee. However, compared with conventional chemical synthesis methods for amino alcohols, it is worth noting that this approach makes it possible to avoid the use of any harmful or otherwise hazardous chemicals. The only by‐product generated during the production of the protected amino alcohols (**3**) is water. Nevertheless, this cannot be said of the introduction of the Boc protecting group. Although the same number of atoms are introduced prior to the enzymatic reaction as are cleaved off afterward, CO_2_ and *tert*‐butanol are lost during the introduction when the Boc_2_O used is cleaved. As a result, the overall atom efficiency has to be seen as significantly lower if the protection reaction is taken into account.

### 2.2 Subsequent Reactions to Chiral Pharmaceuticals

After conversion of the Boc‐protected substrates with *Mth*UPO, the products (**3**) were isolated. To cleave the protecting group, the compounds were exposed to an acidic pH in order to obtain the free amino alcohols (**4**, compare Figure 1). Based on these compounds, further syntheses were carried out. The enzymatic reactions were performed, as explained in the Supporting Information.

#### 2.2.1 (*S*)‐2‐Amino‐1‐Phenylethanol (**4a**) and the Pathway to (*S*)‐2‐(1*H*‐imidazol‐1‐yl)‐1‐Phenylethanol (**5a**)

**FIGURE 2:**
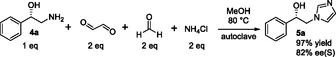
Reaction scheme for the synthesis of 2‐(1*H*‐imidazol‐1‍‐‍yl)‐1‐phenylethanol (**5a**).

The reaction path chosen for this work is displayed in Figure 2.

Following the deprotection reaction, **4a** was obtained in a quantitative fashion. This compound was synthesized with an enantiomeric excess of 82% for the (*S*)‐enantiomer. 2‐Amino‐1‐phenylethanol represents a very valuable amine alcohol precursor as its structure can be found in a wide variety of other pharmaceuticals. **4a** can also be obtained following different routes, for example, by opening the epoxide ring of styrene oxide [2, 42], but the straight‐forward enzymatic preparation shown in this work appears to be more practical. As already mentioned, various other pharmaceutical compounds can be derived from **4a** [31, 43]. Furthermore, this compound is also used in its own right, for example, as a neuraminidase inhibitor to combat influenza viruses [44] or as a medication for the treatment of Alzheimer’s disease [45].

2‐(1*H*‐imidazol‐1‐yl)‐1‐phenylethanol (**5a**) was selected as a suitable product for further synthesis. This compound is a pharmaceutical agent that has a wide range of applications, for example, as an insecticide [46], as an antifungal [47, 48], and even as an anticancer agent [49]. Compound **5a** can be obtained in various ways—normally by reaction of phenacyl bromide with imidazole followed by reduction using hydrogen and transition metal complex catalysts or using a halogenated alcohol precursor in which the halogen leaving group can be exchanged for imidazole [50]. Another suitable reaction path would be using an epoxide and imidazole in order to fulfill a ring‐opening reaction, resulting in good yields of around 76% [51].

Although these methods are certainly very effective, the same product can also be obtained by a simpler route, in which the enantiomeric distribution depends solely on the orientation of the amino alcohol used. The reaction path chosen for this work is displayed in Figure 2. It utilizes high pressure in order to force the formation of an imidazole ring system at the position of the amino group. This is a condensation reaction in which water is produced as a nontoxic by‐product, and no additional catalyst is required. A sealed, pressure‐resistant reaction vessel had to be used as the formaldehyde employed is volatile and would have evaporated from the reaction mixture at elevated temperatures. Two equivalents each of glyoxal, formaldehyde, and ammonium chloride are added in order to shift the reaction equilibrium further toward the products, in accordance with Le Chatelier’s principle. This way, the reaction´s yield was 97%, which significantly exceeds the yields of alternative reactions [47, 51] and is in line with the expected value for this reaction [46].

The major advantage of this reaction is, on the one hand, that it does not require expensive and, in some cases, toxic transition‐metal catalysts as other procedures do [27]. Furthermore, neither the enzymatic oxyfunctionalization nor the subsequent generation of the imidazole group produces any environmentally harmful by‐products. Consequently, compound **5a** can be produced in an innovative, cost‐effective, and efficient manner, covering a wide range of applications. **5a** can also serve as a precursor for other pharmaceuticals [28, 52]. As for the reaction pathway involving a ketone [53, 54, 55], which must be reduced after synthesis to yield the alcohol—whilst this route is certainly feasible, depending on the method used, it requires various additional steps as well as a large number of environmentally harmful chemicals for the extraction and processing of the products. The chemo‐enzymatic variant described in this paper, which does not require an intermediate ketone step, not only saves resources and reduces the number of steps involved but also achieves a yield that is at least as high and is therefore preferable to conventional methods.

#### 2.2.2 (*S*)‐2‐Amino‐1‐(3‐Chlorophenyl)ethanol (**4e**) and the Pathway to (*S*)‐2‐((2‐(Benzyloxy)benzyl)amino)‐1‐(3‍‐‍Chlorophenyl)‐Ethanol (**5e**)

**FIGURE 3:**
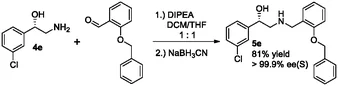
Reaction scheme for the synthesis of 2‐((2‐(benzyloxy)benzyl)amino)‐1‐(3‐chlorophenyl)ethanol (**5e**).

The chosen reaction pathway for the synthesis of compound **5e** is shown in Figure 3.

As with the unsubstituted derivative, compound **4e** was also obtained following deprotection. It was synthesized with an enantiomeric excess of >99.9% for the (*S*)‐enantiomer, where the (R)‐enantiomer could not even be detected using LC–MS. **4e** is used as an active agent that targets β‐adrenergic receptors [56, 57]. Compound **4e** can also be prepared chemically. One way to do this is to reduce the corresponding cyanohydrin with lithium aluminum hydride, whereby the cyano group is converted into an amino group. The required cyanohydrin can be obtained beforehand by reacting the ketone with hydrocyanic acid; however, this is an efficient but dangerous reaction. The reduction of the corresponding azide is also possible but suffers from the same problems [23, 32]. In contrast, the UPO method requires no toxic substrates whatsoever, so the small additional step involved in preparing the protected amine seems entirely justifiable. Alternatively, the reduction of the corresponding amino ketone to the amino alcohol can also be carried out using transition‐metal catalysts [26, 58]; whilst this is highly efficient, it involves greater preparative effort and requires the use of expensive precious metal compounds. In comparison, the enzyme is inexpensive and is readily available.

The product selected for a follow‐up reaction was 2‐((2‐(benzyloxy)benzyl)amino)‐1‐(3‐chlorophenyl)ethanol (**5e**), a potent antibiotic [59, 60]. In this case, the enzymatic oxyfunctionalization to introduce a hydroxy group in the benzylic position prior to the chemical step represents an innovation compared to the conventional approach, which utilizes 3‐chlorobenzaldehyde as well as toxic trimethylsilyl cyanide as starting materials [59]. Nevertheless, the reaction of the resulting amino alcohol (**4e**) proceeded in a very straightforward manner without the need for harmful chemicals, as shown in Figure 3.

This is a reductive alkylation reaction at the amino group, in which 2‐(benzyloxy)benzaldehyde was used to first form an imine, which was then reduced under basic conditions with sodium cyanoborohydride to the corresponding secondary amine. The reaction was performed at room temperature and gave an 81% yield. The use of a mild reducing agent was crucial to the success of the synthesis as overly aggressive reagents could lead to unwanted side reactions. Sodium cyanoborohydride selectively reduces imines, ketones, and aldehydes and can be used in a wide range of solvents, such as water, methanol, or dimethyl sulfoxide. **5e** is just one example of a variety of active ingredients that can be produced in a similar way [59]. By substituting functional groups, it is possible to achieve varying degrees of potency among the different analogs.

#### 2.2.3 (*S*)‐2‐Amino‐1‐(3,4‐Dichlorophenyl)ethanol (**4f**) and the Pathway to (S)‐1‐(3,4‐Dichlorophenyl)‐2‐((Naphthalen‐2‍‐‍ylmethyl)ami‐no)ethanol (**5f**)

**FIGURE 4:**
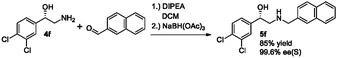
Reaction scheme for the synthesis of 1‐(3,4‍‐‍dichlorophenyl)‐2‐((naphthalen‐2‐ylmethyl)amino)ethanol (**5f**).

Figure 4 displays the chosen reaction pathway for the synthesis of compound **5f**.

As with the previous compound, **3f** was also first deprotected following enzymatic oxyfunctionalization to give the amino alcohol 2‐amino‐1‐(3,4‐dichlorophenyl)ethanol (**4f**). This compound itself already acts as an active ingredient. For example, the (*S*)‐enantiomer blocks octopamine receptors in American cockroaches (*Periplaneta americana*) [57], whilst the (*R*)‐enantiomer can be used as a beta‐blocker in mammals [61]. As a racemate, **4f** can be applied as a medication for leishmaniasis [62]. Compound **4f** can also be obtained by reducing the corresponding ketone [58]. Here too, it can be said that conventional chemical synthesis is certainly feasible and delivers high yields, but it always involves the use of expensive, toxic, and environmentally harmful chemicals. However, the enzymatic conversion yielded a 99.6% enantiomeric excess of the (*S*)‐enantiomer, offering a viable alternative and interesting possibilities for pharmaceutical applications.

Compound **4f** also serves as a precursor for other pharmaceuticals. For this study, 1‐(3,4‐dichlorophenyl)‐2‐((naphthalen‐2‐ylmethyl)amino)ethanol (**5f**) was selected as the end product, being a medicine used to treat malaria [63]. There are also other analogs with similar effects. For the synthesis, **4f** was dissolved in dichloromethane and reacted with 2‐naphthaldehyde under basic conditions in a stirred flask, initially forming an imine, which was then converted into a secondary amine using sodium triacetoxyborohydride, as shown in Figure 4. The reaction gave a yield of 85%, which is in line with literature.

The reduction of the imine was deliberately carried out using a relatively mild reducing agent. Sodium triacetoxyborohydride selectively reduces aldehydes and imines but would leave ketones unaffected. This is a synthesis typically employed for this compound. The difference lies in the preparation of the amino alcohol (**4f**), which, as in the cases mentioned above, could have been produced chemically but in this instance was generated enzymatically. As in the cases discussed previously, the use of harmful chemicals is largely avoided here as well. Furthermore, it can be argued that although the detour via the introduction of the Boc protecting group for the enzymatic conversion of the amine to the amino alcohol does not save any reaction steps in this example, it is nevertheless justifiable.

## 3 Conclusion

This study has demonstrated that valuable enantiopure amino alcohol‐based pharmaceuticals and their respective precursors are accessible via unspecific peroxygenases. The key intermediary step is the enantioselective oxyfunctionalization of the Boc‐protected amine, which otherwise would undergo a direct inhibition of the active site of the UPO.

An important aspect of this work was the attempt to develop alternative synthetic routes by combining biocatalysis with conventional chemistry, which ultimately either reduces the number of reaction steps or offers other advantages. It may therefore seem counterintuitive to introduce additional reaction steps by using the Boc protecting group. However, enzymatic oxyfunctionalization, which then becomes possible, in turn saves at least two reaction steps—namely, the oxidation to the ketone and the subsequent conversion to the alcohol—on the path to obtain the desired products. This also occurs in an enantioselective manner, which offers obvious benefits for later pharmaceutical usage and preferentially in the benzylic position, which is generally considered to be unreactive due to a lack of activation. However, the UPO enables the selective cleavage of the C─H bond to catalyze the formation of a hydroxyl group.

**FIGURE 5:**
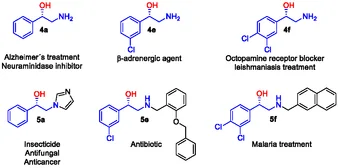
Compilation of the pharmaceuticals presented in this work.

A variety of substrates were successfully converted with satisfactory yields. The amino alcohols obtained are themselves pharmaceutical compounds and do not require further processing. However, a suitable subsequent product was identified and synthesized for each intermediate compound (**4a**, **4e**, and **4f**). These follow‐up products (**5a**, **5e**, and **5f**) comprise a selection of various active ingredients that can be used, amongst other things, as antibiotics, insecticides, and fungicides, or in the treatment of cancer and malaria, as shown in Figure 5. The versatility of combining enzymatic and conventional chemical methods has thus been conclusively demonstrated, particularly as many other analogs are conceivable in addition to the compounds shown.

A major advantage of enzymatic catalysis lies in the high enantioselectivity, which is also the case here. Furthermore, it could be observed that the presence of substituents on the aromatic ring contained in the substrate can lead to a further increase in enantiomeric excess. With regard to applications in the pharmaceutical industry, the ability to produce enantiomerically pure active substances is a major advantage. The method described here makes this possible as compounds **3e** and **3f** were formed with ee(*S*) > 99%.

It also became apparent that, depending on the substitution of the aromatic ring, the catalysis of the oxyfunctionalization reaction can become impossible even with a Boc protecting group attached. Thus, substituents with reactive free electron pairs continue to lead to deactivation of the enzyme. Such substrates still could not be converted.

In summary, it can be said that the objective of this work has been achieved and that complementary methods to existing synthetic processes for the production of amino alcohols in general and valuable pharmaceuticals in particular have been demonstrated. Another aspect that should not be overlooked is the potential to avoid the use of environmentally harmful chemicals. Equally important are the potential financial savings resulting from a reduction in the number of reaction steps required.

## Funding

This study was supported by Deutsche Forschungsgemeinschaft (505185500 and 450014604) and European Regional Development Fund (ZS‐2023‐12‐182329).

## Conflicts of Interest

The authors declare no conflicts of interest.

## Supporting information

Supplementary Material

## Data Availability

The data that support the findings of this study are available in the supplementary material of this article.
