# Supplementary material for: Unspecific Peroxygenase‐Based Chemo‐Enzymatic Synthesis of Amino Alcohols for Pharmaceutical Preparation
Source: Chembiochem. 2026 Sep 20;27(18):e70537. doi: 10.1002/cbic.70537 (PMC13591206; doi:10.1002/cbic.70537)
Supplement: Supplementary file 1 [file CBIC-27-e70537-s001.pdf]

Supporting Information

Unspecific peroxygenase-based chemo-enzymatic synthesis of amino alcohols for pharmaceutical preparation

Simon Last,[a] Alina Gazizova,[a] Katharina Wittmann,[a] Niklas Dietz,[b] Prof. Dr. Martin J. Weissenborn,[b] and Jan von Langermann*[a]

[a] Otto-von-Guericke University, Institute for Chemistry, Biocatalysis Group, Magdeburg Germany
corresponding author: Tel. +49-(0)3916751440, jan.langermann@ovgu.de

[b] Martin-Luther University Halle-Wittenberg, Institute of Chemistry, Organic Chemistry Group, Halle (Saale) Germany

Table of Contents

1. General Information ... 2

2. NMR spectra ... 2

3. Preparation of biocatalyst ... 24

4. Enzymatic conversion ... 25

5. Performed synthesis ... 25

6. Photometric measurements ... 27

7. Gas chromatography ... 28

8. HPLC analytics and determination of enantiomeric excess ... 30

1. GENERAL INFORMATION

^{1}H and ^{13}C spectra were measured on an AV III Bruker-BioSpin (^{1}H: 400.13 MHz; ^{13}C: 100.62 MHz) or an Avance Neo Bruker BioSpin (^{1}H: 600.13 MHz; ^{13}C: 150.9 MHz). The conversion of all substrates was observed using chromatographic methods. All chemicals and solvents used for synthesis, cell production, processing and analytics were purchased from commercial distributors. These are Carl Roth (Karlsruhe, Germany), Merck (Darmstadt, Germany), abcr (Karlsruhe, Germany) and Thermo Fisher Scientific (Waltham, MA, USA). Furthermore, SC Drop-Out (-Uracil) for the cultivation of yeast was purchased from Formedium LTD (Kings Lyn, England). Additional analytical samples were purchased from Enamine Germany (Frankfurt am Main, Germany). Solvents for HPLC/MS were purchased in LC/MS grade from Fisher Scientific.

2. NMR SPECTRA

The NMR data for the compounds that were ultimately processed into pharmaceuticals and discussed in the main body of the text are presented below. Additionally, there are NMR spectra and data of compounds, which were mentioned in the main text, but not further processed. For some NMR spectra, which were measured in $CDCl_3$, there can be seen a signal of the calibration agent TMS at 0 ppm.

tert-butyl phenylethylcarbamate (**2a**)

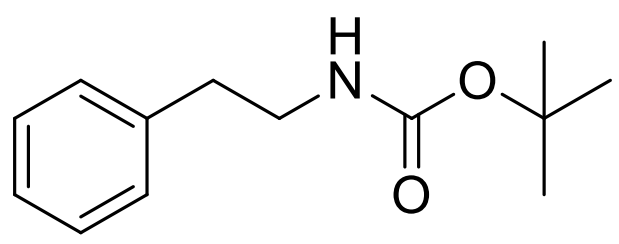

^{1}H NMR ($CDCl_3$, 400 MHz) δ [ppm]: 7.32 (m, 1H, Ar-H_{para}), 7.31 (s, 1H, $CDCl_3$), 7.29 (m, 2H, Ar-H_{meta}), 7.20 (m, 2H, Ar-H_{ortho}), 3.37 (t, J = 6.36 Hz, 2H, N-CH_2-C), 2.80 (t, J = 6.85 Hz, 2H, Ar-CH_2), 1.44 (s, 9H, C-CH_3).

^{13}C HMR ($CDCl_3$, 100 MHz) δ [ppm]: 155.8 (s), 138.9 (s), 128.7 (s), 128.5 (s), 126.3 (s), 79.1 (s) 77.0 (t, J = 31.54 Hz, $CDCl_3$), 41.7 (s), 36.1 (s), 28.3 (s).

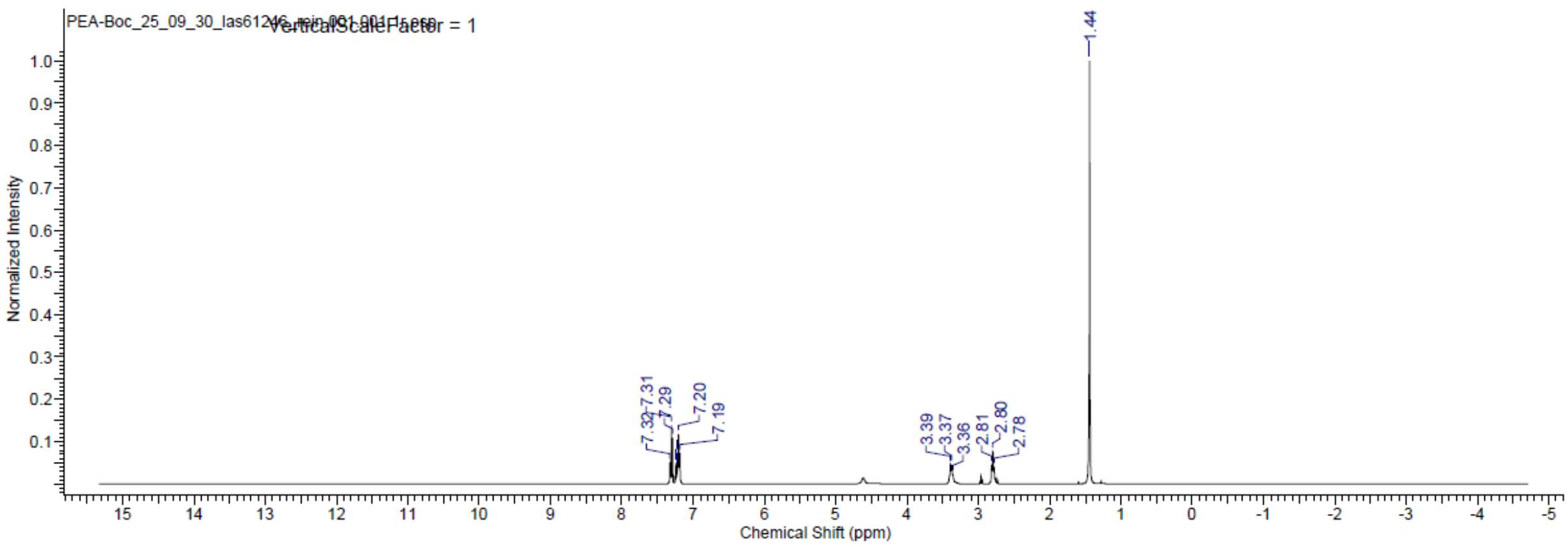

Figure S1. ^{1}H-NMR spectrum of **2a**.

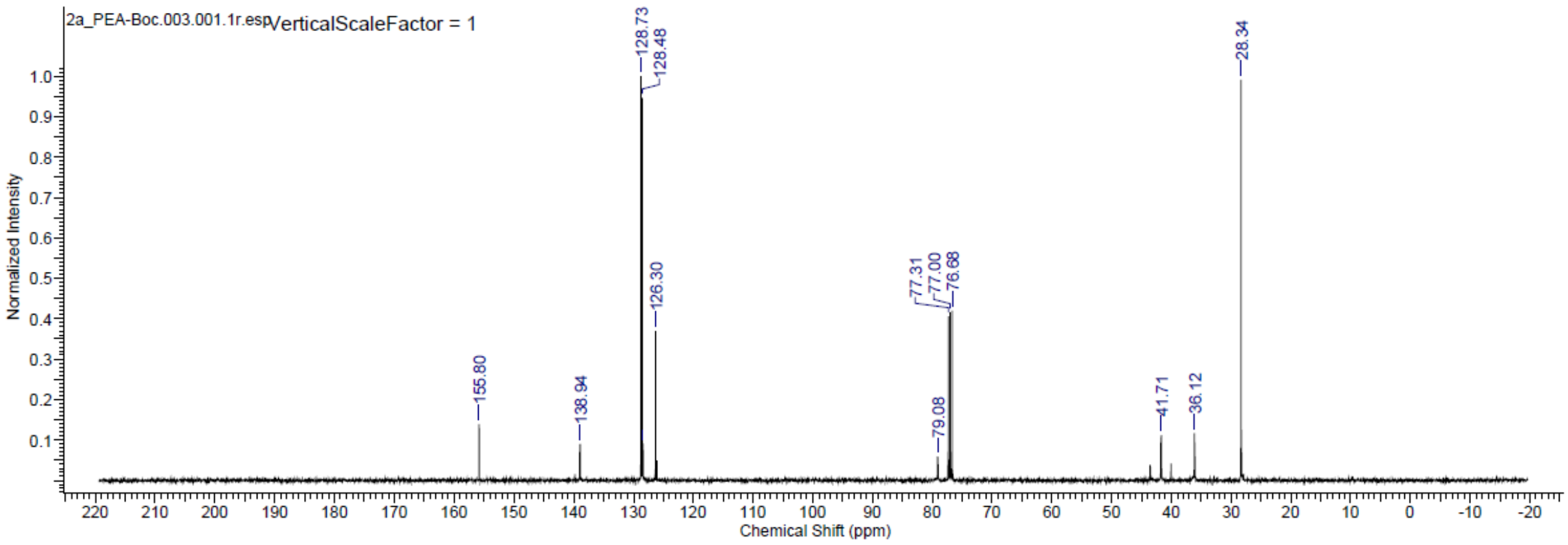

Figure S2. ^{13}C-NMR spectrum of **2a**.

<u>*tert*-butyl 4-methoxyphenethylcarbamate (**2b**)</u>

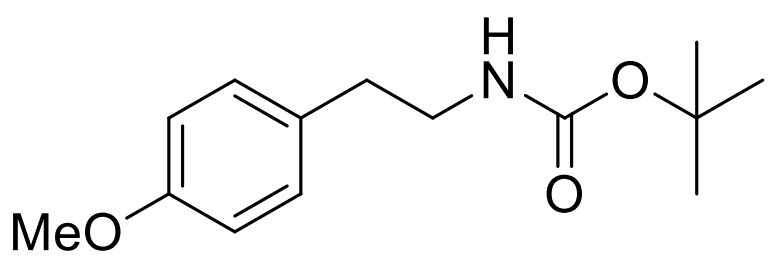

^{1}H NMR ($CDCl_3$, 600 MHz) δ [ppm]: 7.10 (d, J = 8.36 Hz, 2H, CH_{ortho}), 6.84 (d, J = 8.72 Hz, 2H, CH_{meta}), 3.78 (s, 3H, O-CH_3), 3.33 (dd, J = 6.18 Hz, 2H, N-CH_2), 2.73 (t, J = 6.90 Hz, 2H, Ar-CH_2-C), 1.43 (s, 9H, C-CH_3).

^{13}C HMR ($CDCl_3$, 150 MHz) δ [ppm]: 158.1 (s), 155.8 (s), 130.9 (s), 129.6 (s), 113.9 (s), 79.0 (s), 77.0 (t, J = 32.70 Hz, $CDCl_3$), 55.1 (s), 41.9 (s), 35.2 (s), 28.3 (s).

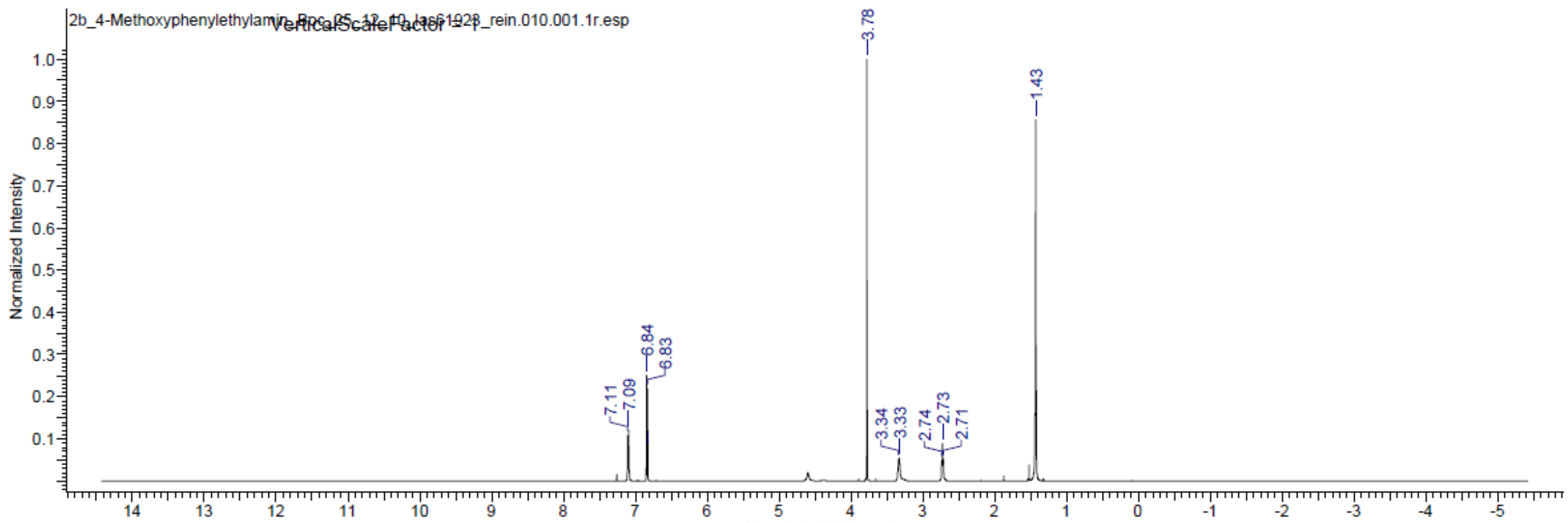

Figure S3. ^{1}H-NMR spectrum of **2b**.

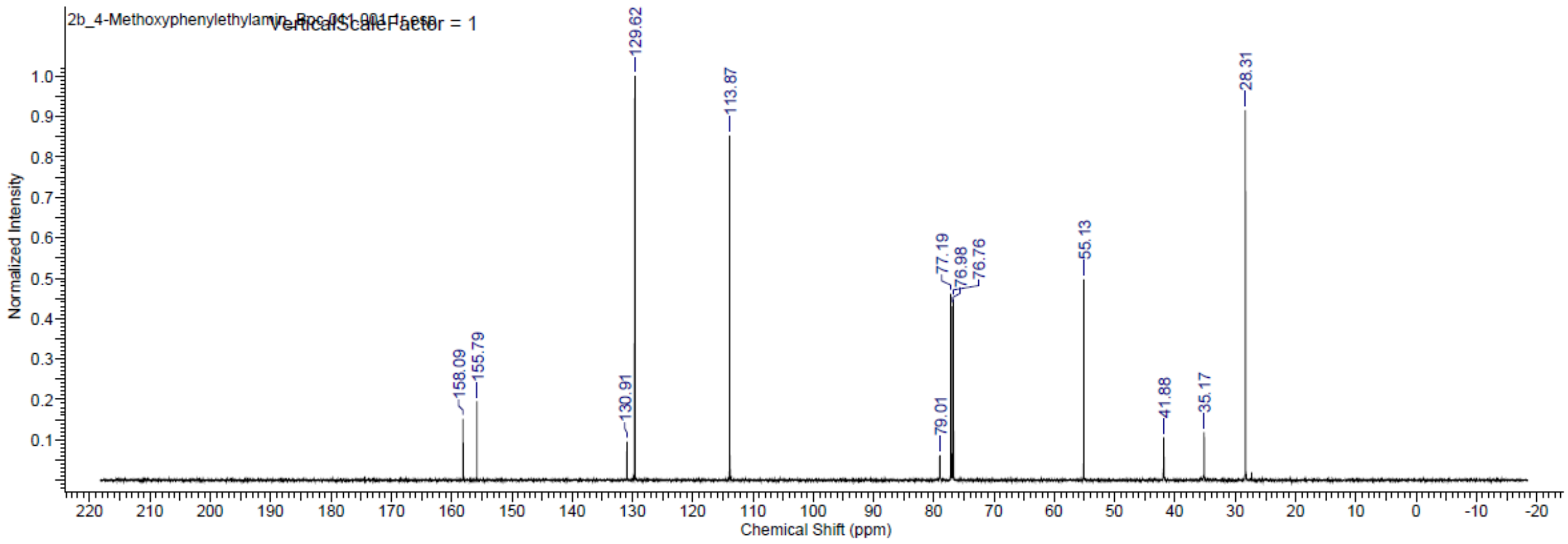

Figure S4. ^{13}C-NMR spectrum of **2b**.

<u>*tert*-butyl 4-chlorophenethylcarbamate (**2c**)</u>

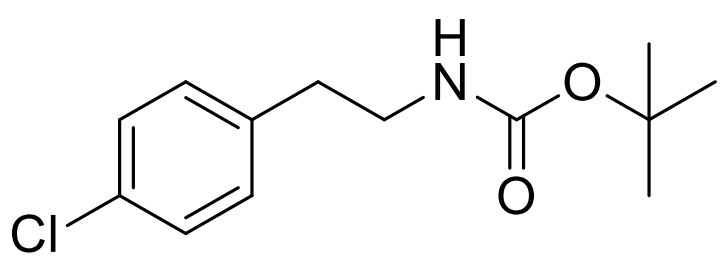

^{1}H NMR ($CDCl_3$, 600 MHz) δ [ppm]: 7.25 (d, *J* = 8.54 Hz, 2H, CH_{meta}), 7.11 (d, *J* = 8.17 Hz, 2H, CH_{ortho}), 3.33 (dd, *J* = 6.36 Hz, 2H, N-CH_2), 2.75 (t, *J* = 6.90 Hz, 2H, Ar-CH_2-C), 1.42 (s, 9H, C-CH_3).

^{13}C HMR ($CDCl_3$, 150 MHz) δ [ppm]: 155.8 (s), 137.4 (s), 132.1 (s), 130.1 (s), 128.6 (s), 79.2 (s) 77.0 (t, *J* = 31.61 Hz, $CDCl_3$), 41.6 (s) 35.5 (s), 28.3 (s).

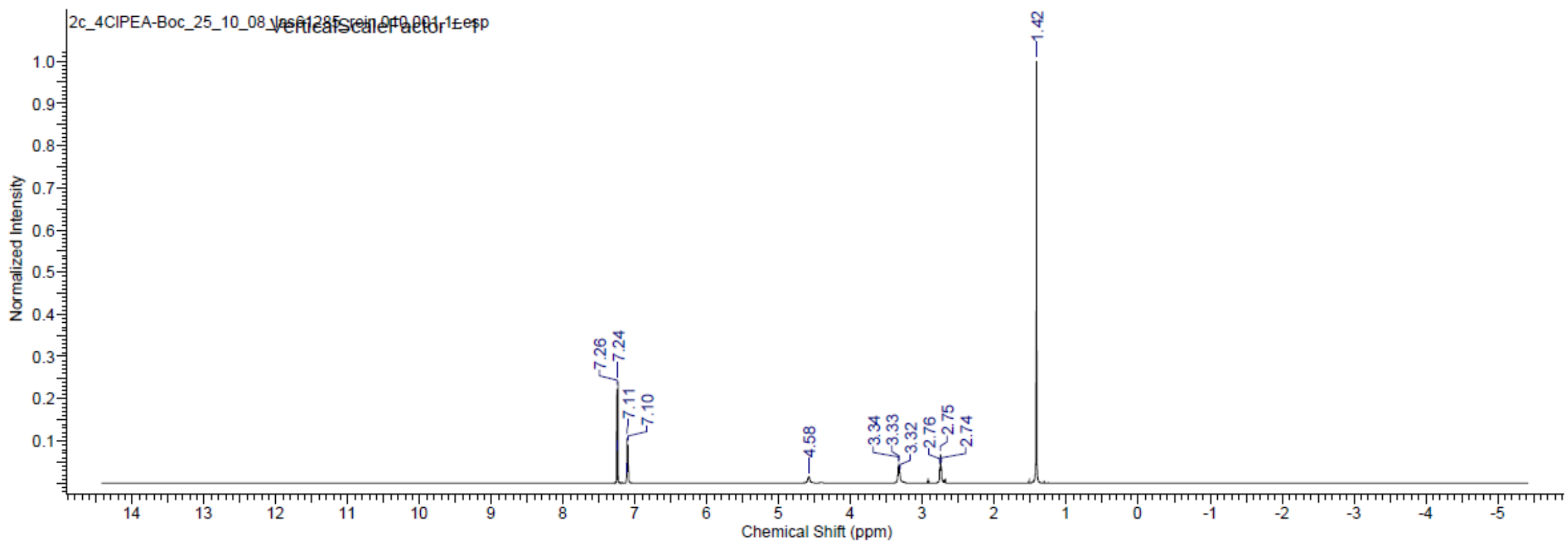

Figure S5. ^{1}H-NMR spectrum of **2c**.

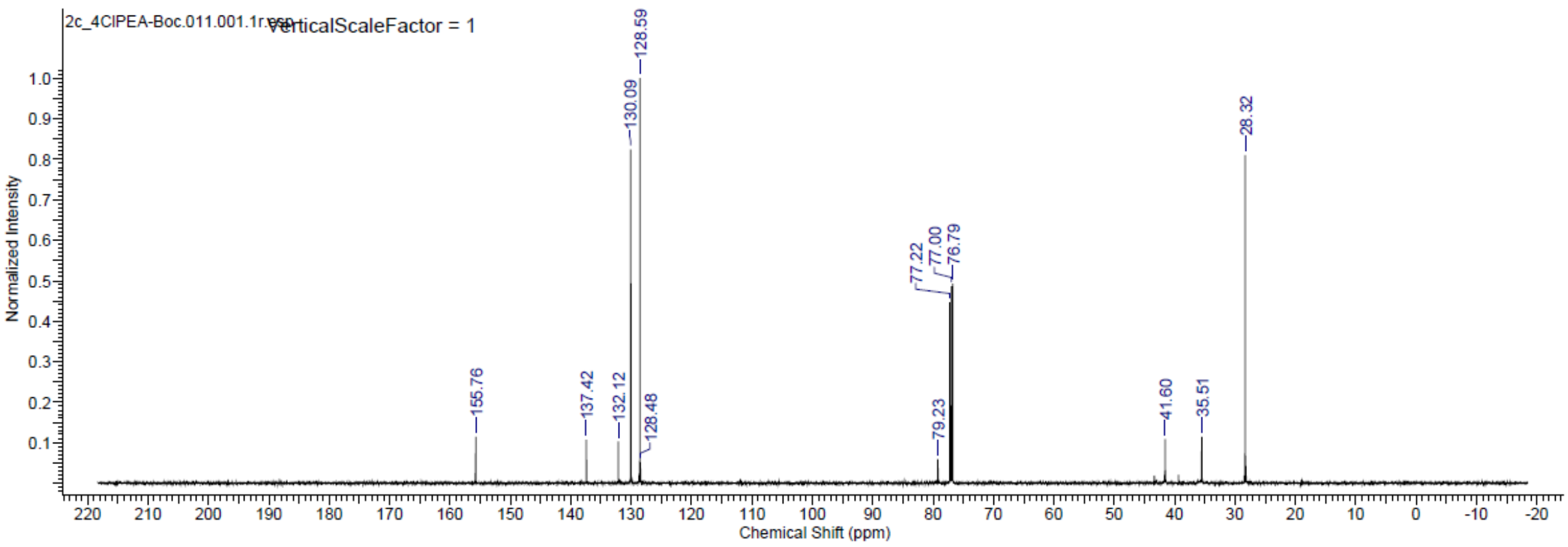

Figure S6. ^{13}C-NMR spectrum of **2c**.

<u>*tert*-butyl 2-chlorophenethylcarbamate (**2d**)</u>

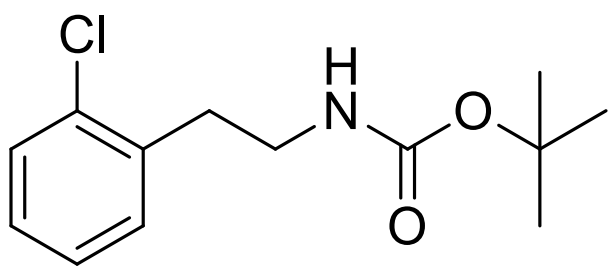

^{1}H NMR ($CDCl_3$, 600 MHz) δ [ppm]: 7.25 (m, 1H, Cl-C-CH$_{meta}$), 7.12 (m, 1H, CH$_{meta}$), 7.09 (m, 1H, CH$_{ortho}$), 7.07 (m, 1H, CH$_{para}$), 3.30 (t, J = 6.54 Hz, 2H, N-CH_2), 2.85 (t, J = 6.90 Hz, 2H, Ar-CH_2), 1.34 (s, 9H, C-CH_3).

^{13}C HMR ($CDCl_3$, 150 MHz) δ [ppm]: 155.9 (s), 136.7 (s), 134.2 (s), 131.0 (s), 129.5 (s), 127.9 (s), 126.87 (s), 79.1 (s), 77.2 (t, J = 32.70 Hz, $CDCl_3$), 40.2 (s), 33.9 (s), 28.4 (s).

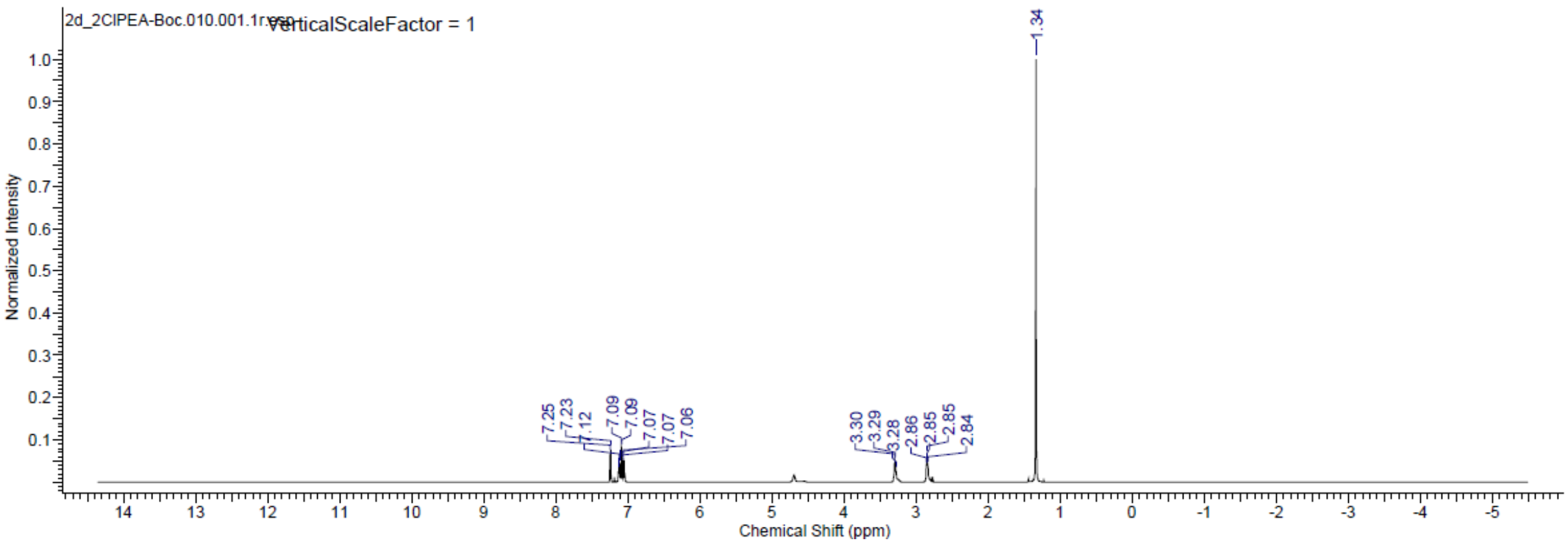

Figure S7. ^{1}H-NMR spectrum of **2d**.

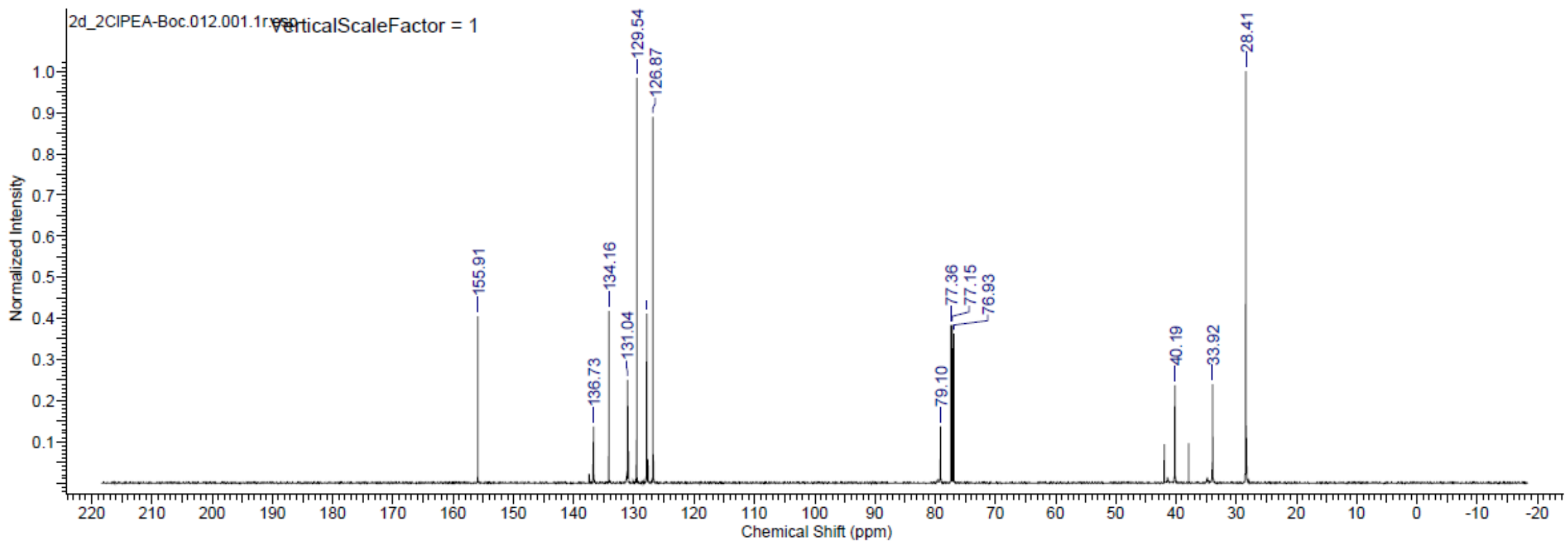

Figure S8. ^{13}C-NMR spectrum of **2d**.

<u>*tert*-butyl 3-chlorophenethylcarbamate (**2e**)</u>

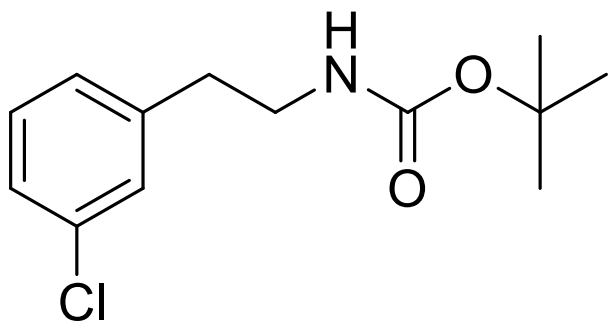

^{1}H NMR ($CDCl_3$, 600 MHz) δ [ppm]: 7.20 (s, 1H, Cl-C-CH_{ortho}), 7.18 (t, J = 1.63 Hz, 1H, CH_{meta}), 7.16 (s, 1H, Cl-C-CH_{para}), 7.06 (d, J = 7.08 Hz 1H, C-CH_{ortho}-C), 3.33 (t, J = 6.36 Hz, 2H, N-CH_2-C), 2.76 (t, J = 6.90 Hz, 2H, Ar-CH_2), 1.42 (s, 9H, C-CH_3).

^{13}C HMR ($CDCl_3$, 150 MHz) δ [ppm]: 155.7 (s), 146.7 (s), 141.0 (s), 134.2 (s), 129.7 (s), 128.8 (s), 126.5 (s), 85.1 (s), 77.1 (t, J = 31.61 Hz, $CDCl_3$), 41.5 (s), 35.8 (s), 28.3 (s).

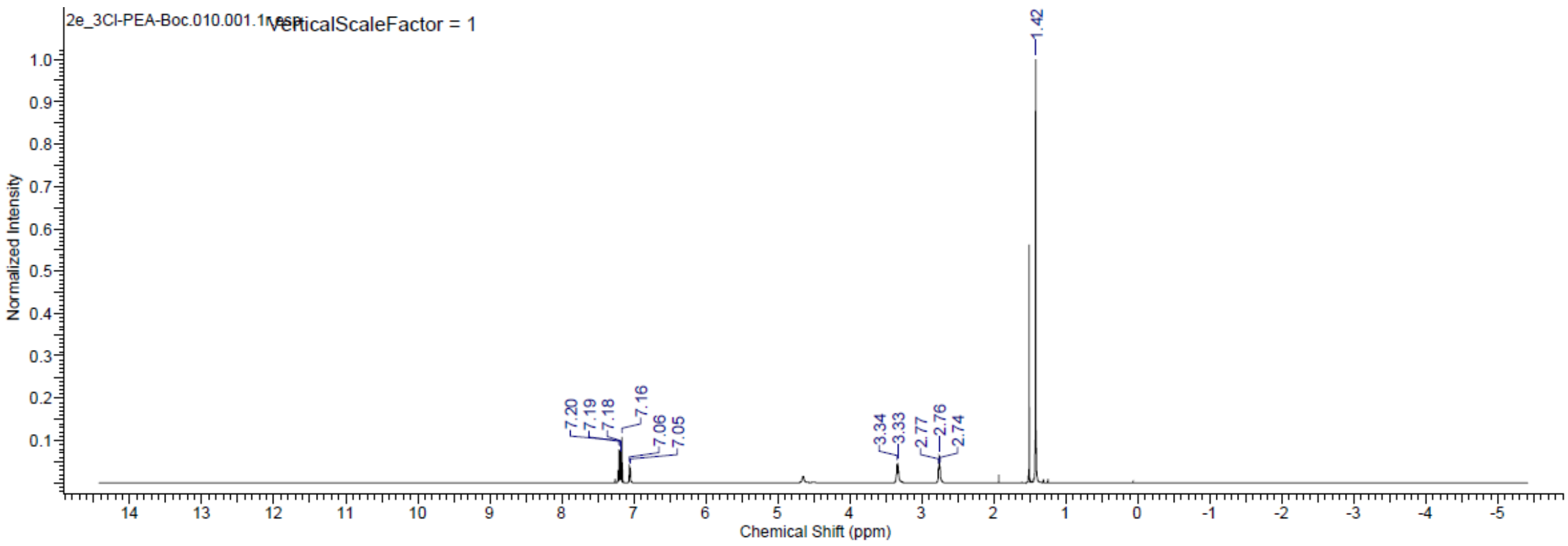

Figure S9. ^{1}H-NMR spectrum of **2e**.

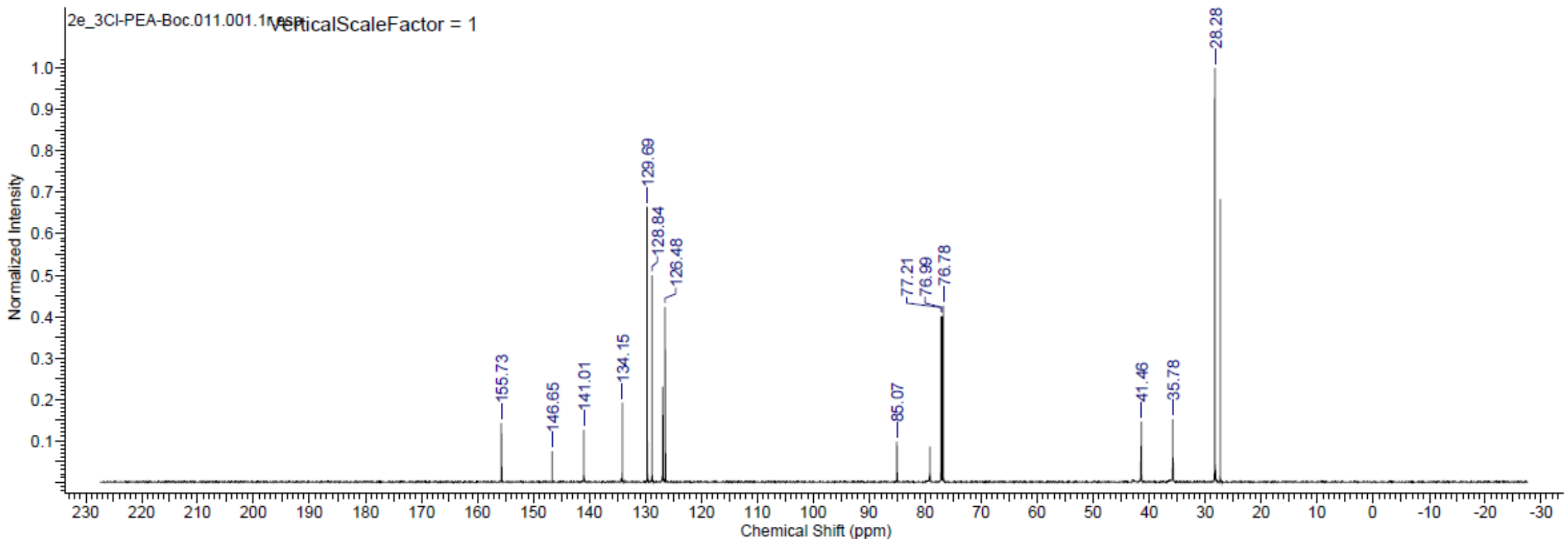

Figure S10. ^{13}C-NMR spectrum of **2e**.

<u>*tert*-butyl 3,4-dichlorophenethylcarbamate (**2f**)</u>

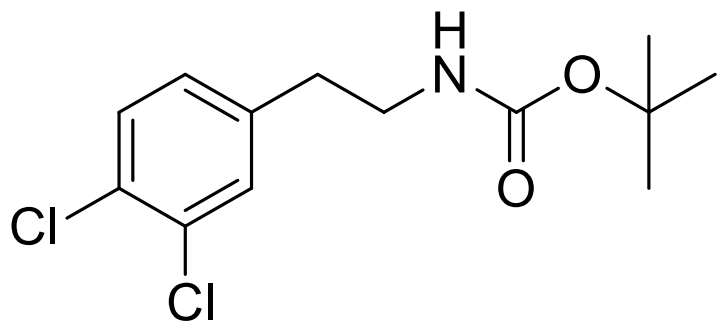

^{1}H NMR ($CDCl_3$, 600 MHz) δ [ppm]: 7.38 (d, *J* = 8.17 Hz, 1H, Cl-C-CH_{meta}), 7.30 (s, 1H, C-CH_{ortho}), 7.05 (d, *J* = 7.99 Hz, 1H, Cl-C-CH_{ortho}), 3.36 (t, *J* = 6.18 Hz, 2H, N-CH_2-C), 2.78 (t, *J* = 6.90 Hz, 2H, Ar-CH_2), 1.45 (s, 9H, C-CH_3).

^{13}C HMR ($CDCl_3$, 150 MHz) δ [ppm]: 155.8 (s), 139.3 (s), 132.4 (s), 130.8 (s), 130.5 (s), 128.3 (s), 79.5 (s), 77.1 (t, *J* = 31.61 Hz, $CDCl_3$), 41.5 (s), 35.4 (s), 28.4 (s).

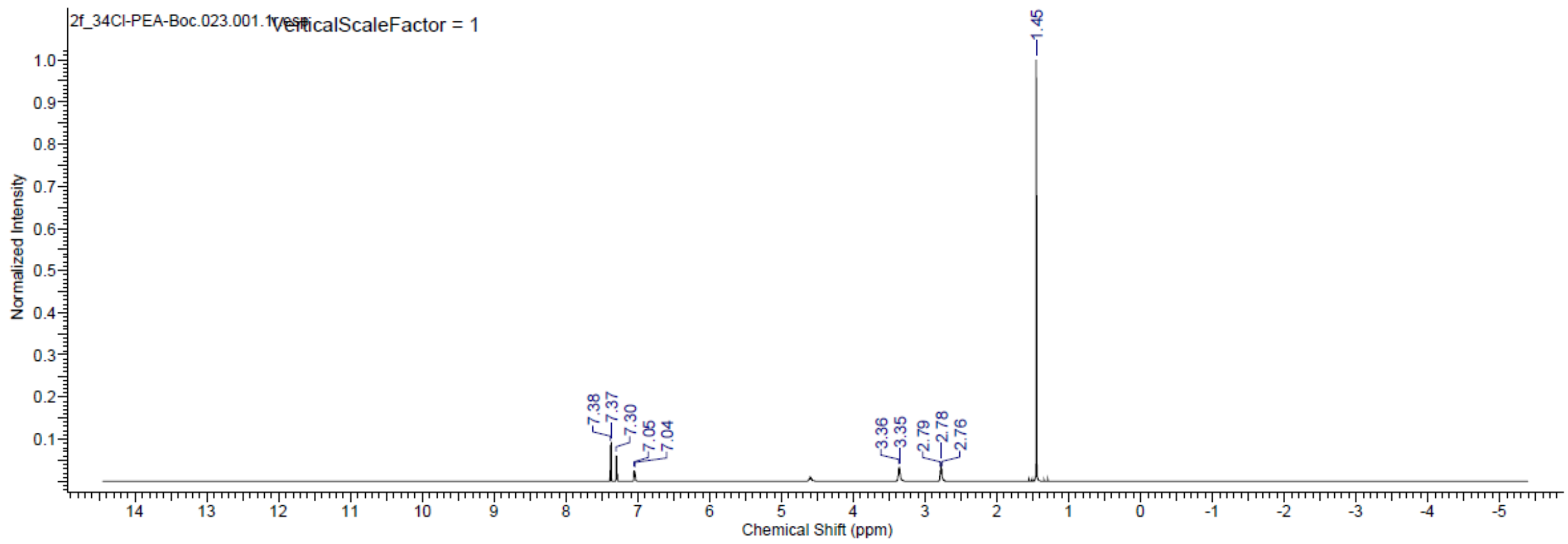

Figure S11. ^{1}H-NMR spectrum of **2f**.

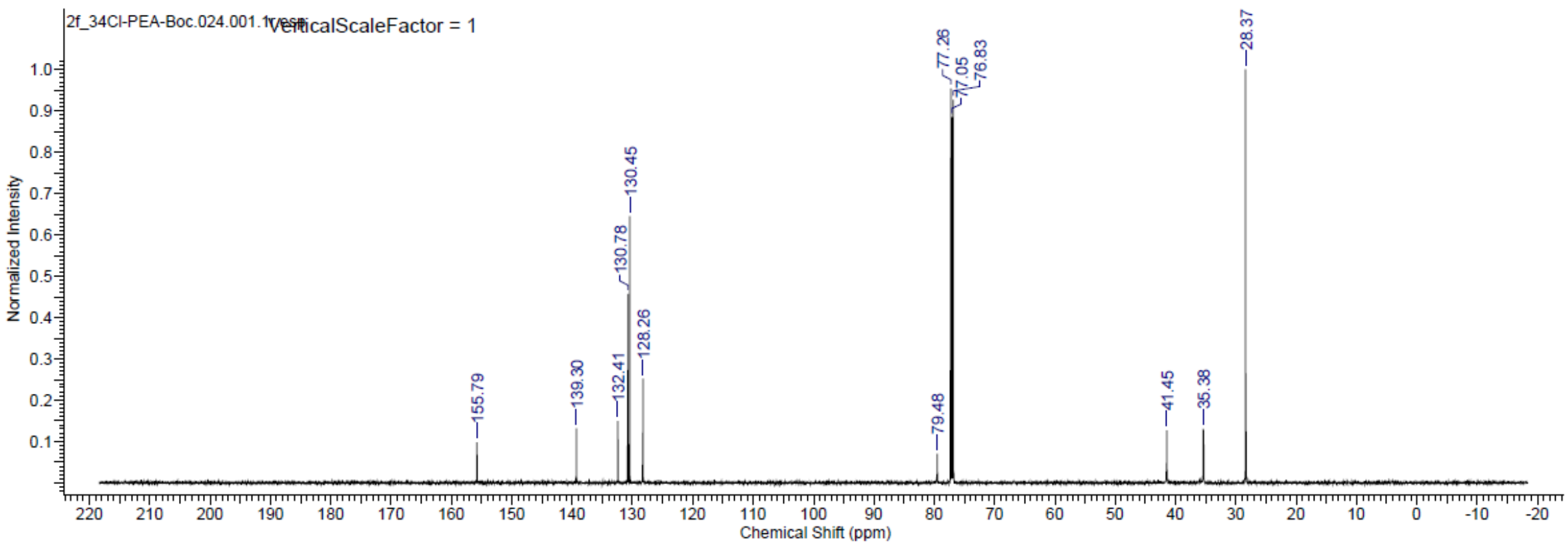

Figure S12. ^{13}C-NMR spectrum of **2f**.

<u>*tert*-butyl 4-hydroxyphenethylcarbanate (**2g**)</u>

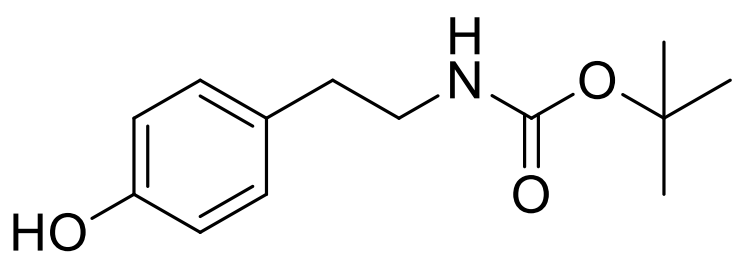

^{1}H NMR ($CDCl_3$, 600 MHz) δ [ppm]: 6.91 (d, *J* = 8.17 Hz, 2H, CH_{ortho}), 6.71 (d, *J* = 8.54 Hz, 2H, CH_{meta}), 4.67 (s, 1H, OH), 3.23 (t, *J* = 6.36 Hz, 2H, NH-CH_2-C), 2.60 (t, *J* = 6.90 Hz, 2H, Ar-CH_2), 1.36 (s, 9H, C-CH_3).

^{13}C HMR ($CDCl_3$, 150 MHz) δ [ppm]: 155.4 (s), 155.0 (s), 130.1 (s), 129.8 (s), 115.6 (s), 79.8 (s), 77.1 (t, *J* = 32.70 Hz, $CDCl_3$), 42.1 (s), 35.2 (s), 28.4 (s).

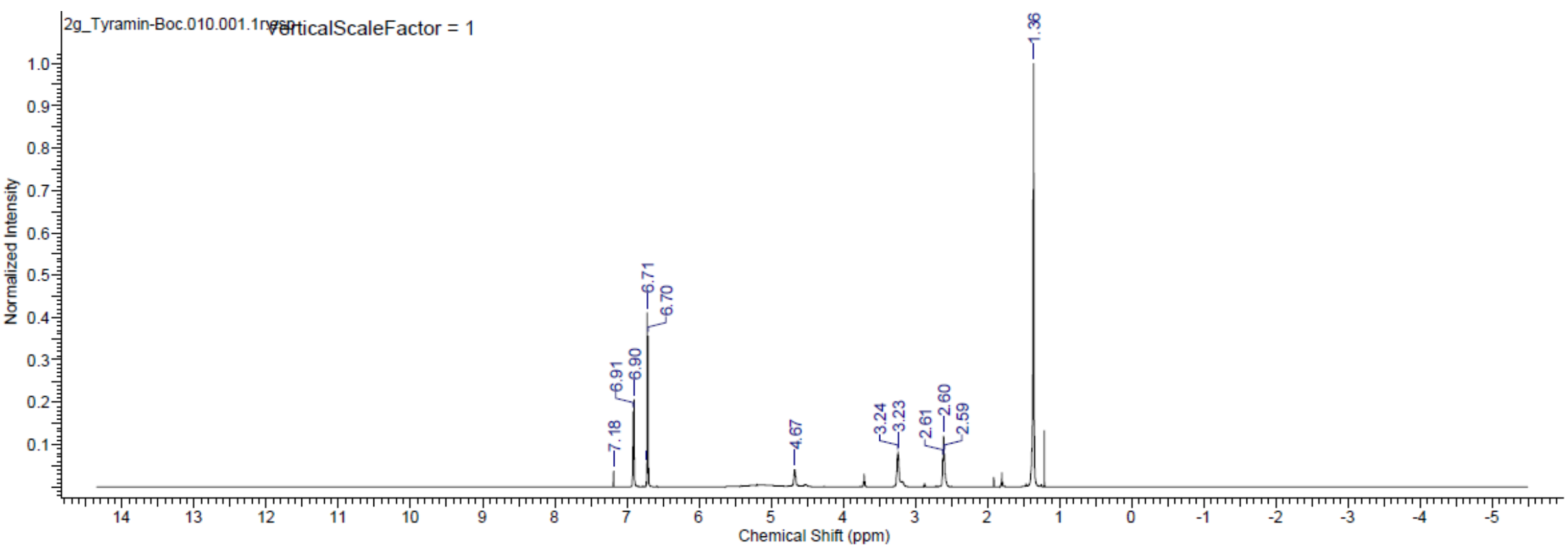

Figure S13. ^{1}H-NMR spectrum of **2g**.

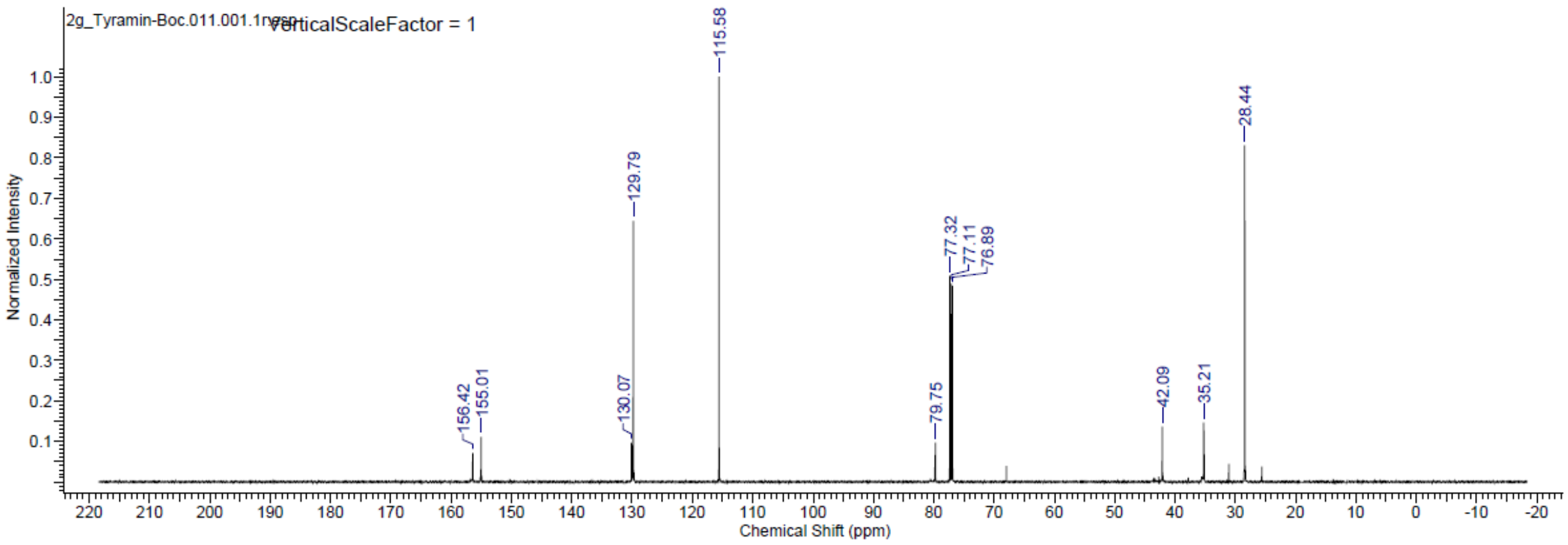

Figure S14. ^{13}C-NMR spectrum of **2g**.

tert-butyl 3,4-dihydroxyphenethylcarbamate (**2h**)

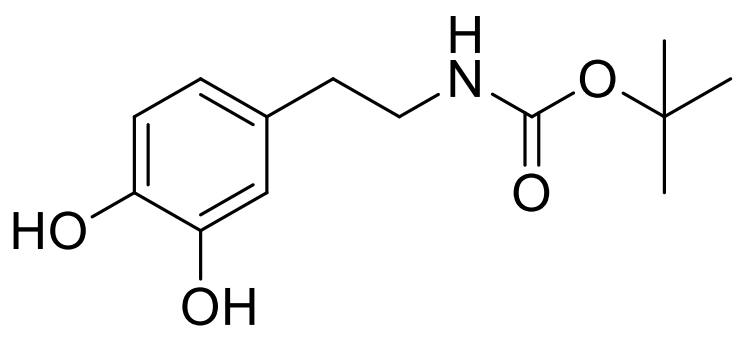

^1H NMR (DMSO-d_6, 600 MHz) δ [ppm]: 8.63 (s, 1H, C-NH-C), 6.66 (d, J = 7.99 Hz, 1H, CH_{meta}), 6.60 (s, 1H, OH-C-CH_{ortho}), 6.45 (d, J = 6.18 Hz, 1H, CH_{ortho}), 5.76 (s, 2H, Ar-OH), 3.40 (s, 2H, H_2O), 3.07 (t, J = 8.72 Hz, 2H, N-CH_2), 2.55 (t, J = 7.27 Hz, 2H, Ar-CH_2), 2.52 (s, 6H, DMSO-d_6), 1.51 (s, 9H, C-CH_3).

^{13}C HMR (DMSO-d_6, 150 MHz) δ [ppm]: 155.4 (s), 146.2 (s), 145.0 (s), 143.4 (s), 130.1 (s), 119.1 (s), 115.4 (s), 85.4 (s), 42.0 (s), 39.5 (h, J = 21.13 Hz, DMSO-d_6), 35.0 (s), 26.8 (s).

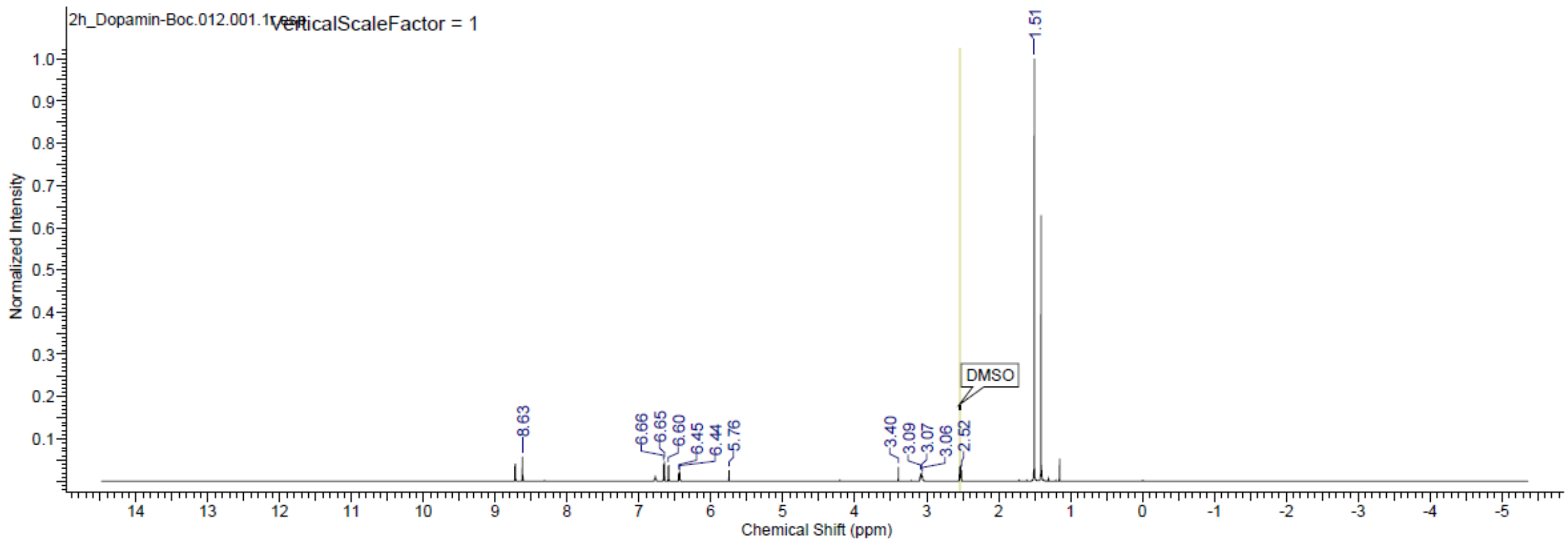

Figure S15. ^1H-NMR spectrum of **2h**.

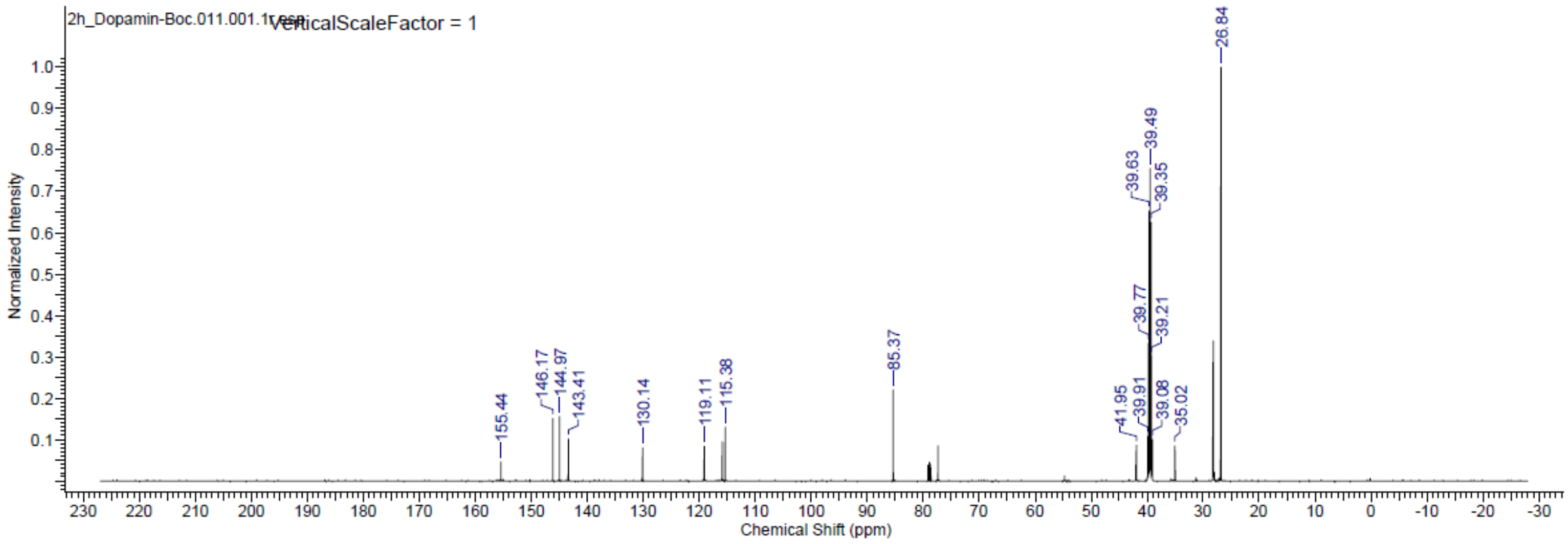

Figure S16. ^{13}C-NMR spectrum of **2h**.

<u>*tert*-butyl 4-(trifluoromethyl)phenethylcarbamate (**2i**)</u>

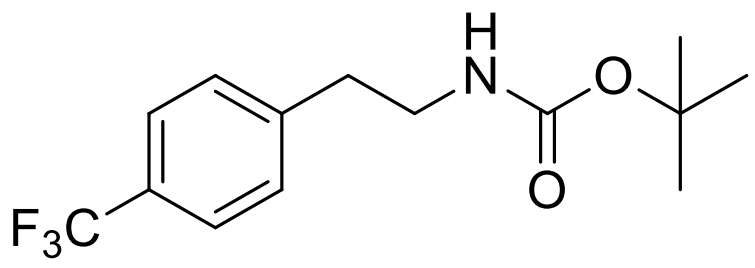

^{1}H NMR ($CDCl_3$, 600 MHz) δ [ppm]: 7.48 (d, *J* = 8.17 Hz, 2H, CH_{meta}), 7.23 (d, *J* = 7.81 Hz, 2H, CH_{ortho}), 3.32 (dd, *J* = 6.36 Hz, 2H, NH-CH_2), 2.78 (t, *J* = 6.72 Hz, 2H, Ar-CH_2), 1.35 (s, 9H, C-CH_3).

^{13}C HMR ($CDCl_3$, 150 MHz) δ [ppm]: 155.8 (s), 143.2 (s), 129.2 (s), 125.5 (s), 125.4 (s), 79.4 (s), 77.0 (t, *J* = 31.61 Hz, $CDCl_3$), 41.5 (s), 36.1 (s), 28.4 (s).

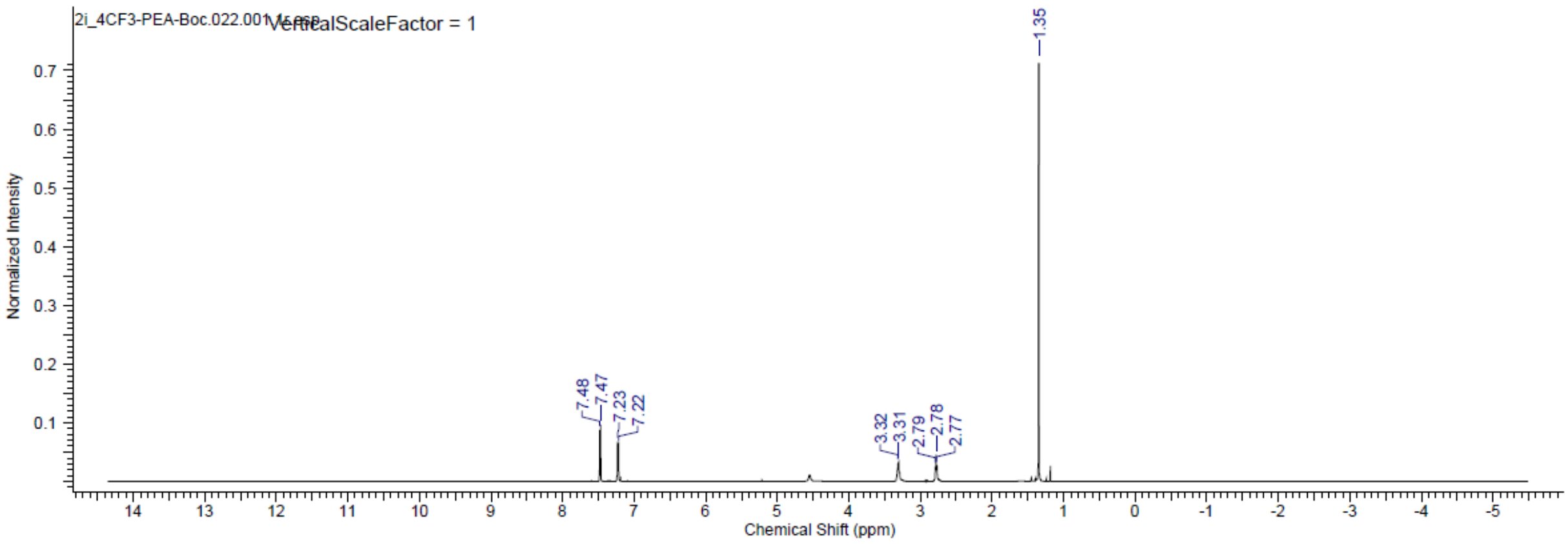

Figure S17. ^{1}H-NMR spectrum of **2i**.

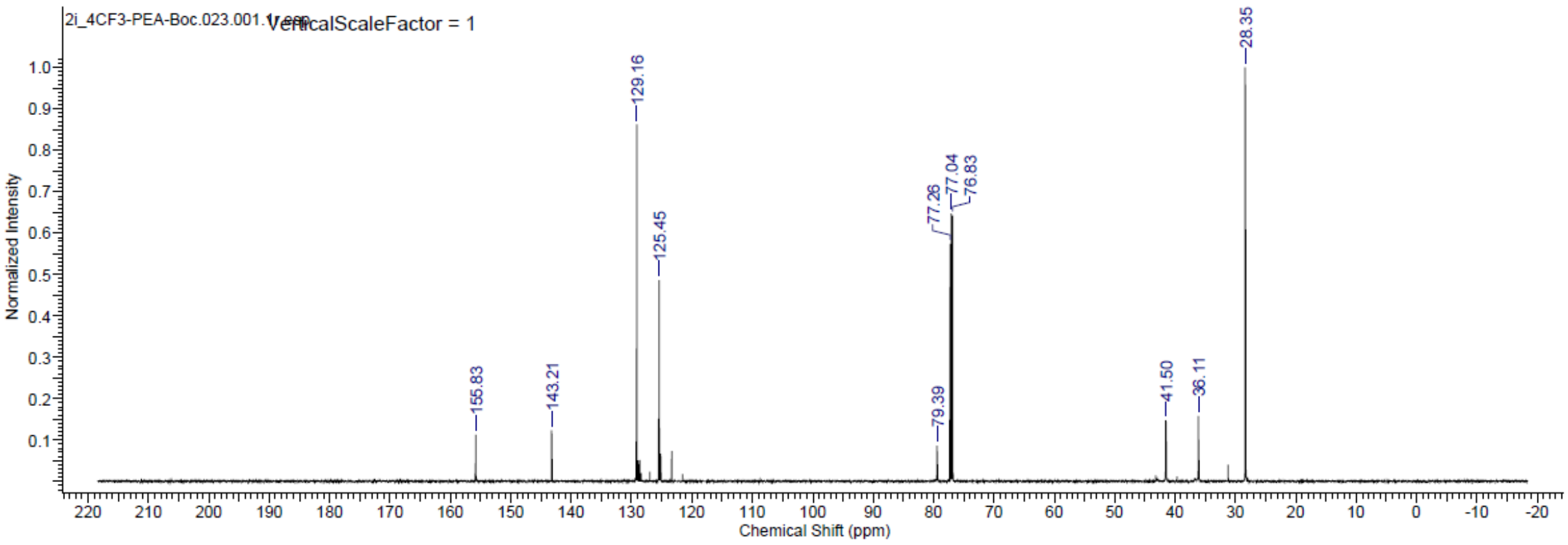

Figure S18. ^{13}C-NMR spectrum of **2i**.

<u>*tert*-butyl 4-aminophenethylcarbamate (**2j**)</u>

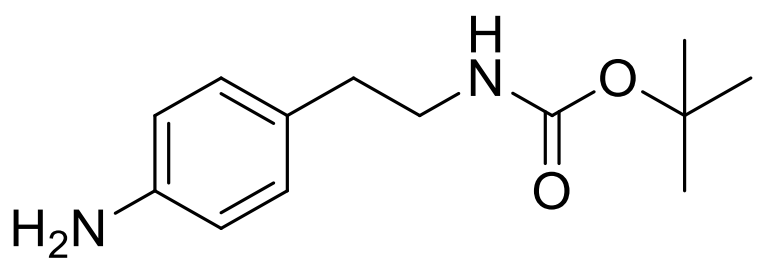

^1H NMR ($CDCl_3$, 600 MHz) δ [ppm]: 6.95 (d, J = 7.99 Hz, 2H, CH_{ortho}), 6.62 (d, J = 8.36 Hz, 2H, CH_{meta}), 5.38 (s, 2H, Ar-NH_2), 3.30 (t, J = 6.18 Hz, 2H, N-CH_2), 2.65 (t, J = 6.72 Hz, 2H, Ar-CH_2), 1.41 (s, 9H, C-CH_3).

^{13}C HMR ($CDCl_3$, 150 MHz) δ [ppm]: 155.8 (s), 144.7 (s), 129.5 (s), 129.2 (s), 115.2 (s), 79.0 (s), 77.0 (t, J = 31.69 Hz, $CDCl_3$), 41.9 (s), 35.1 (s), 28.3 (s).

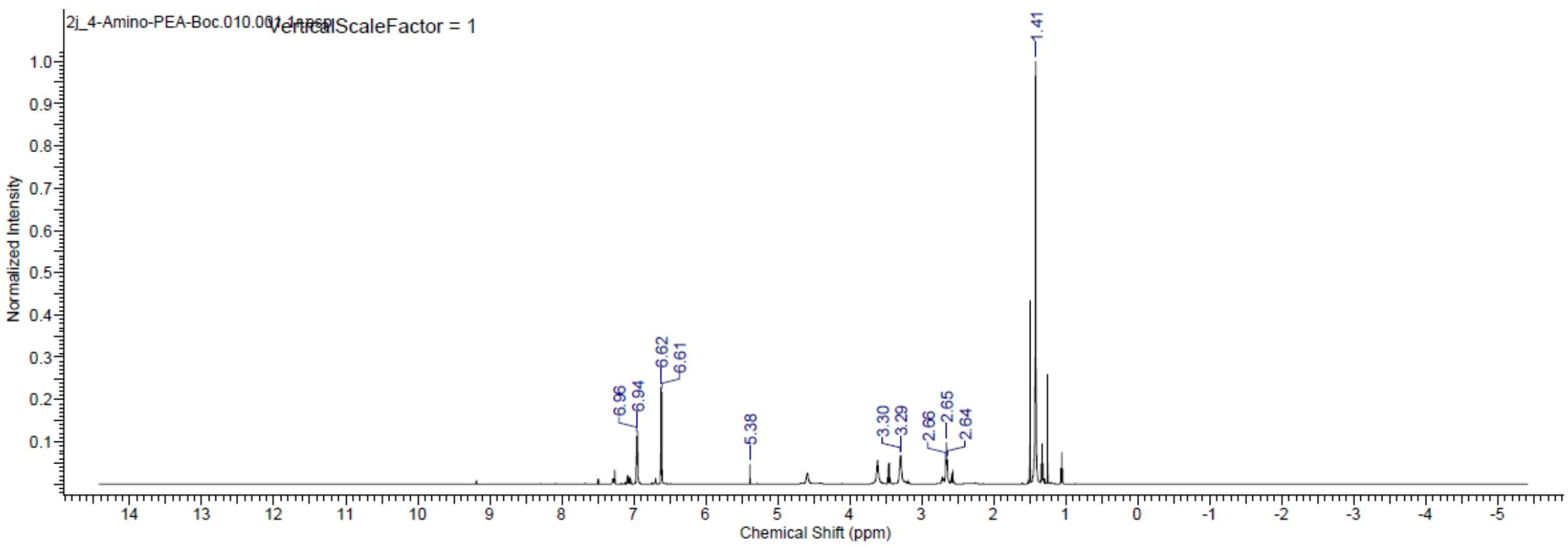

Figure S19. ^1H-NMR spectrum of **2j**.

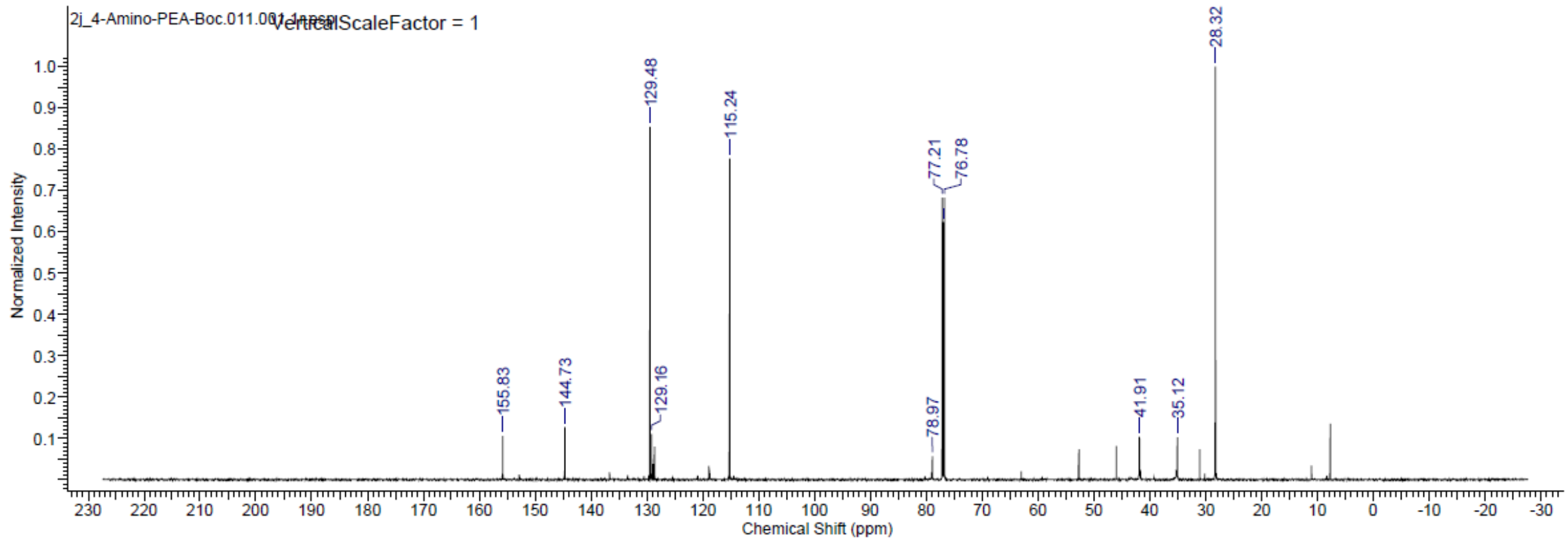

Figure S20. ^{13}C-NMR spectrum of **2j**.

<u>*tert*-butyl 4-(methylsulfonamido)phenethylcarbamate (**2k**)</u>

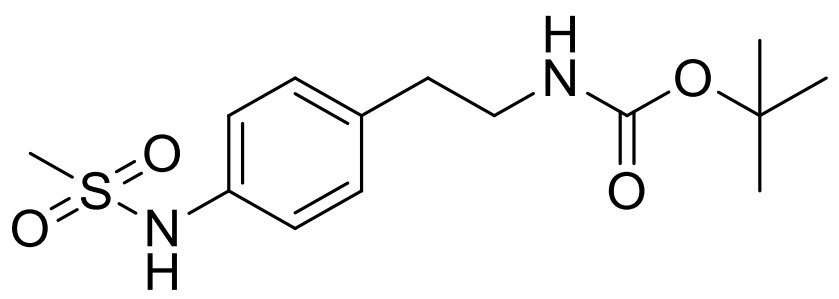

^{1}H NMR ($CDCl_3$, 600 MHz) δ [ppm]: 7.20 (m, 2H, CH_{meta}), 7.11 (d, J = 1.63 Hz, 2H, CH_{ortho}), 4.06 (s, 1H, S-NH), 3.28 (t, J = 6.18 Hz, 2H, N-CH_2), 2.92 (s, 3H, S-CH_3), 2.70 (t, J = 6.90 Hz, 2H, Ar-CH_2), 1.36 (s, 9H, C-CH_3).

^{13}C HMR ($CDCl_3$, 150 MHz) δ [ppm]: 155.9 (s), 135.2 (s), 130.0 (s), 129.3 (s), 121.4 (s), 79.4 (s), 77.0 (t, J = 31.61 Hz, $CDCl_3$), 41.7 (s), 39.3 (s), 35.6 (s), 28.4 (s).

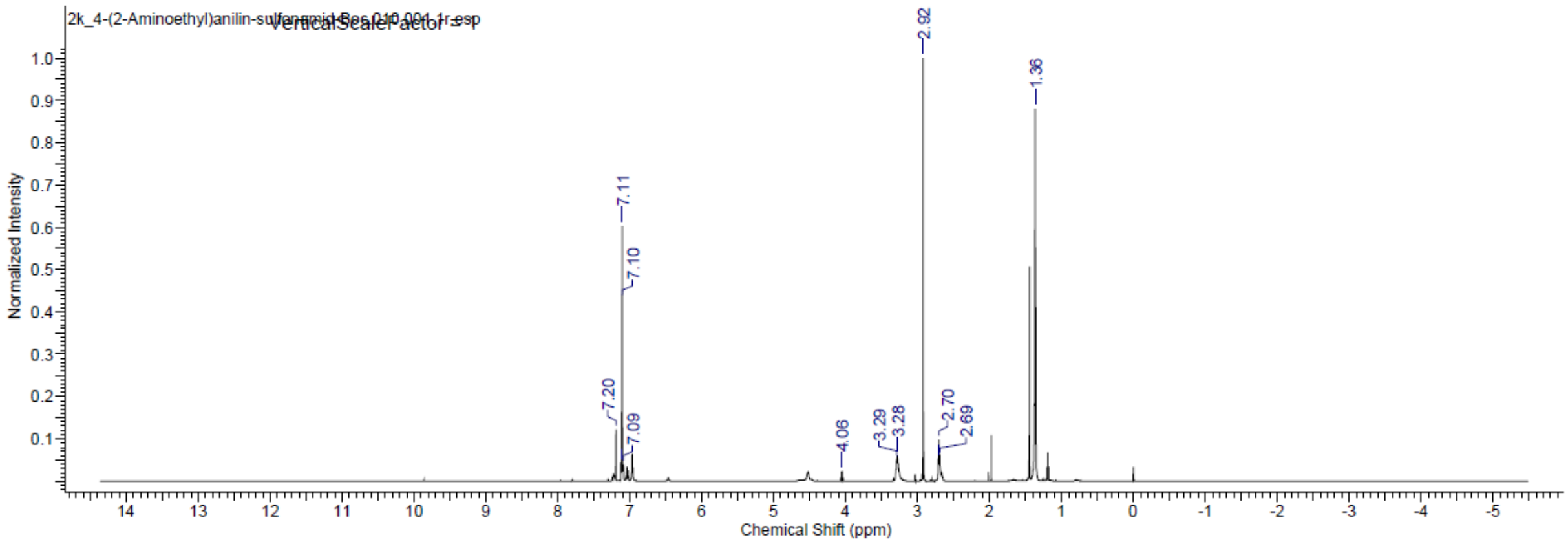

Figure S21. ^{1}H-NMR spectrum of **2k**.

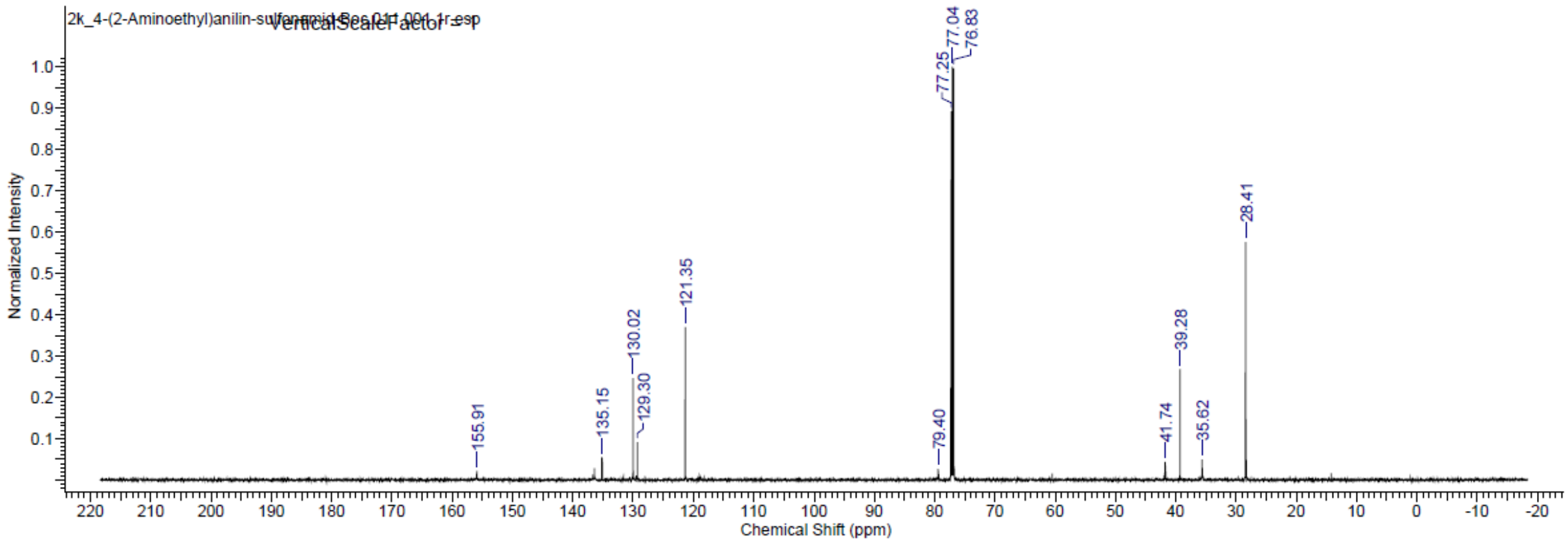

Figure S22. ^{13}C-NMR spectrum of **2k**.

<u>*tert*-butyl (2-(naphthalen-2-yl)ethyl)carbamate (**2l**)</u>

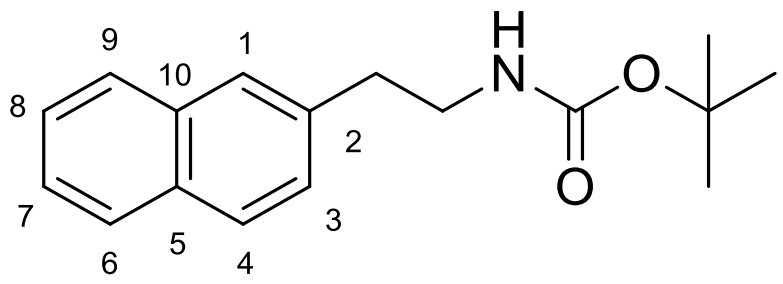

^{1}H NMR ($CDCl_3$, 400 MHz) δ [ppm]: 7.77 (m, 1H, C^6H), 7.75 (m, 1H, C^9H), 7.73 (m, 1H, C^4H), 7.59 (s, 1H, C^1H), 7.42 (m, 1H, C^8H), 7.40 (m, 1H, C^7H), 7.30 (m, 1H, C^3H), 3.08 (dd, J = 7.34 Hz, 2H, N-CH_2), 2.91 (t, J = 6.97 Hz, 2H, Ar-CH_2), 1.38 (s, 9H, C-CH_3).

^{13}C HMR ($CDCl_3$, 100 MHz) δ [ppm]: 155.9 (s), 136.5 (s), 133.5 (s), 132.2 (s), 128.2 (s), 127.6 (s), 127.4 (s), 127.11 (s), 126.0 (s), 125.4 (s), 79.1 (s) 77.2 (t, J = 32.28 Hz, $CDCl_3$), 46.1 (s), 28.4 (s), 8.8 (s).

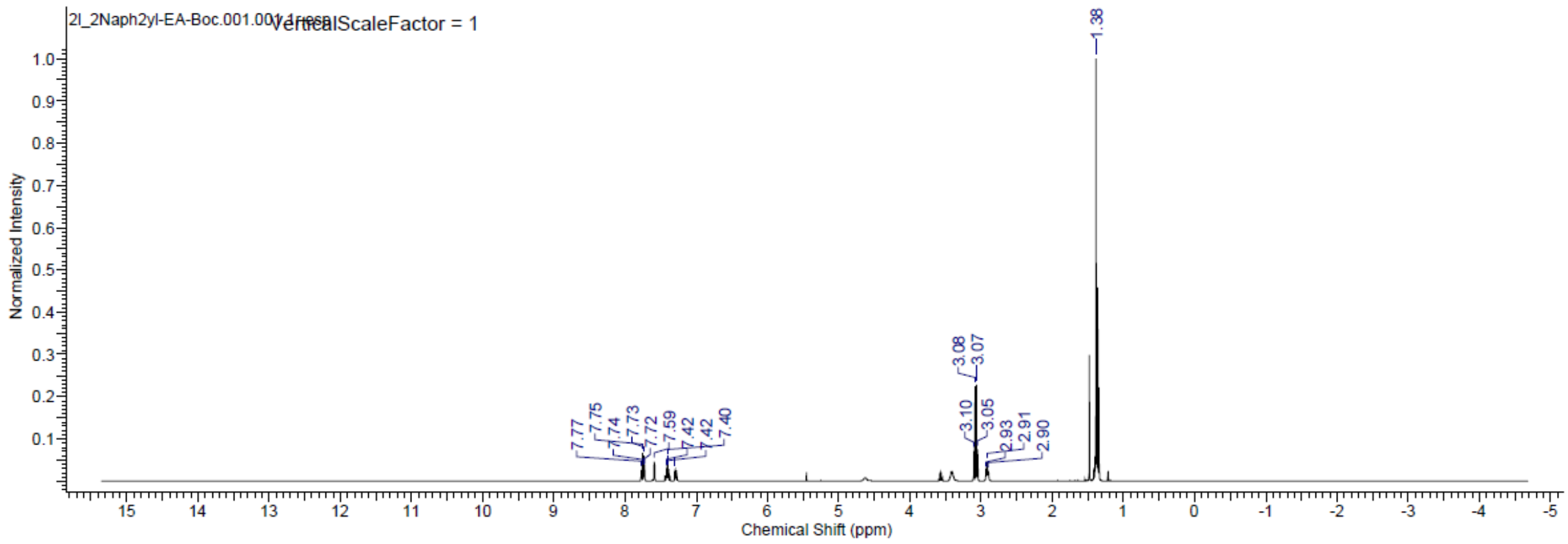

Figure S23. ^{1}H-NMR spectrum of **2l**.

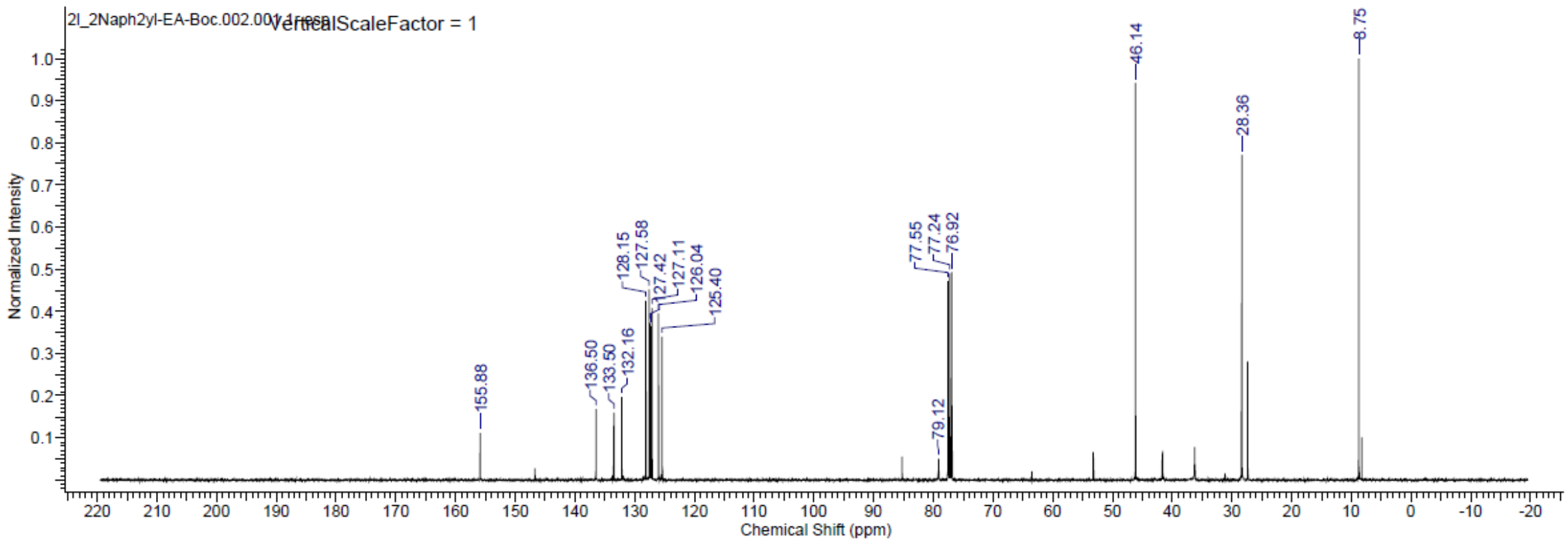

Figure S24. ^{13}C-NMR spectrum of **2l**.

<u>*tert*-butyl (2-(naphthalen-1-yl)ethyl)carbamate (**2m**)</u>

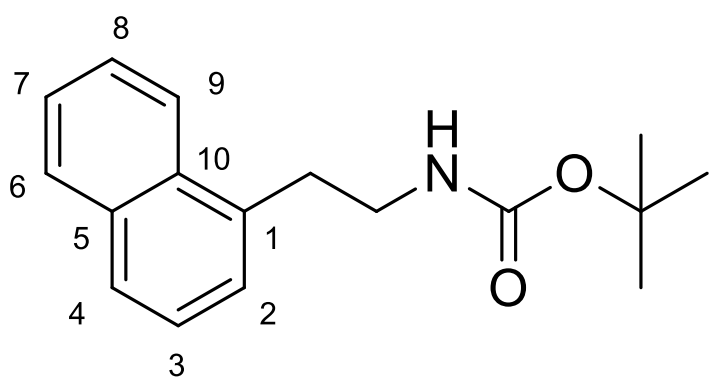

^{1}H NMR ($CDCl_3$, 400 MHz) δ [ppm]: 8.14 (d, *J* = 8.31 Hz, 1H, C^{9}H), 7.89 (d, *J* = 7.70 Hz, 1H, C^{6}H), 7.78 (d, *J* = 8.19 Hz, 1H, C^{4}H), 7.55 (m, 1H, C^{8}H), 7.52 (m, 1H, C^{7}H), 7.44 (t, *J* = 6.97 Hz, 1H, C^{3}H), 7.36 (d, *J* = 6.85 Hz, 1H, C^{2}H), 3.54 (t, *J* = 6.60 Hz, 2H, Ar-CH_2), 3.31 (t, *J* = 6.72 Hz, 2H, N-CH_2), 1.57 (s, 9H, C-CH_3).

^{13}C HMR ($CDCl_3$, 100 MHz) δ [ppm]: 156.0 (s), 134.0 (s), 132.1 (s), 128.8 (s), 127.2 (s), 126.8 (s), 126.1 (s), 125.7 (s), 125.6 (s), 123.8 (s), 85.2 (s), 77.2 (t, *J* = 32.28 Hz, $CDCl_3$),41.4 (s), 33.4 (s), 27.4 (s).

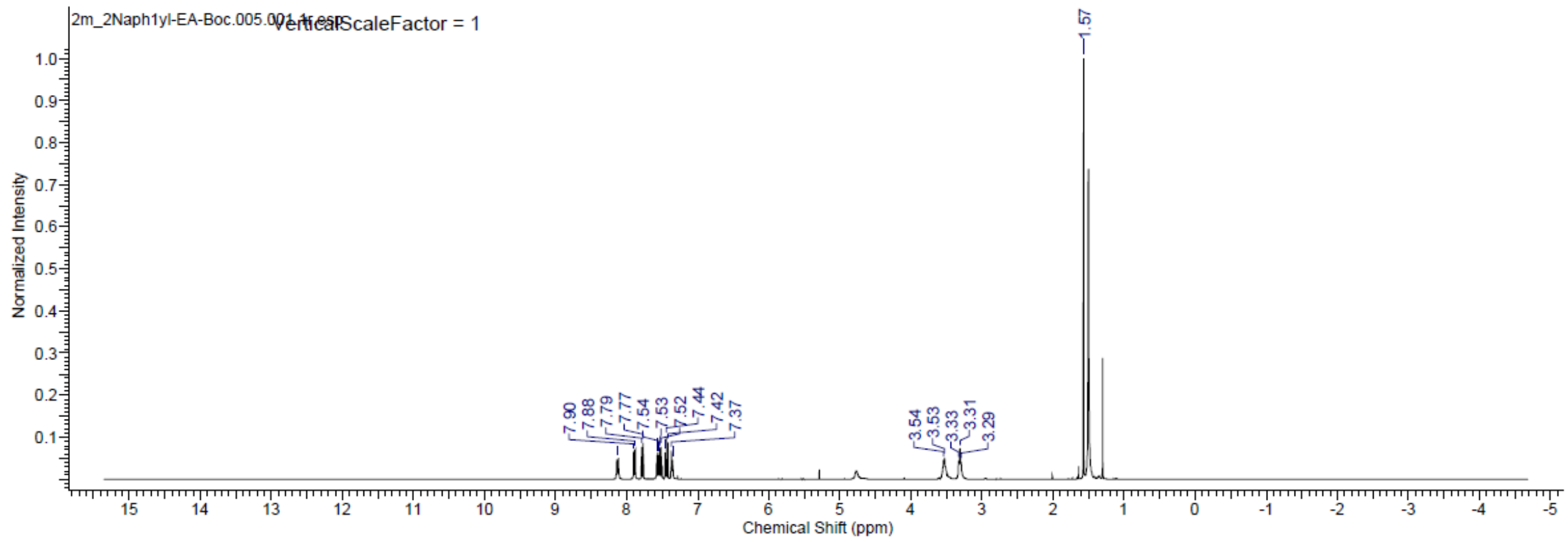

Figure S25. ^{1}H-NMR spectrum of **2m**.

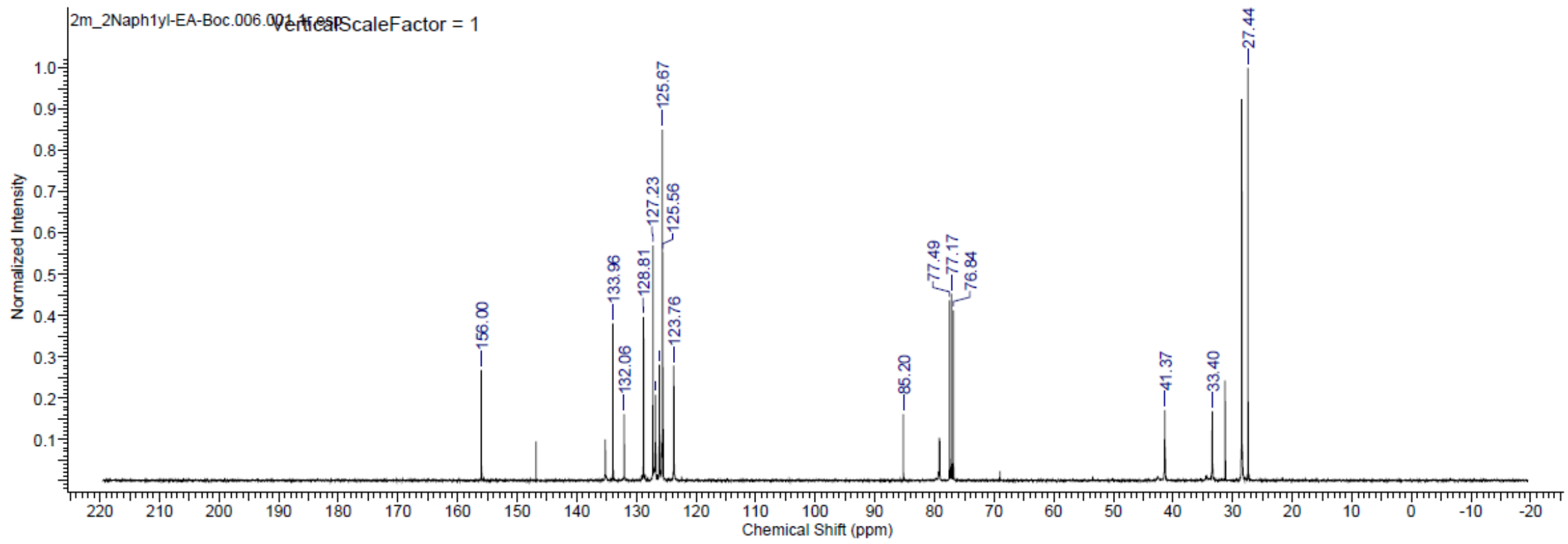

Figure S26. ^{13}C-NMR spectrum of **2m**.

<u>*tert*-butyl (2-hydroxy-2-phenylethyl)carbamate (**3a**)</u>

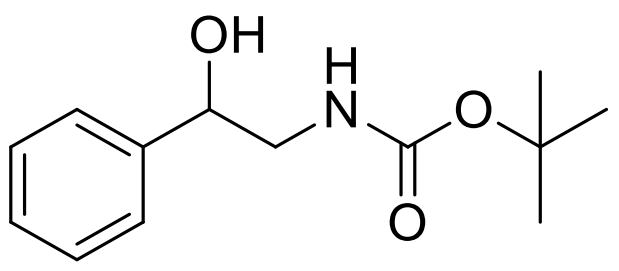

^{1}H NMR ($CDCl_3$, 400 MHz) δ [ppm]: 7.40 (m, 1H, Ar-H_{para}), 7.37 (s, 1H, $CDCl_3$), 7.36 (m, 2H, Ar-H_{meta}), 7.33 (m, 2H, Ar-H_{ortho}), 4.80 (t, *J* = 4.77 Hz, 1H, OH-CH-C), 3.48 (s, 1H, OH), 3.24 (d, *J* = 5.87 Hz, 2H, N-CH_2-C), 1.46 (s, 9H, C-CH_3).

^{13}C HMR ($CDCl_3$, 100 MHz) δ [ppm]: 157.0 (s), 141.9 (s), 128.5 (s), 127.7 (s), 125.9 (s), 79.8 (s), 77.1 (t, *J* = 31.55 Hz, $CDCl_3$), 73.8 (s), 48.3 (s), 28.4 (s).

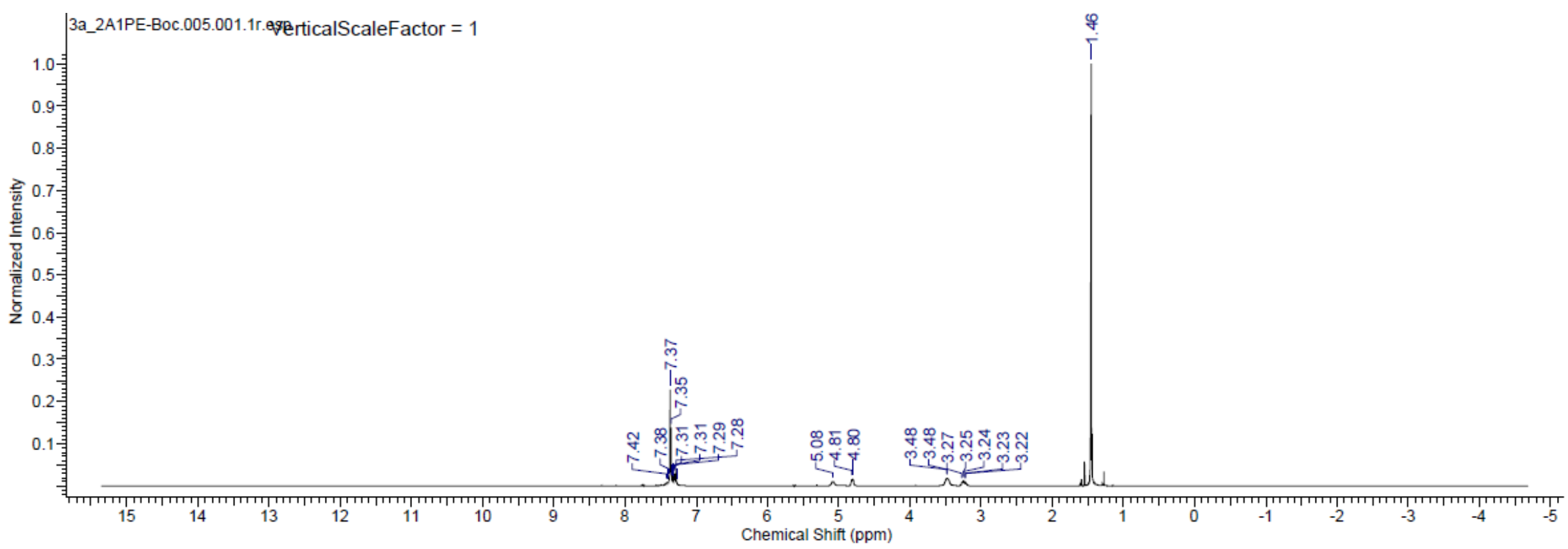

Figure S27. ^{1}H-NMR spectrum of **3a**.

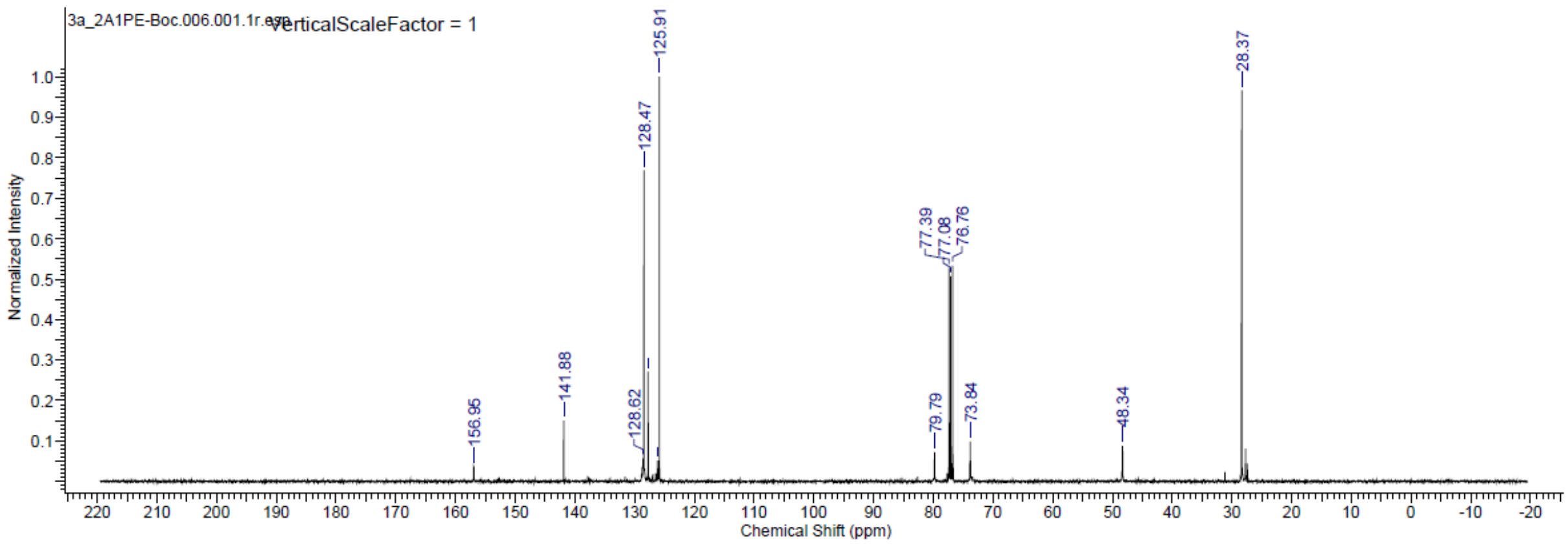

Figure S28. ^{13}C-NMR spectrum of **3a**.

<u>*tert*-butyl (2-(3-chlorophenyl)-2-hydroxyethyl)carbamate (**3e**)</u>

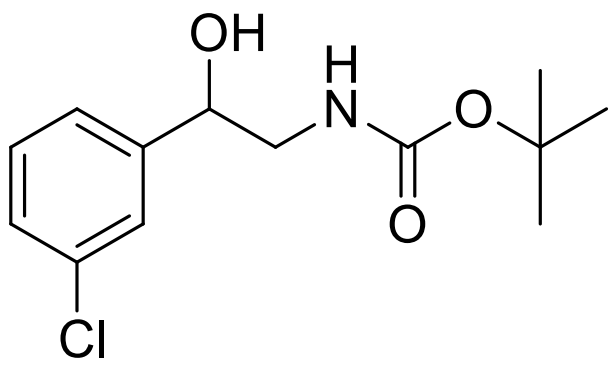

^{1}H NMR ($CDCl_3$, 600 MHz) δ [ppm]: 7.30 (s, 1H, Cl-C-CH_{ortho}), 7.22 (d, *J* = 2.54 Hz, 1H, Ar-H_{para}), 7.19 (m, 1H, Ar-H_{meta}), 7,21 (m, 1H, Ar-H_{ortho}), 4.73 (t, *J* = 5.09 Hz, 1H, OH-CH-C), 3.38 (d, *J* = 6.63 Hz, 1H, N-CH_2-C), 3.14 (m, 1H, N-CH_2-C), 1.36 (s, 9H, C-CH_3).

^{13}C HMR ($CDCl_3$, 150 MHz) δ [ppm]: 157.1 (s), 143.9 (s), 134.4 (s), 130.1 (s), 129.7 (s), 127.8 (s), 126.1 (s), 124.0 (s), 80.1 (s), 77.0 (t, *J* = 31.69 Hz, $CDCl_3$), 73.4 (s), 48.3 (s), 28.3 (s).

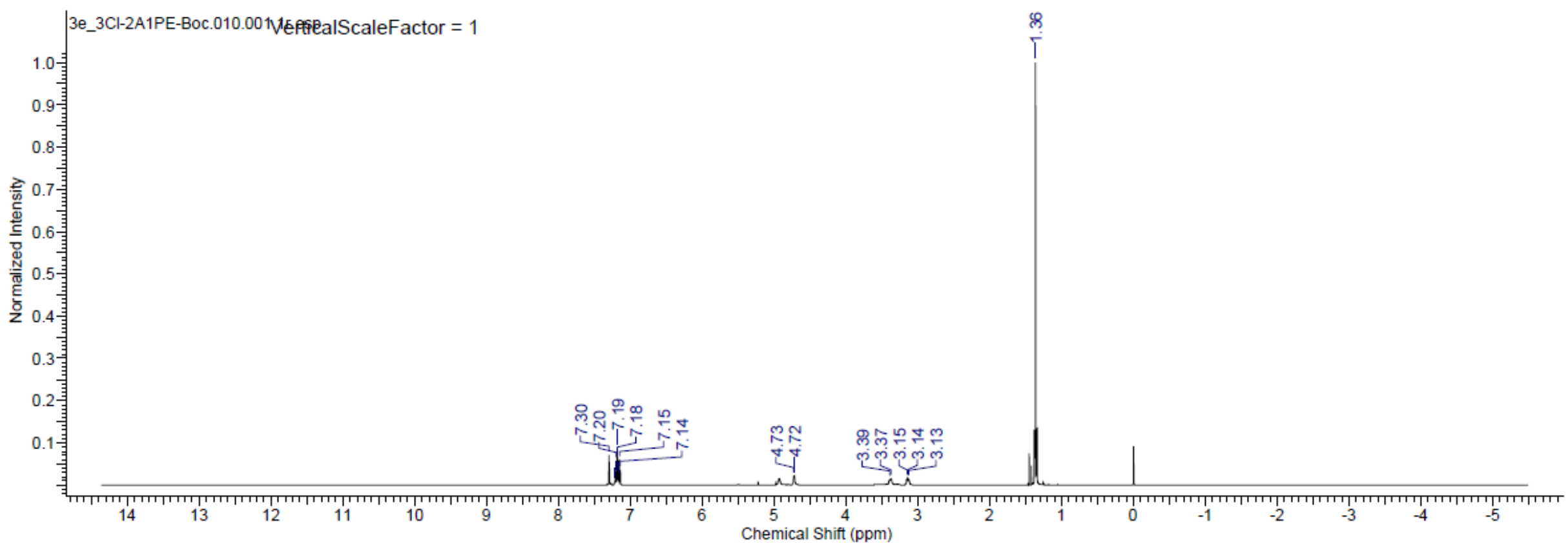

Figure S29. ^{1}H-NMR spectrum of **3e**.

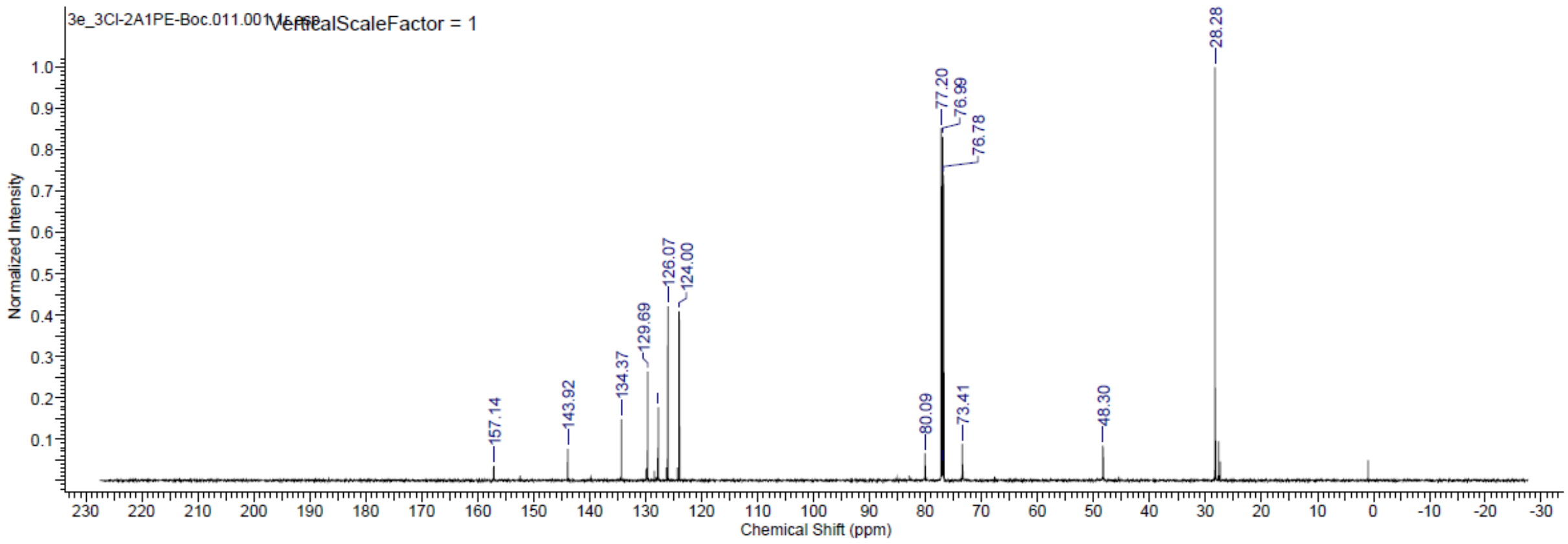

Figure S30. ^{13}C-NMR spectrum of **3e**.

<u>*tert*-butyl (2-(3,4-dichlorophenyl)-2-hydroxyethyl)carbamate (**3f**)</u>

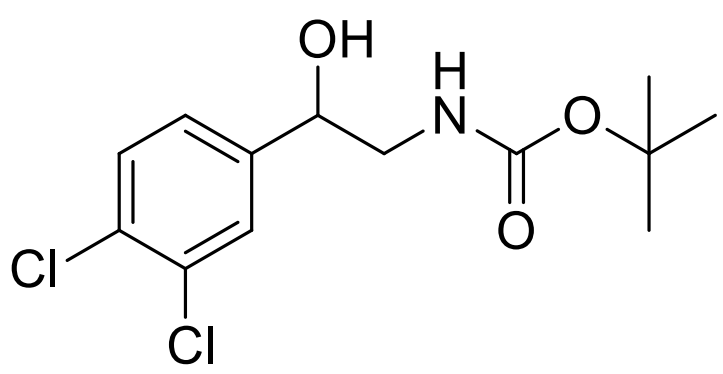

^{1}H NMR ($CDCl_3$, 600 MHz) δ [ppm]: 7.46 (s, 1H, Cl-C-CH_{ortho}), 7.40 (d, J = 8.17 Hz, 1H, Cl-C-CH_{meta}), 7.17 (d, J = 8.17 Hz, 1H, C-CH_{ortho}), 5.19 (t, J = 6.36 Hz, 1H, OH-CH-C), 3.85 (s, 1H, OH), 3.43 (dd, J = 5.09 Hz, 1H, N-CH_2-C), 3.18 (dd, J = 7.08 Hz, 1H, N-CH_2-C), 1.42 (s, 9H, C-CH_3).

^{13}C HMR ($CDCl_3$, 150 MHz) δ [ppm]: 157.2 (s), 142.2 (s), 132.6 (s), 133.2 (s), 131.5 (s), 130.4 (s), 127.9 (s), 125.2 (s), 80.3 (s), 77.1 (t, J = 32.87 Hz, $CDCl_3$), 72.9 (s), 48.3 (s), 28.3 (s).

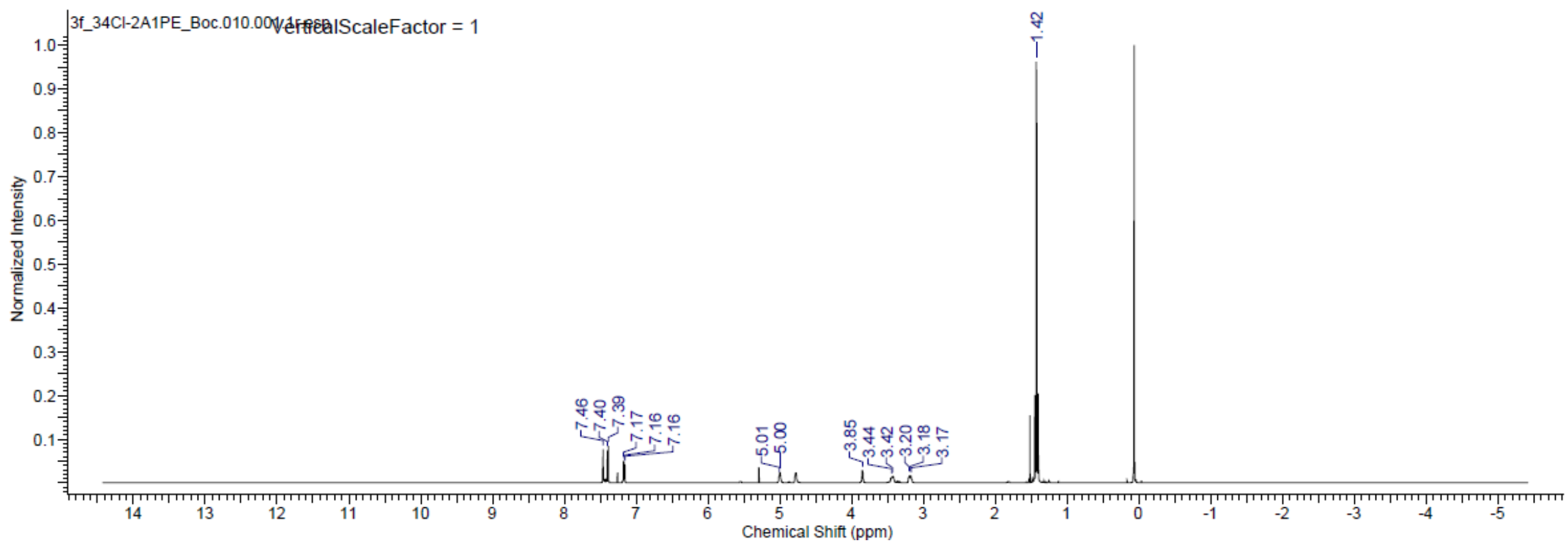

Figure S31. ^{1}H-NMR spectrum of **3f**.

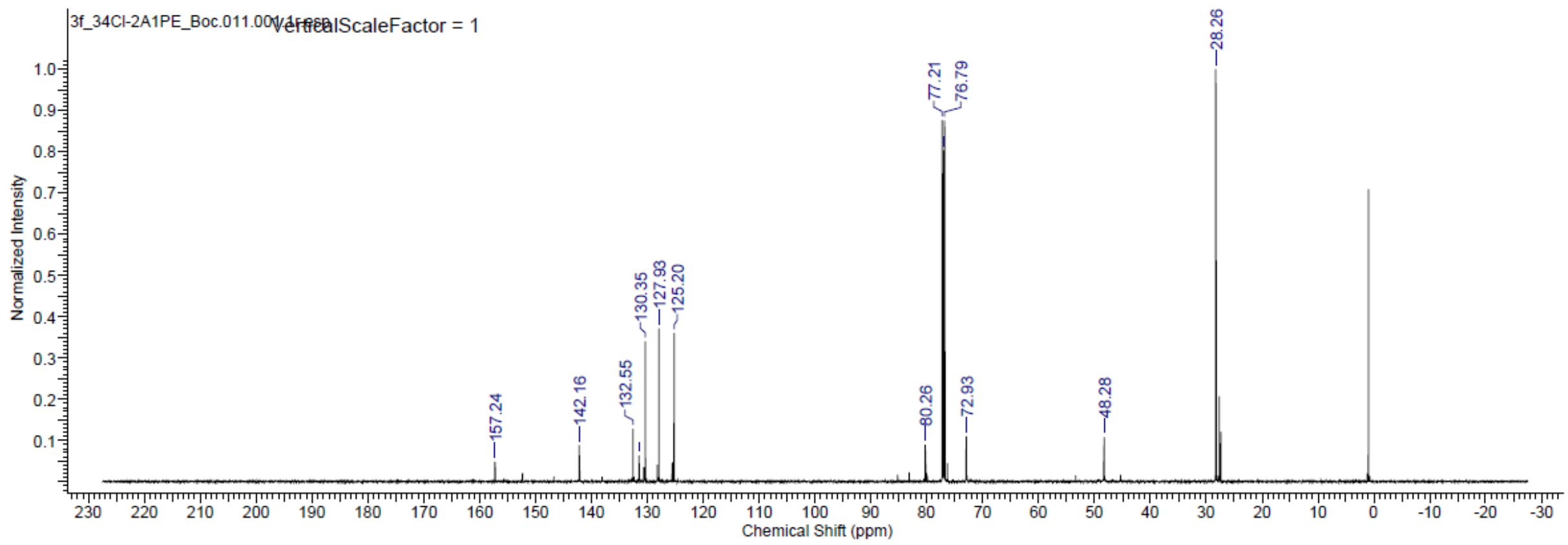

Figure S32. ^{13}C-NMR spectrum of **3f**.

2-amino-1-phenylethanol (**4a**)

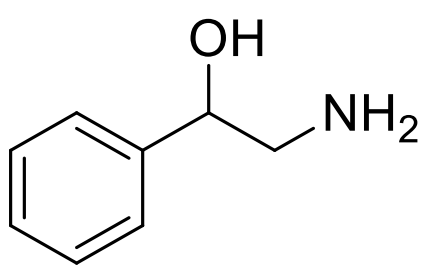

^{1}H NMR ($CDCl_3$, 400 MHz) δ [ppm]: (7.37 (m, 1H, Ar-H_{para}), 7.35 (m, 2H, Ar-H_{meta}), 7.33 (s, 1H, $CDCl_3$), 7.29 (m, 2H, Ar-H_{ortho}), 4.81 (dd, *J* = 3.67 Hz, 1H, OH-CH-C), 2.90 (dd, *J* = 4.03 Hz, 1H, N-CH_2-C), 2.79 (dd, *J* = 7.70 Hz, 1H, N-CH_2-C).

^{13}C HMR ($CDCl_3$, 100 MHz) δ [ppm]: 142.7 (s), 128.3 (s), 127.4 (s), 125.8 (s), 77.0 (t, *J* = 32.28 Hz, $CDCl_3$), 74.2 (s), 49.2 (s).

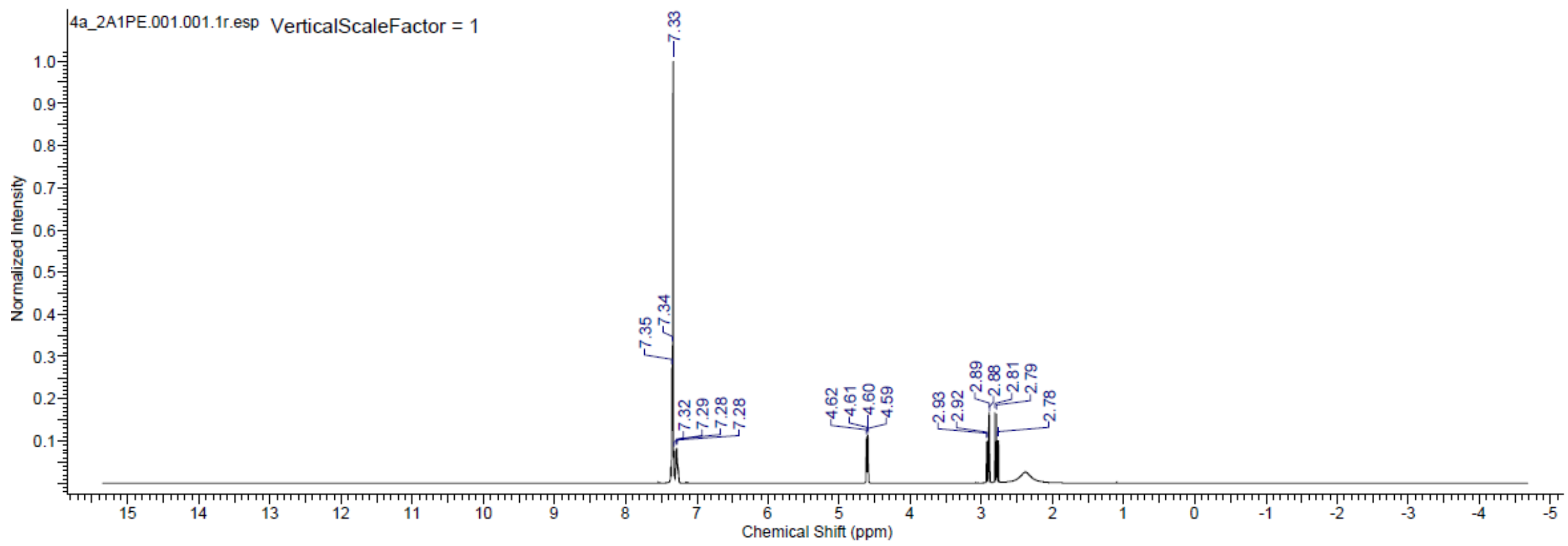

Figure S33. ^{1}H-NMR spectrum of **4a**.

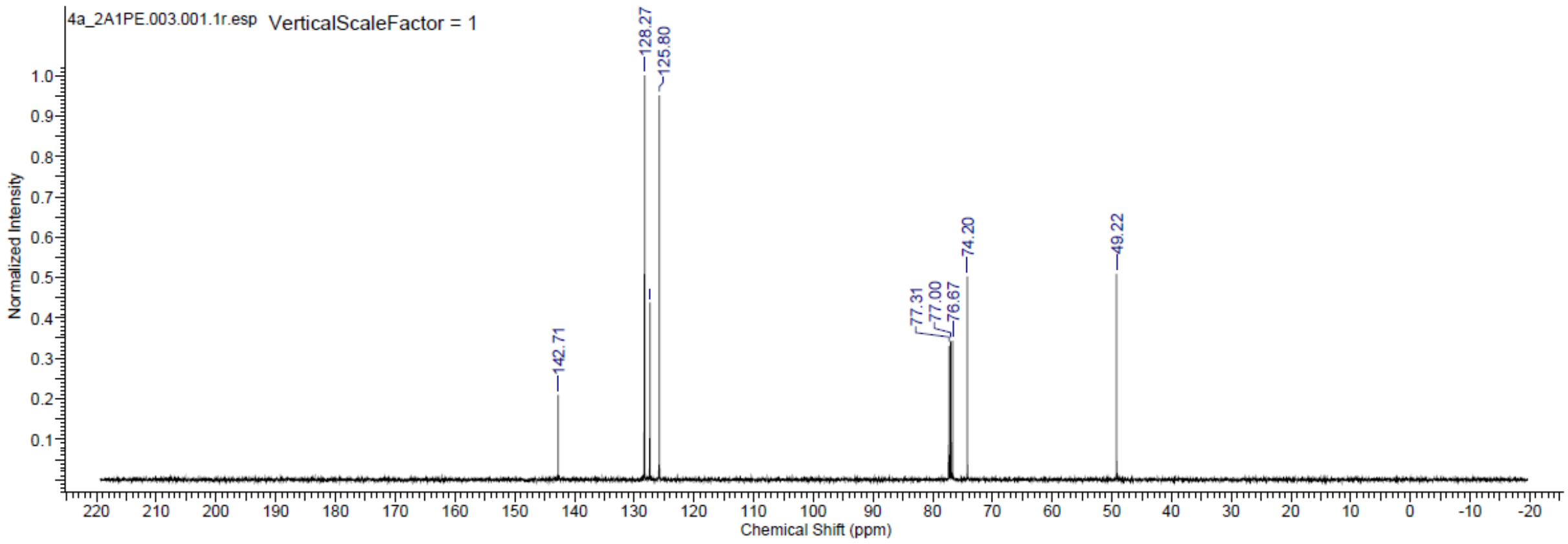

Figure S34. ^{13}C-NMR spectrum of **4a**.

<u>2-amino-1-(3-chlorophenyl)ethanol (**4e**)</u>

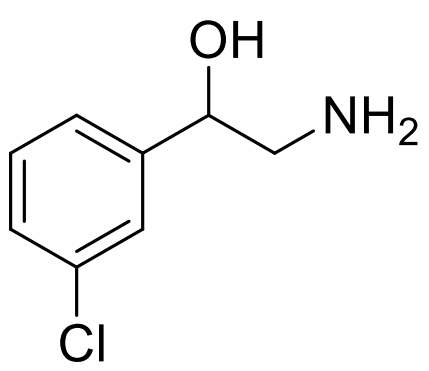

^{1}H NMR ($CDCl_3$, 400 MHz) δ [ppm]: 7.37 (s, 1H, Cl-C-CH_{ortho}), 7.29 (m, 1H, 1H, Ar-H_{para}), 7.27 (s, 1H, $CDCl_3$), 7.26 (m, 1H, Ar-H_{meta}), 7.23 (m, 1H, Ar-H_{ortho}), 4.61 (dd, *J* = 3.91 Hz, 1H, OH-CH-C), 2.97 (dd, *J* = 3.91 Hz, 1H, N-CH_2-C), 2.78 (dd, *J* = 7.83 Hz, 1H, N-CH_2-C).

^{13}C HMR ($CDCl_3$, 100 MHz) δ [ppm]: 144.8 (s), 134.3 (s), 129.6 (s), 127.6 (s), 126.0 (s), 124.0 (s), 77.0 (t, *J* = 32.28 Hz, $CDCl_3$), 73.4 (s), 49.0 (s).

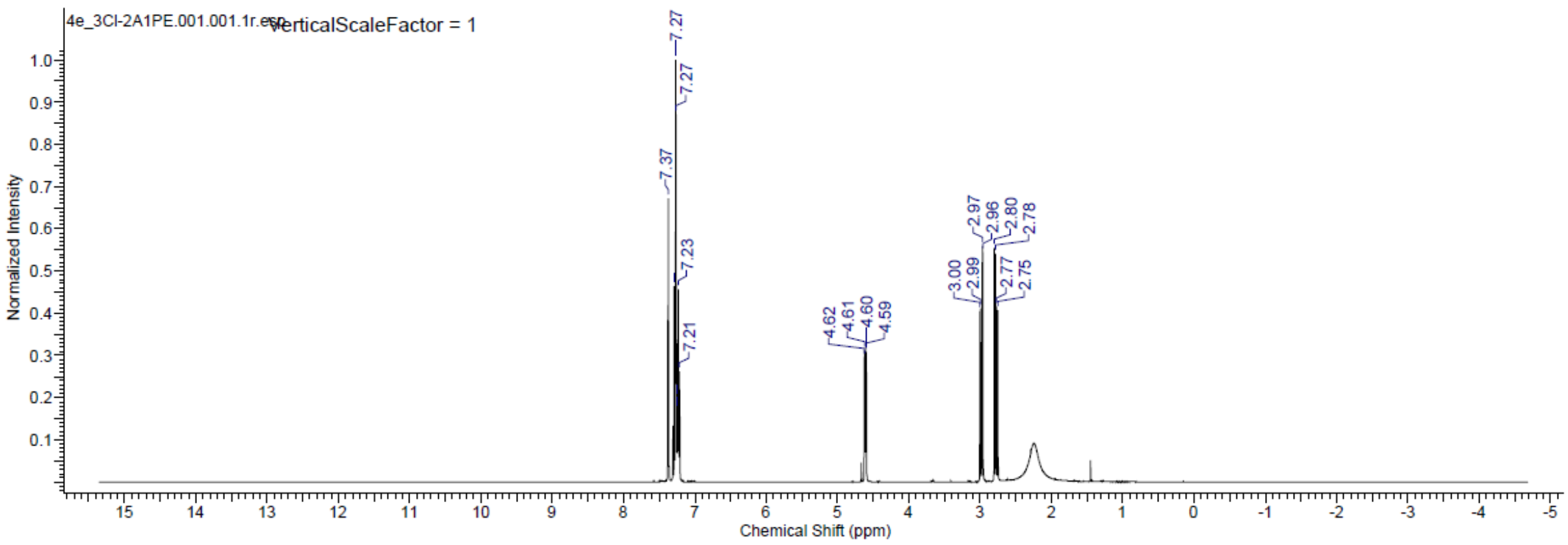

Figure S35. ^{1}H-NMR spectrum of **4e**.

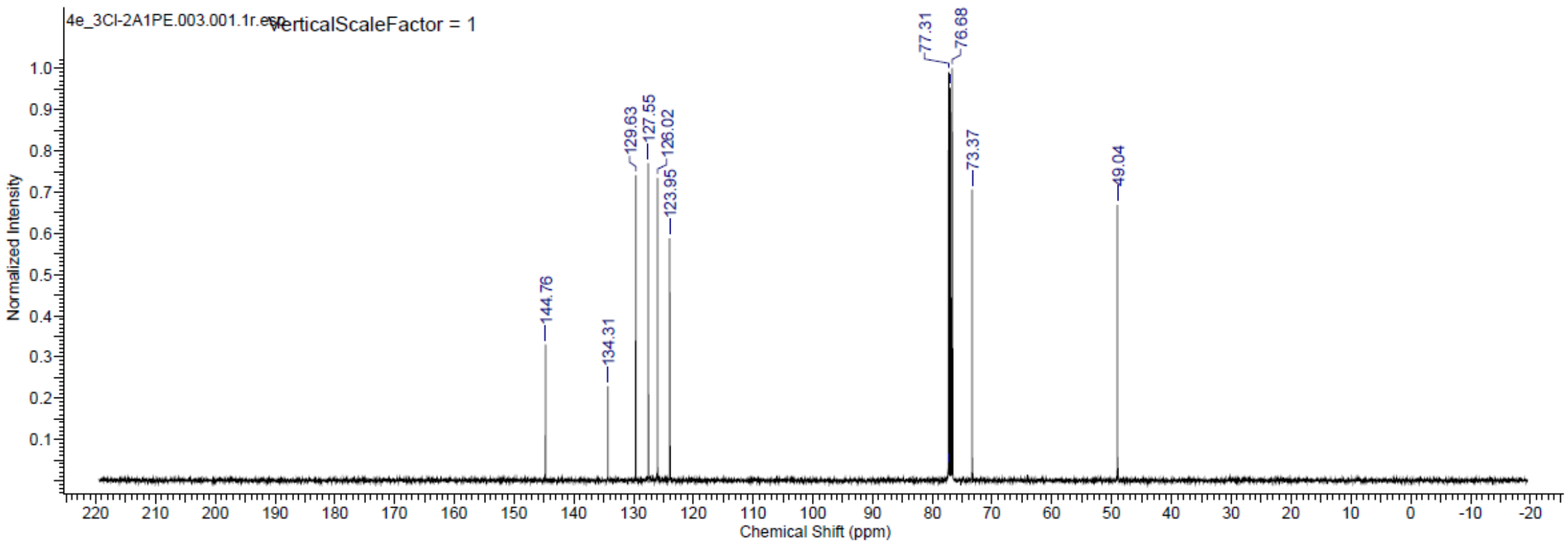

Figure S36. ^{13}C-NMR spectrum of **4e**.

<u>2-amino-1-(3,4-dichlorophenyl)ethanol (**4f**)</u>

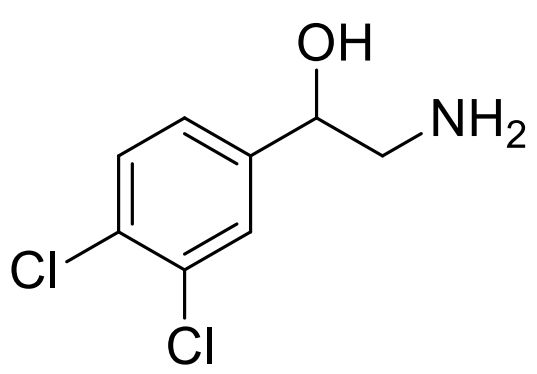

^{1}H NMR ($CDCl_3$, 400 MHz) δ [ppm]: 7.45 (d, *J* = 1.96 Hz, 1H, Cl-C-CH_{meta}), 7.41 (s, 1H, Cl-C-CH_{ortho}), 7.38 (s, 1H, $CDCl_3$), 7.16 (m, 1H, Ar-H_{ortho}), 4.57 (dd, *J* = 3.91 Hz, 1H, OH-CH-C), 2.97 (dd, *J* = 8.93 Hz, 1H, N-CH_2-C), 2.72 (dd, *J* = 7.82 Hz, 1H, N-CH_2-C).

^{13}C HMR ($CDCl_3$, 100 MHz) δ [ppm]: 142.9 (s), 132.5 (s), 131.2 (s), 130.3 (s), 127.9 (s), 125.2 (s),77.0 (t, *J* = 32.28 Hz, $CDCl_3$), 72.7 (s), 48.9 (s).

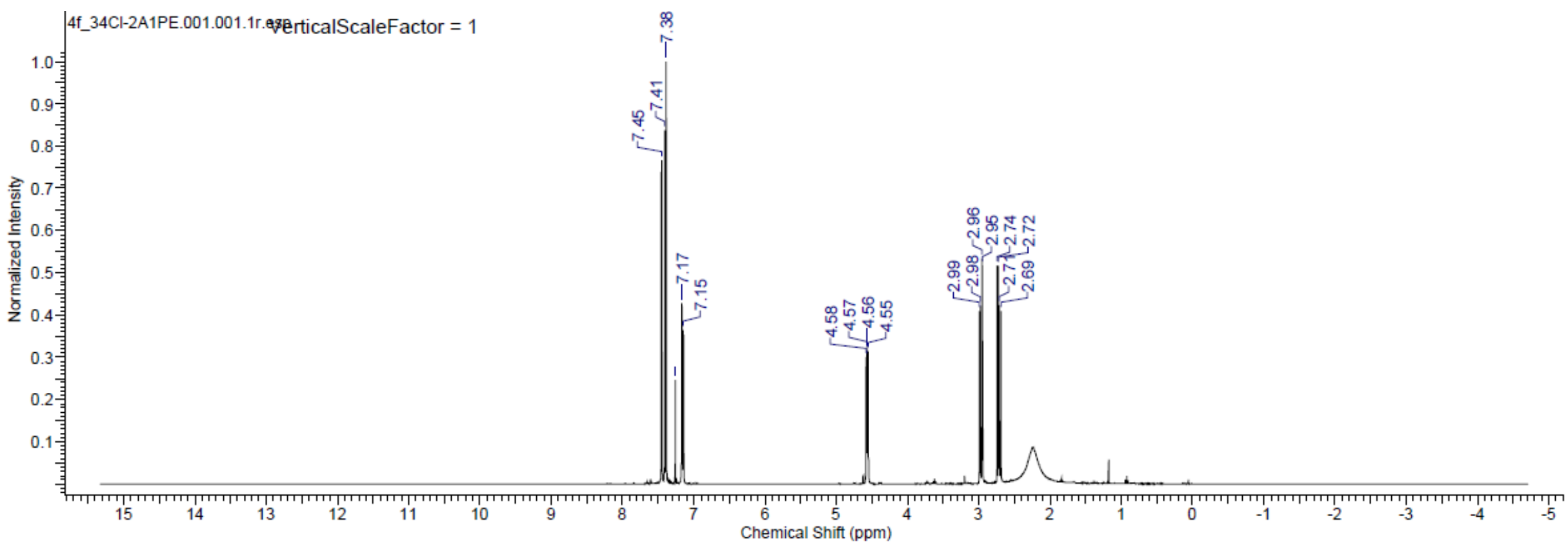

Figure S37. ^{1}H-NMR spectrum of **4f**.

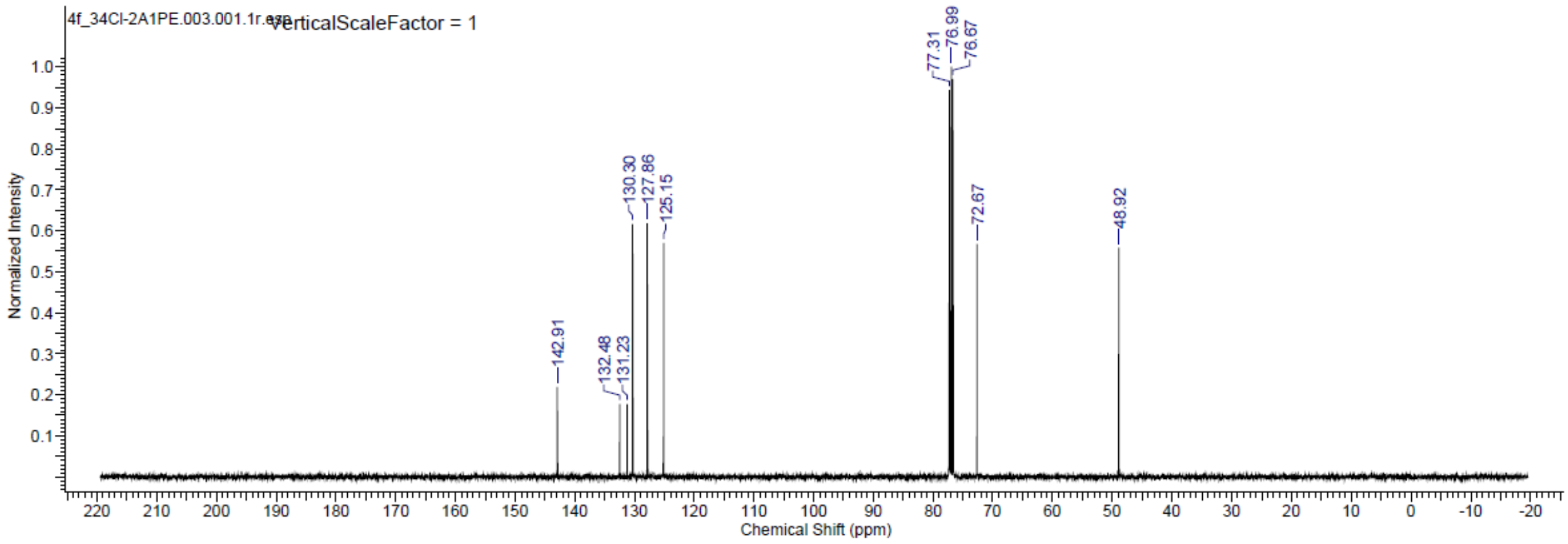

Figure S38. ^{13}C-NMR spectrum of **4f**.

2-(1*H*-imidazol-1-yl)-1-phenylethanol (**5a**)

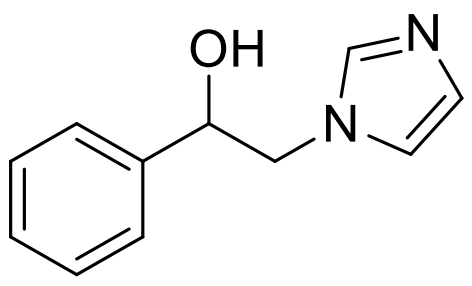

^{1}H NMR ($CDCl_3$, 600 MHz) δ [ppm]: 7.51 (s, 1H, N-CH=N), 7.43 (m, 1H, Ar-H_{para}), 7.35 (m, 2H, Ar-H_{meta}), 7.32 (s, 1H, $CDCl_3$), 7.30 (m, 2H, Ar-H_{ortho}), 7.26 (d, *J* = 4.90 Hz, 1H-N-CH=C), 6.87 (d, *J* = 12.72 Hz, 1H, -C=CH-N=), 4.90 (dd, *J* = 3.45 Hz, 1H, OH-CH-C), 4.08 (dd, *J* = 4.72 Hz, 1H, N-CH_2-C), 3.54 (dd, *J* = 2.54 Hz, 1H, N-CH_2-C), 3.25 (s, 1H, OH).

^{13}C HMR ($CDCl_3$, 150 MHz) δ [ppm]: 141.1 (s), 137.6 (s), 130.0 (s), 128.7 (s), 128.6 (s), 128.2 (s), 125.8 (s), 119.7 (s), 118.8 (s), 77.0 (t, *J* = 31.61 Hz, $CDCl_3$), 73.32 (s), 54.72 (s).

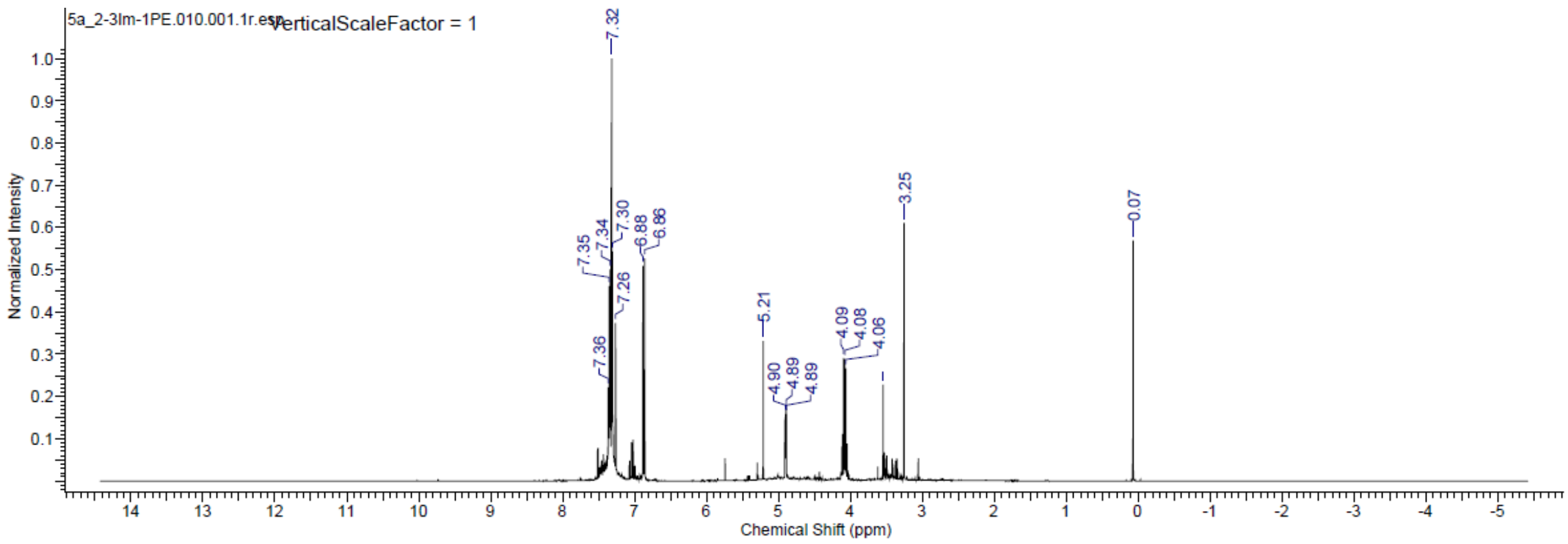

Figure S39. ^{1}H-NMR spectrum of **5a**.

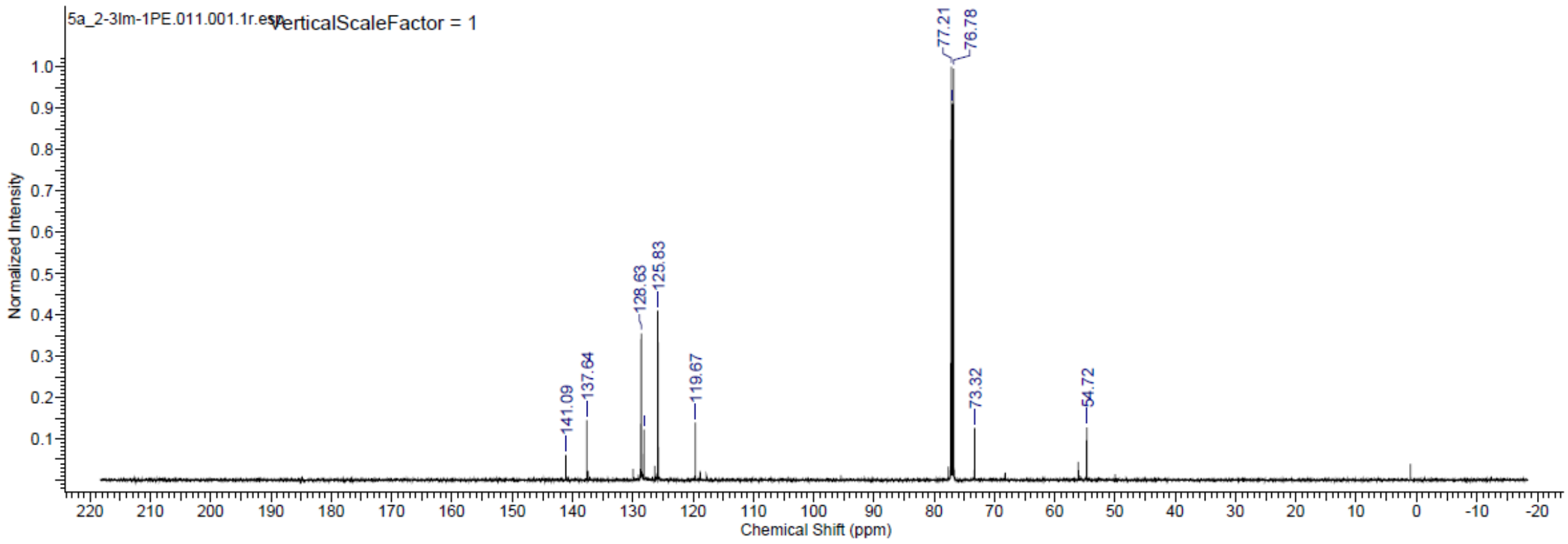

Figure S40. ^{13}C-NMR spectrum of **5a**.

<u>2-((2-(benzyloxy)benzyl)amino)-1-(3-chlorophenyl)ethanol (**5e**)</u>

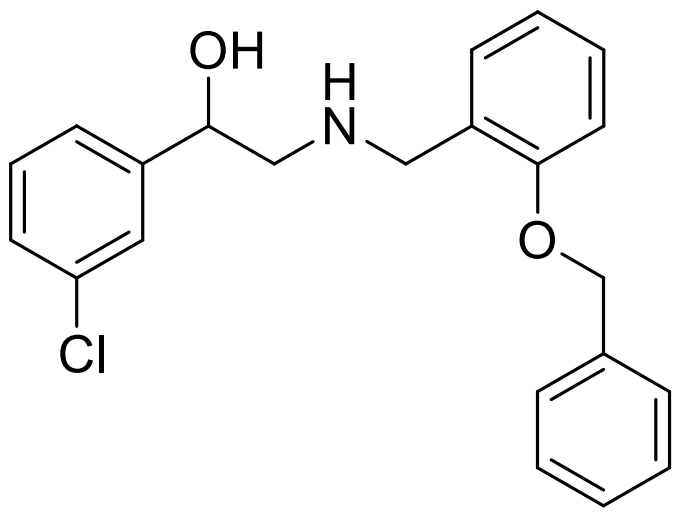

^{1}H NMR ($CDCl_3$, 600 MHz) δ [ppm]: 7.33 (m, 1H, Ar-H), 7.32 (m, 1H, Ar-H), 7.31 (m, 1H, Ar-H), 7.30 (m, 1H, Ar-H), 7.28 (s, 1H, $CDCl_3$), 7.25 (m, 1H, Ar-H), 7.22 (m, 1H, Ar-H), 7.16 (m, 1H, Ar-H), 7.11 (m, 1H, Ar-H), 7.06 (m, 1H, Ar-H), 6.88 (m, 1H, Ar-H), 6.87 (m, 1H, Ar-H), 6.85 (m, 1H, Ar-H), 6.83 (m, 1H, Ar-H), 5.02 (s, 2H, -O-CH_2-Ar), 4.97 (t, J = 5.45 Hz, 1H, OH-CH-C), 4.63 (s, 2H, N-CH_2-Ar), 3.75 (s, 1H, OH), 2.71 (dd, J = 3.63 Hz, 1H, N-CH_2-C), 2,52 (dd, J = 9.08 Hz, 1H, N-CH_2-C), 2.07 (s, 1H, NH).

^{13}C HMR ($CDCl_3$, 150 MHz) δ [ppm]: 155.75 (s), 143,8 (s), 135.8 (s), 133.2 (s), 129.0 (s), 128.5 (s), 127.8 (s), 127.7 (s), 127.6 (s), 127.5 (s), 127.1 (s), 126.4 (s), 126.2 (s), 124.9 (s), 122.9 (s), 120.0 (s), 119.8 (s), 110.7 (s), 76.0 (t, J = 31.61 Hz, $CDCl_3$), 69.9 (s), 69.0 (s), 54.9 (s), 48.0 (s).

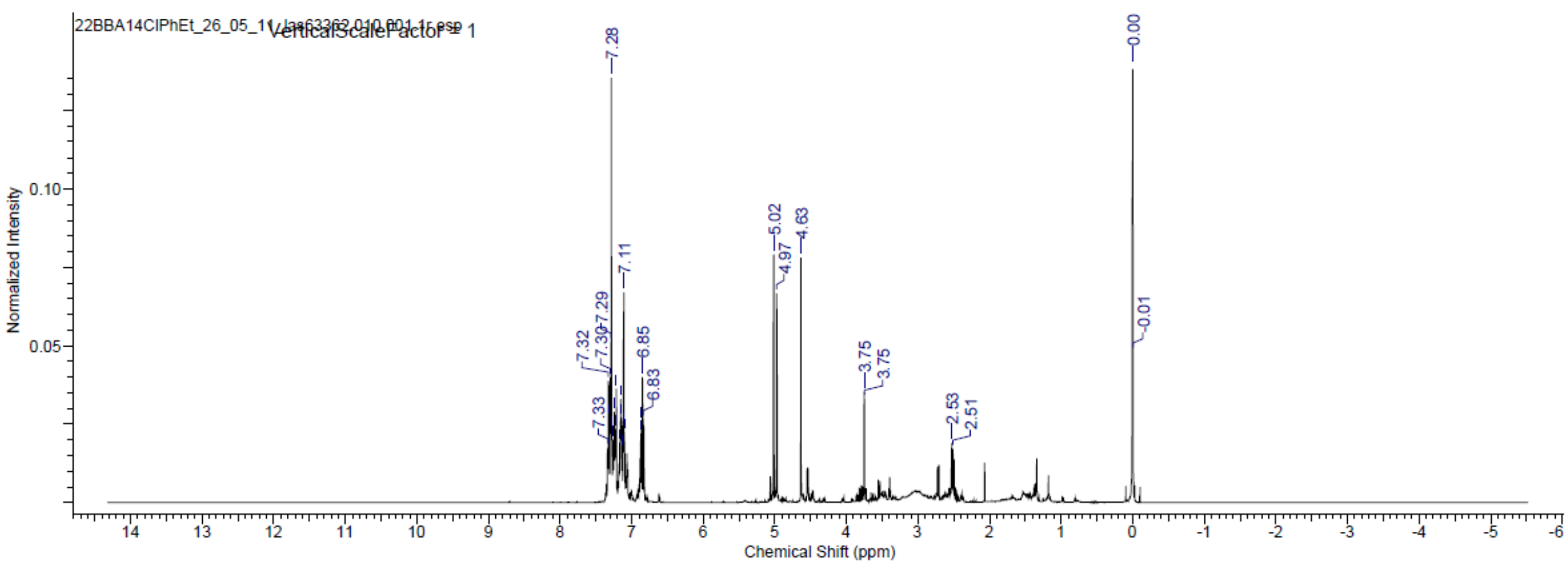

Figure S41. ^{1}H-NMR spectrum of **5e**.

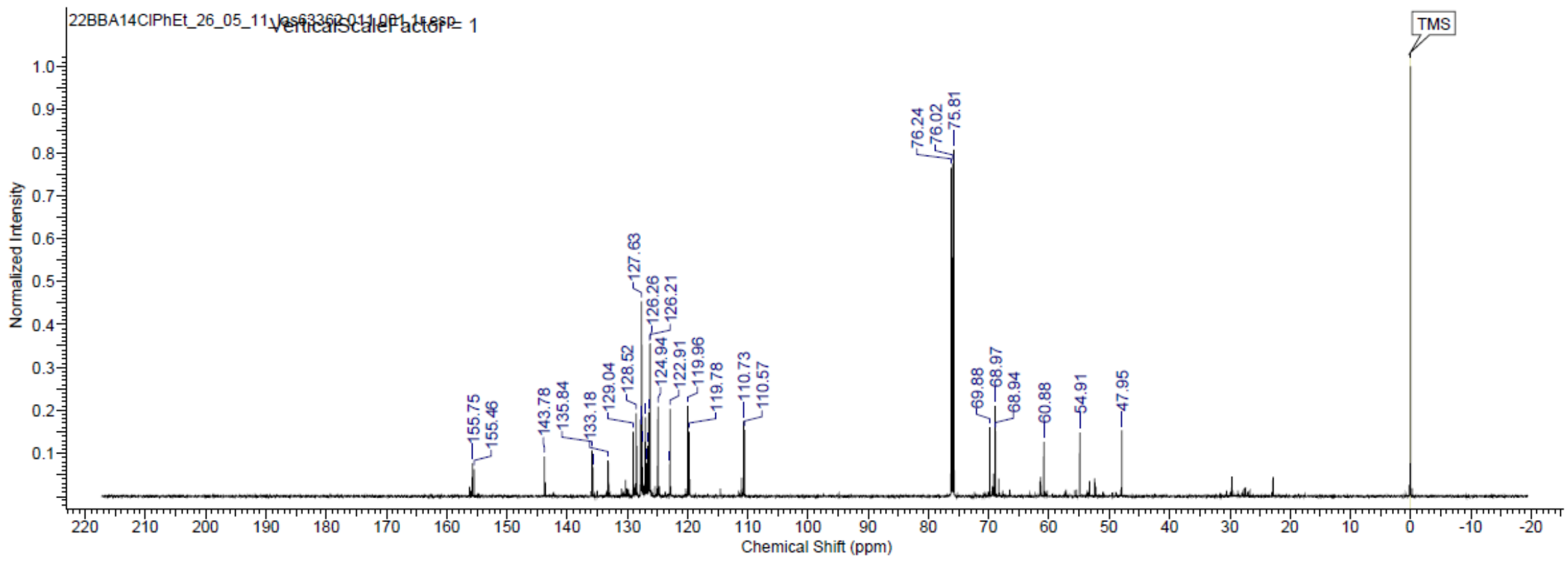

Figure S42. ^{13}C-NMR spectrum of **5e**.

1-(3,4-dichlorophenyl)-2-((naphthalen-2-ylmethyl)amino)ethanol (**5f**)

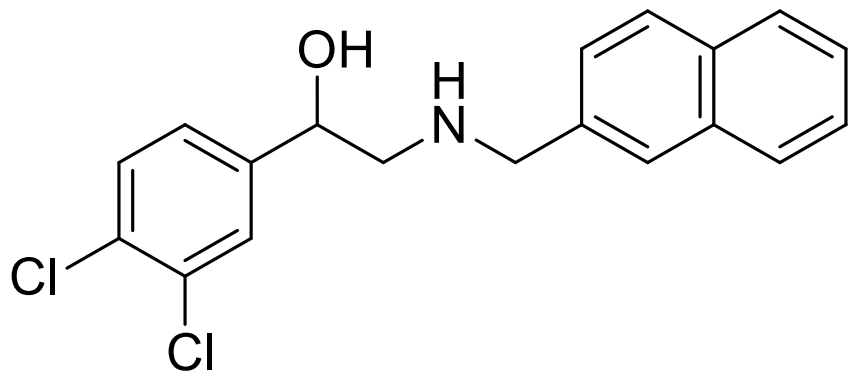

^{1}H NMR ($CDCl_3$, 400 MHz) δ [ppm]: 8.00 (m, 1H, Ar-H), 7.98 (m, 1H, Ar-H), 7.87 (m, 1H, Ar-H), 7.77 (m, 1H, Ar-H), 7.73 (m, 1H, Ar-H), 7.65 (m, 1H, Ar-H), 7.42 (m, 1H, Ar-H), 7.36 (m, 1H, Ar-H), 7.32 (m, 1H, Ar-H), 7.19 (s, 1H, $CDCl_3$), 7.11 (m, 1H, Ar-H), 4.61 (dd, J = 5.26 Hz, 1H, OH-CH-C), 3.93 (s, 2H, N-CH_2-Ar), 2.90 (dd, J = 3.55 Hz, 1H, N-CH_2-C), 2.64 (dd, J = 8.80 Hz, 1H, N-CH_2-C).

^{13}C HMR ($CDCl_3$, 100 MHz) δ [ppm]: 142.7 (s), 137.0 (s), 133.4 (s), 132.8 (s), 132.5 (s), 131.5 (s), 131.3 (s), 130.3 (s), 128.4 (s), 127.9 (s), 127.7 (s), 126.6 (s), 126.3 (s), 126.2 (s), 125.8 (s), 125.1 (s), 77.0 (t, J = 32.28 Hz, $CDCl_3$), 70.5 (s), 56.1 (s), 53.6 (s).

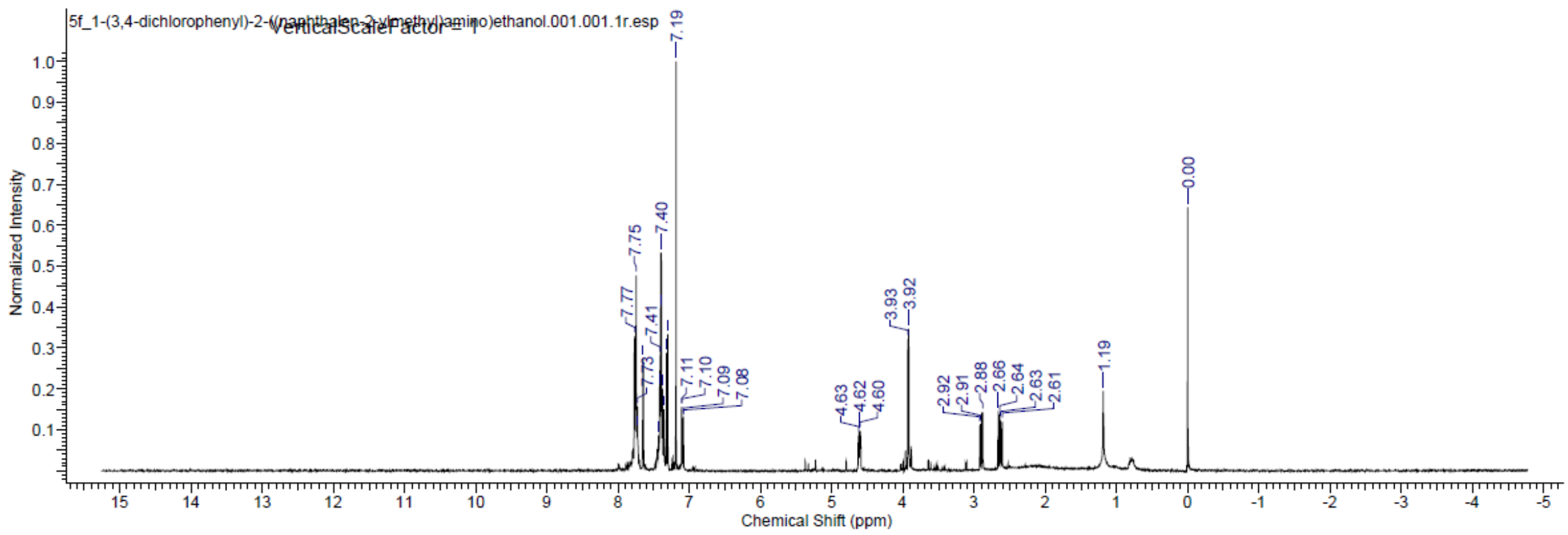

Figure S43. ^{1}H-NMR spectrum of **5f**.

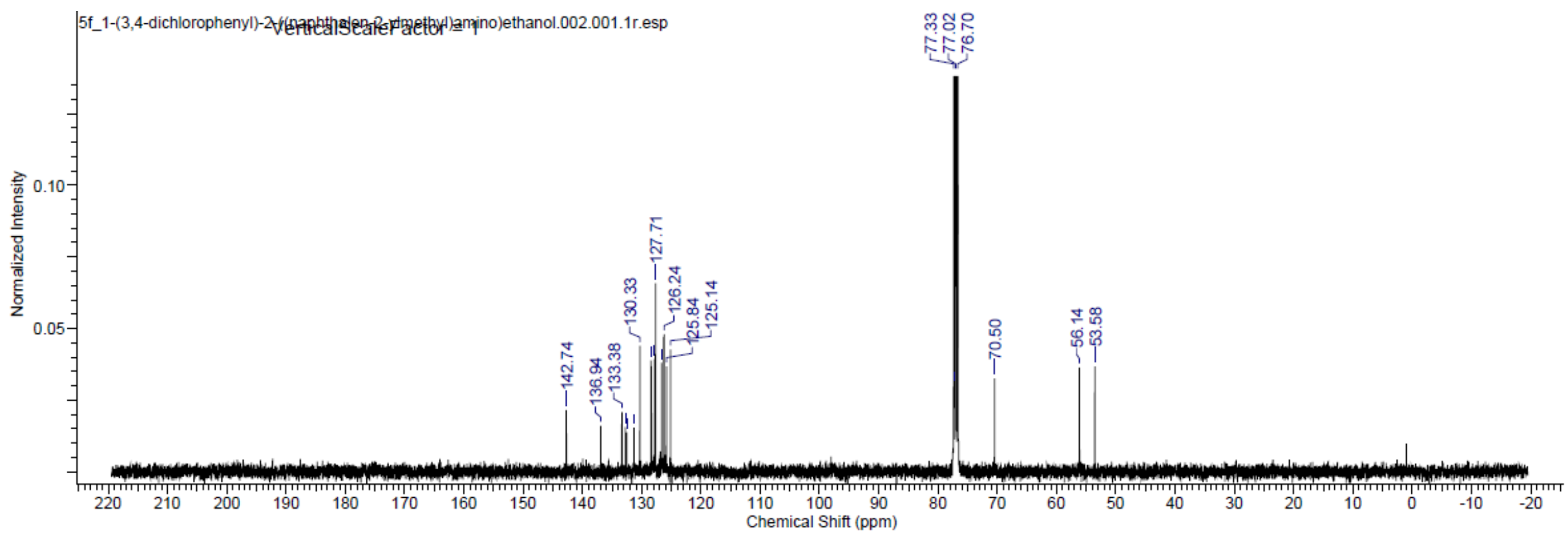

Figure S44. ^{13}C-NMR spectrum of **5f**.

3. Preparation of biocatalyst

Chemical competent cells of *S. cerevisiae* (INVSc1) were prepared for the transformation using transformation buffer (15% (v/v) glycerol, 500 µM EDTA, 100 mM lithium acetate, 5 mM Tris-HCl pH 7.4). For each transformation, 100 ng of prepared plasmid pAGT572_Nemo2.0_*Mth*UPO or pAGT572_Nemo2.0_TteUPO, respectively, were mixed with 10 µl of pure single-stranded salmon sperm DNA (Merck, D7656-1ML) and added to a 200 µl stock of competent INVSc1 cells on ice. 600 µl of the beforehand prepared transformation buffer (100 mM lithium acetate, 10 mM Tris-HCl pH 7.4, 40% (v/v) polyethylene glycol 4000, 1 mM EDTA) were added and the cells were left to incubate under shaking (850 rpm, 30 °C) for 30 minutes. Before the heat shock at 42 °C for 15 minutes, 70 µl of DMSO were added to the mixture. After pelleting the cells via short centrifugation, the supernatant was discarded. The cell pellet was resuspended in 350 µl of sterile ultrapure water. 100 µl of the resulting solution were plated on a Synthetic Complement (SC) Drop Out plate, containing a total of 2% (w/v) glucose as carbon source, but lacking Uracil as selection marker. After incubating the plate for at least 48 hours at 30 °C, single colonies appeared, from which one each was used to inoculate 100 ml SC Drop medium with raffinose as carbon source. To the sterile baffled flasks, in which the pre-culture was left to shake for two days (130 rpm, 30 °C), 25 µl chloramphenicol (50 mg/ml in ethanol) were added to ensure a clean growth of yeast cells without unwanted bacteria. After incubation, the whole pre-culture was added to the main culture in a 1-liter-baffled flask; 333 ml 1,5x YP (3% (w/v) peptone, 1.5% (w/v) yeast extract), 50 ml D-galactose (20% (w/v)), 16.5 ml pure ethanol, 36 ml KPi buffer (1 M, pH 6.0), 16 ml $MgSO_4$ (100 mM), 48 ml sterile ultrapure water and 0.25 ml chloramphenicol stock solution (50 mg/ml). The main culture was then incubated for another four days (120 rpm, 25 °C). Afterwards, the cell solution was centrifuged (3500 rpm, 4 °C, 30 min) and the cells were discarded. The solution went through ultrafiltration using a Vivaflow 200 filtration module (30 kDa, PES, Sartorius), in order to increase the concentration of the enzyme. The obtained dark-brown supernatant was sterile filtered and stored at 4 °C.

Once enzyme production had been established and optimised, these experiments were no longer carried out in shaking flasks, but in a 5-litre fermenter equipped with a stirrer and pH control. The compounds used were scaled up by a factor of 2, and for each run six pre-cultures were added to the

main culture medium. This increased the cell yield and reduced the total duration of the process by up to two days. The resulting enzyme solution was then subjected to ultrafiltration and concentrated in the same way.

4. Enzymatic conversion

The conversion of substrates **2a–m** to products **3a–m** was tested using *Mth*UPO and *Tte*UPO. The tests were initially carried out on a 1 ml scale. The substrates were used at concentrations of 5, 10 and 20 mM, and it was found that a significant reduction in activity occurred at concentrations of 20 mM and above. Consequently, 10 mM was considered the optimal concentration above which the substrates exert an inhibitory effect. The substrates were used as 100 mM stock solutions in acetonitrile and added to the aqueous reaction solutions in appropriate quantities. The acetonitrile also served to ensure the necessary solubility. To achieve this, a constant low supply of hydrogen peroxide had to be established. To this end, L-amino acid oxidase (LAAO, 150 µl, 21 mg/ml, prediluted 1:1000) was used, along with L-alanine (20 mM) as substrate for this second enzyme. The concentration of the amino acid was deliberately set at twice the substrate concentration, as previous studies had shown this to be optimal. The reaction was carried out at pH 7, which was adjusted using KPi buffer (100 µl, 1 M, pH = 7). The concentrated UPO solutions (350 µl, 1.44 U/ml) were added and the remaining volume was filled with ultrapure water. The reaction began with the addition of LAAO, which generated hydrogen peroxide for the UPO, and was terminated after six hours in the initial tests, and after twenty-four hours for the most promising substrates. For all subsequent experiments, particularly those on a larger scale, the completion of the reaction was monitored using gas chromatography and then terminated accordingly. On a larger scale, up to 10 ml total volume, the ratios of the stock solutions used were maintained. If necessary, additional alanine solution was added to continue the production of hydrogen peroxide. After a duration of twenty-four hours, in every case, complete conversion had been reached. The products were extracted using dichloromethane and collected for further experiments and analysis.

5. Performed synthesis

Applying the Boc protecting group

The amines (**1a–m**) were each dissolved in 10 ml of dichloromethane. For each protection reaction, 5 mM of the respective amine had been used. To promote the reaction and improve the yields, 5 mol% of triethylamine was added to each solution. Boc_2O was then added in a 1.1-fold excess (5.5 mM, 1.177 ml) and the resulting solution was stirred at room temperature overnight. The formation of gas bubbles (CO_2) was observed, indicating the progress of the reaction. After the reaction was complete, the solvent and any remaining Boc_2O were removed using a rotary evaporator; the resulting solid was dissolved in dichloromethane and washed with water. The phases were separated, and the DCM phase was evaporated again using the rotary evaporator. The solids obtained are the protected amines. The yields were quantitative.

Removal of the Boc protecting group

The Boc protecting group prevents a variety of chemicals from reacting with the amine it is applied to. However, the Boc group is unstable in acidic conditions. In order to remove it from the products **3a-m** and form the free amino alcohols **4a-m**, an aqueous solution of the protected compounds can be treated with a small volume of concentrated hydrochloric acid, lowering the pH value to at least 2. After shaking at 800 rpm at 60 °C, the free amino alcohols could be extracted using dichloromethane, which was just evaporated afterwards. The amino alcohols obtained were then analysed for their enantiomeric composition and could be used as pharmaceuticals in this form. All yields were quantitative, and the selectivity is not considered to be affected by the de-protection.

2-(1*H*-imidazol-1-yl)-1-phenylethanol (**5a**)

Compound **5a** was synthesised in a sealed Teflon vessel, which was additionally clamped inside a steel autoclave to prevent gaseous components from escaping during the reaction. The size of the batch was determined by the limited capacity of the vessel used. Nevertheless, following completion of the enzymatic conversion of **2a** to **3a** and the subsequent removal of the Boc protecting group by the addition of concentrated hydrochloric acid, **4a** was obtained as the starting material for this reaction.

The synthesis was carried out in a mixture of water and methanol. In 5 ml of methanol, 2-amino-1-phenylethanol (**4a**, 0.6859 g, 5 mmol), glyoxal (0.509 ml, 10 mmol) and ammonium chloride (0.5349 g, 10 mmol) were added to 5 ml of methanol and mixed with 37% aqueous formaldehyde solution (0.9 ml). This mixture was carefully homogenised before the vessel was sealed and placed in the steel autoclave. The reaction was carried out in an oven at 80 °C overnight. An amber-colored solution and a caramel-colored solid with a sweetish aroma were obtained. Both were dried in a rotary evaporator, then dissolved in NaOH solution (2 M) and washed several times with dichloromethane. After evaporation of the organic phase, a yield of 0.913 g (97%) was obtained.

2-((2-(benzyloxy)benzyl)amino)-1-(3-chlorophenyl)ethanol (**5e**).

The synthesis of **5e** involved the reductive alkylation of an amine. For this reaction, **4e** (250 mg, 1.46 mmol) and 2-(benzyloxy)benzaldehyde (340 mg, 1.6 mmol) were dissolved in 10 ml of dichloromethane/tetrahydrofuran (1:1) and stirred together with diisopropylethylamine (DIPEA, 508.6 µl, 2.9 mmol) for two hours at room temperature. Sodium cyanoborohydride (128,4 mg, 2.04 mmol) was then added to reduce the presumed imine. The mixture was left to stir overnight, then evaporated and redissolved in dichloromethane, followed by washing with water. The organic phase was separated and washed with brine. After drying with $MgSO_4$, the solution was filtered and concentrated in a rotary evaporator. Hexane was then added to this concentrated solution until a pale-yellow solid precipitated. This was collected, washed with hexane and subsequently dried in a rotary evaporator. A yield of 423 mg (81%) was obtained.

1-(3,4-dichlorophenyl)-2-((naphthalen-2-ylmethyl)amino)ethanol (**5f**)

The synthesis of **5f** was carried out in a similar manner. The amino alcohol **4f** (250 mg, 1.2 mmol) and 2-naphthaldehyde (0.135 ml, 1.34 mmol) were dissolved in dichloromethane (10 ml) with diisopropylethylamine (DIPEA, 0.413 ml, 2.45 mmol). After stirring for two hours at room temperature, sodium triacetoxyborohydride (360 mg, 1.698 mmol) was added in this case and the reaction mixture was left to stir overnight. The solvent was then evaporated, and the remaining solid was dissolved in dichloromethane and washed with water. The organic phase was isolated and washed with salt water, then dried over $MgSO_4$ and filtered. After concentration in a rotary evaporator, hexane was added until a pale yellow solid precipitated. This was washed with hexane and then dried. The yield was 357 mg (85%).

6. PHOTOMETRIC MEASUREMENTS

A Spark Multimode Microplate Reader (Tecan Trading AG, Switzerland) was used to perform every photometric measurement. For absorbance measurements, a xenon flash lamp (spectral range from 200 to 1000 nm, optical density range from 0 to 4 OD, scan speed ≤ 5 sec, wavelength accuracy < 0.3 nm) was used. The experiments were performed in Microplates (96 well, PS, F-Bottom, clear) purchased from Greiner Bio-One.

NBD Assay

Using 5-nitro-1,3-benzodioxole (NBD), the activity of the unspecific peroxygenase was measured. The enzyme converted the substrate to 4-Nitrocatechol, which can be measured at a wavelength of 425 nm. The reactions were performed in the above mentioned well plates in a total volume of 200 µl per well, containing 0.5 mM NBD (stock solution of 10 mM, dissolved in acetonitrile). 10 µl of concentrated UPO solution (1.44 U/ml equals 0.072 U/ml in the final assay volume) and 10 µl of pre-diluted *hc*LAAO4 or H_2O_2 solution, respectively, as well as 100 mM HEPES buffer (pH 7.0) were contained in every assay reaction. In case the LAAO was used, the same concentration of L-alanine was added to the solution in order to supply the oxidase for the production of H_2O_2. Every well was filled up with ultrapure water in order to match a total volume of 200 µl. The measurements were performed over the duration of five minutes, giving the slope of each measurement, which was then used to determine the activity of the UPO under the given circumstances. All reactions were performed in triplicates.

H_2O_2 Assay

In order to determine the right conditions for the UPO, it was necessary to measure the amount of H_2O_2 in a given reaction medium as well as the activity of the LAAO, which was used to *in situ* generate H_2O_2 to supply the UPO.

First, there was a molybdate assay, utilising potassium hydrogen phthalate and ammonium molybdate to determine the concentration of H_2O_2 of a solution, allowing the observation of the production of H_2O_2 using LAAO and thus giving insights about the activity of the enzyme. Measurements took place at a wavelength of 351 nm with a total volume of 200 µl per well. 60 µl potassium hydrogen phthalate solution (0.5 M) and 60 µl colour solution (0.05 M NaOH, 0.4 M KI, 10-4 M ammonium molybdate) were mixed. Adding 80 µl of H_2O_2 containing sample solution (10 – 100 mM) started the reaction. The calibration took place using precise H_2O_2 standard solutions. The data gathered from these experiments was used to select the correct ratio of UPO and LAAO the oxyfunctionalisation reaction.

Secondly, a POX/o-Dianisidin Assay was used for the determination of the LAAO activity. The total volume of the assay solution per well contained 1.25 µl (0.2 mg/ml) o-Dianisidin (stock solution of 32 mg/ml, solved in DMSO), 10 µl L-alanine (100 mM in ultrapure water), 1.25 µl peroxidase solution (POX, 400 U/ml) and 10 µl sample and was filled up with TEA buffer (50 mM, pH 7.0). Measurements took place at a wavelength of 436 mM for twenty minutes at 30 °C and allowed statements about the activity of the LAAO.

7. GAS CHROMATOGRAPHY

A Nexis GC – 2030 system from Shimadzu Europa GmbH (Duisburg, Germany) was used for all gas chromatography measurements. The device is equipped with an AOC-20i Plus Auto Injector and a SH-5 column (length 30 m, ID 0.25, DF 0.25, temperature range -60 to 330/350 °C, P/N 221-75701-30). The stationary phase of the column is composed of 5% diphenyl polysiloxane and 95% dimethyl polysiloxane. Hydrogen was used as the main career gas with a flow rate of 36.0 ml/min and compressed air (200.0 ml/min) for the flame ionization detector (FID). For technical reasons, nitrogen was not used as an additional make-up gas. Samples were converted in aqueous solution and extracted with dichloromethane. The temperature program for samples with low boiling points was driven from 100 to 200 °C with a heating rate of 10 °C/min, followed by a holding step over two minutes and a second heating step up to 250 °C with a heating rate of 25 °C/min over a total time frame of sixteen minutes. For higher boiling compounds, such as the ones containing the Boc protecting group, a different temperature program was used. Starting at 100 °C, the temperature was raised with a rate of 10 °C/min up to 300 °C, where in was hold for 2 minutes before raising it again to 310°C with a rate of 5 °C/min.

The following section presents examples of the measurement curves for the enzymatic conversion of Boc-protected amines (**2a**, **2e**, **2f**) to the corresponding amino alcohols (**3a**, **3e**, **3f**). The reaction took place in an aqueous medium in each case, from which the product was extracted using dichloromethane. n-Decane served as the internal standard. Example measurements were deliberately selected at points in time when the conversion to the amino alcohol had not yet been completed, in order to demonstrate the shift in the signals from the reactant and the product.

Enzymatic conversion of **2a** into **3a**

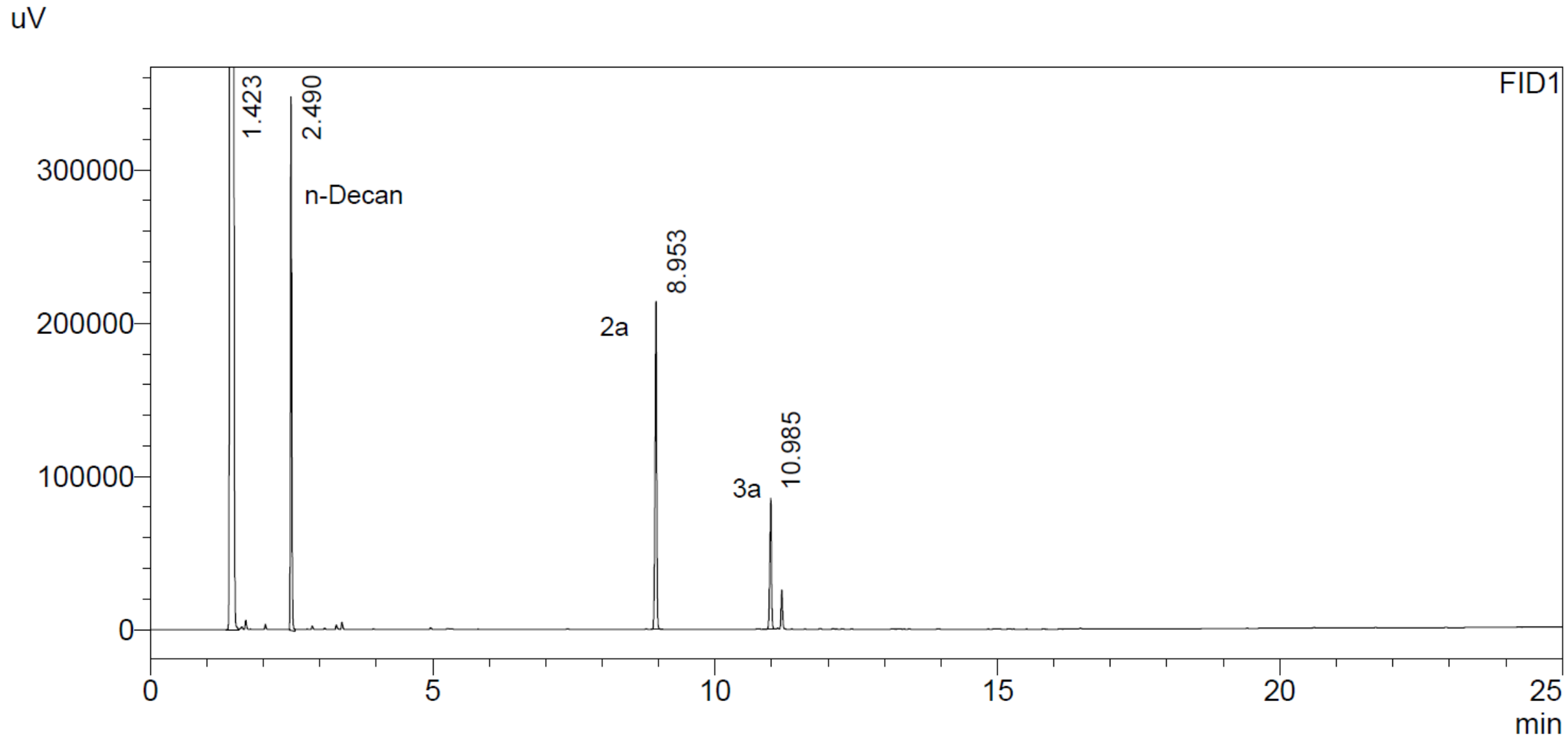

Figure S45. Gas chromatogram of the conversion of **2a** into **3a**.

Enzymatic conversion of **2e** into **3e**

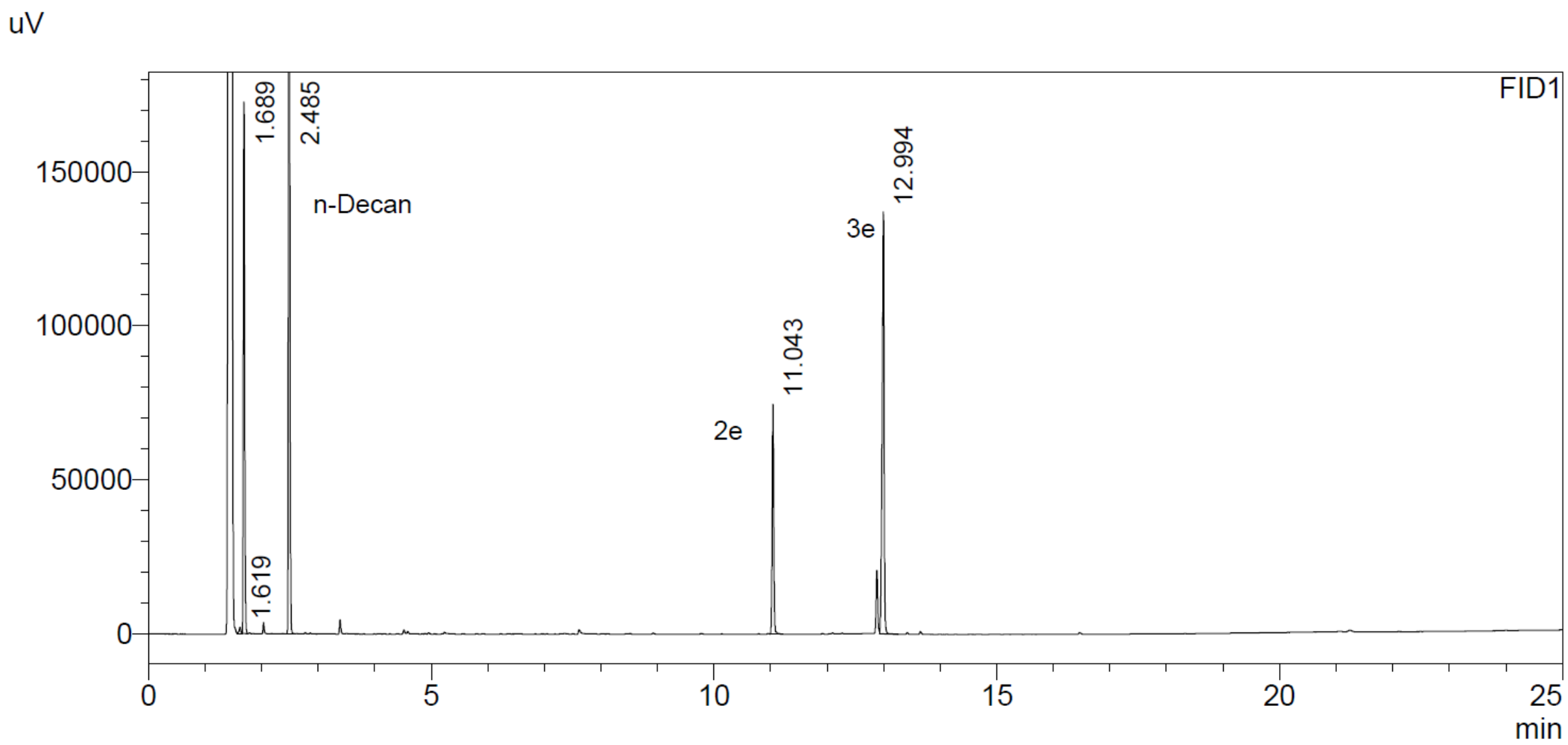

Figure S46. Gas chromatogram of the conversion of **2e** into **3e**.

<u>Enzymatic conversion of **2f** into **3f**</u>

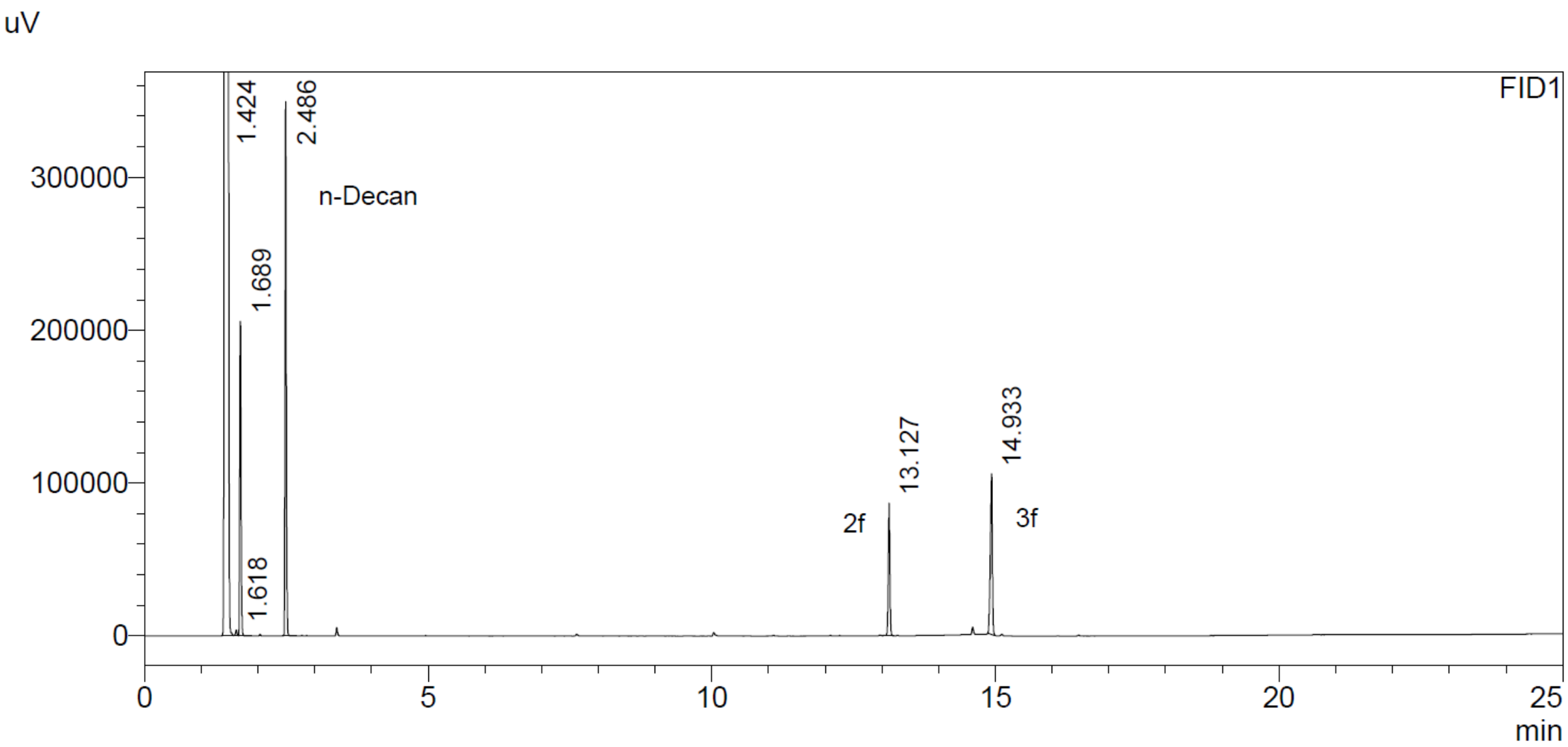

Figure S47. Gas chromatogram of the conversion of **2f** into **3f**.

8. HPLC ANALYTICS AND DETERMINATION OF ENANTIOMERIC EXCESS

To determine the enantiomeric excess (ee), derivatisation was carried out using 2,3,4,6-tetra-O-acetyl-β-D-glucopyranosyl isothiocyanate (GITC). Reaction at the amino group of the unprotected amino alcohols (**4a**, **4e**, **4f**) yields a thiourea derivative, which can be chromatographically separated as diastereomers due to the differing strengths of interaction between the individual isomers and the stationary phase. The separation then took place on a reverse-phase column. The reaction scheme for the derivatization reaction is displayed below.

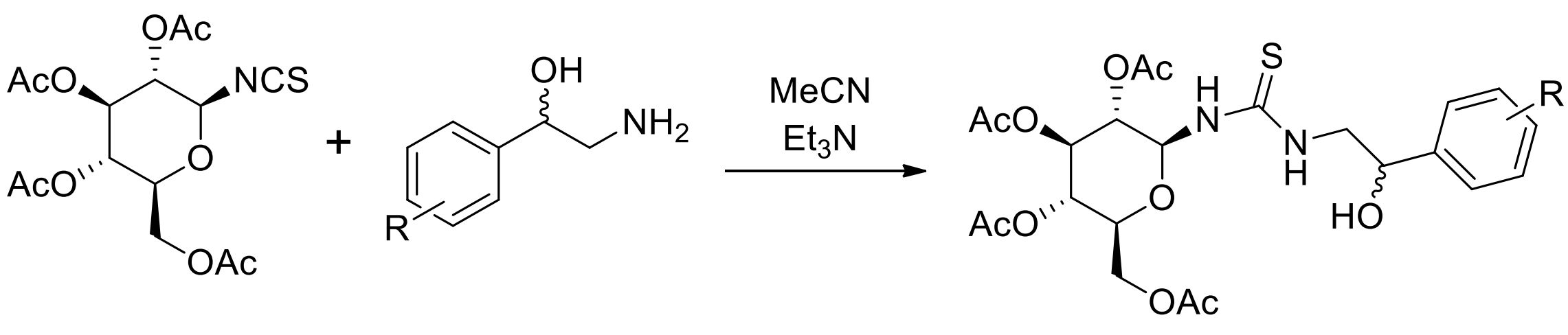

Figure S48. Reaction scheme for the derivatisation on amino alcohols with GITC.

The HPLC system used was a Shimadzu LC-MS (Nexera HPLC) 2050. An InfinityLab Poroshell 120 EC-C18 column from Agilent (3.0 × 150 mm, 2.7 µm), equipped with a Phenomenex AJ0-8775 SecurityGuard ULTRA cartridge, was used. For mass spectrometry, DUIS was used as the ionisation mode, employing both electrospray ionisation (ESI) and atmospheric pressure chemical ionisation (APCI) simultaneously. To this end, a nebulising gas flow rate of 2.0 L/min, a drying gas flow rate of

5.0 L/min and a heating gas flow rate of 7.0 L/min were set, where nitrogen was used in all three cases. The desolvation temperature was 420 °C and the desolvation line temperature was 200 °C.

The oven temperature was set to 40 °C and the flow rate was 400 µl/min in each case. Water (A, LC/MS grade, 0.1% formic acid) and methanol (B, LC/MS grade, 0.1% formic acid) were used as eluents and pumped through the column as a mixture during the analysis. Depending on the compound being studied, two different methods were used. For **4a** and **4e**, the measurements lasted a total of 50 minutes. Initially, an A:B ratio of 59:41 was set and maintained for 14 minutes. Within one minute, the ratio was then changed to a ratio of A:B 38:62 and maintained until the 44th minute, before returning to the A:B ratio of 59:41 within one minute and maintaining this until the end of the measurement. A shorter method was used for **4f**. Starting with an A:B ratio of 59:41, which was maintained for the first 14 minutes, the ratio was then adjusted to 38:62 within one minute and maintained until the 29th minute. Subsequently, the system was adjusted back to the initial A:B ratio of 59:41 within one minute, and this value was maintained for a further five minutes. Each measurement thus lasted 35 minutes. All measurements were carried out in both scan and SIM modes. The voltage for the positive ionisation mode was 3.0 kV. In the negative mode, an interface voltage of -5.0 kV was set. The scan was carried out in the range from m/z 300 to 800 for both polarities. In SIM mode, ions with m/z 527, 549, 561 and 595 were detected.

The chromatograms shown below display the measurements of the amino alcohols **4a**, **4e** and **4f**, which were derivatised with GITC. The relevant signals are marked accordingly. In each case, samples were taken from a reaction solution containing the enzyme, and standards of the respective compounds were used. These are presented and compared separately below for the sake of clarity.

Each sample (20 µl) taken from an enzyme solution was dissolved in 180 µl of acetonitrile (LC/MS grade) and mixed with 200 µl of a GITC solution (1.5 mM, dissolved in acetonitrile, containing 1.5 mM of triethylamine). This solution was shaken at room temperature for 30 minutes at 1000 rpm, then centrifuged to separate any solids, and transferred to a sample vial. To measure the standards, 10 mM stock solutions were first prepared in acetonitrile. Following the same procedure used for sample preparation, 20 µl of these solutions were added to 180 µl of acetonitrile and mixed with 200 µl of the GITC solution. After incubation for 30 minutes at room temperature and 1000 rpm, these solutions were also centrifuged and transferred to vials.

Determination of enantiomeric excess for **4a**

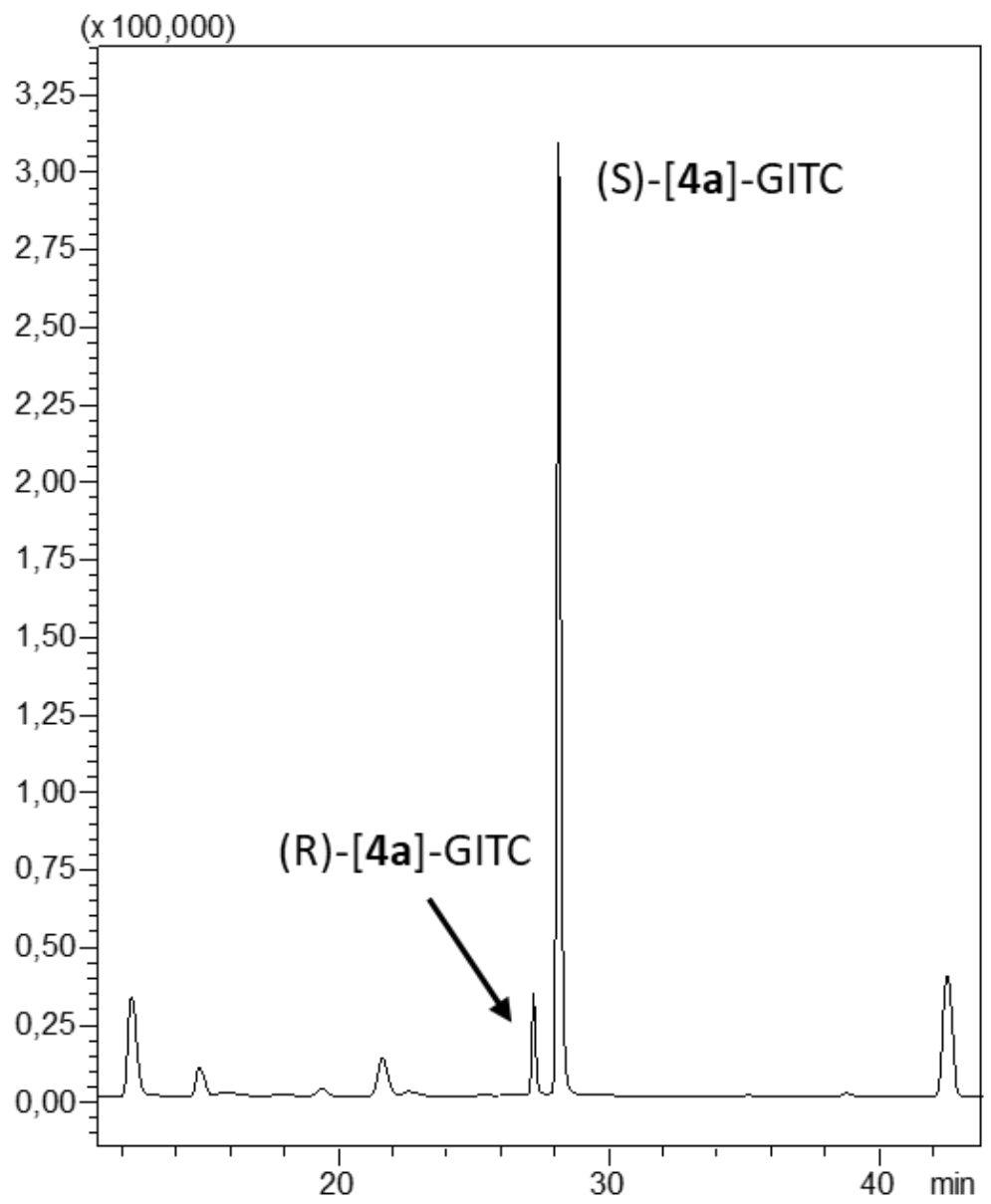

Figure S49. Chromatogram of a sample from the enzymatic reaction for the production of **4a**, after the derivatisation with GITC. Measurement in Scan mode.

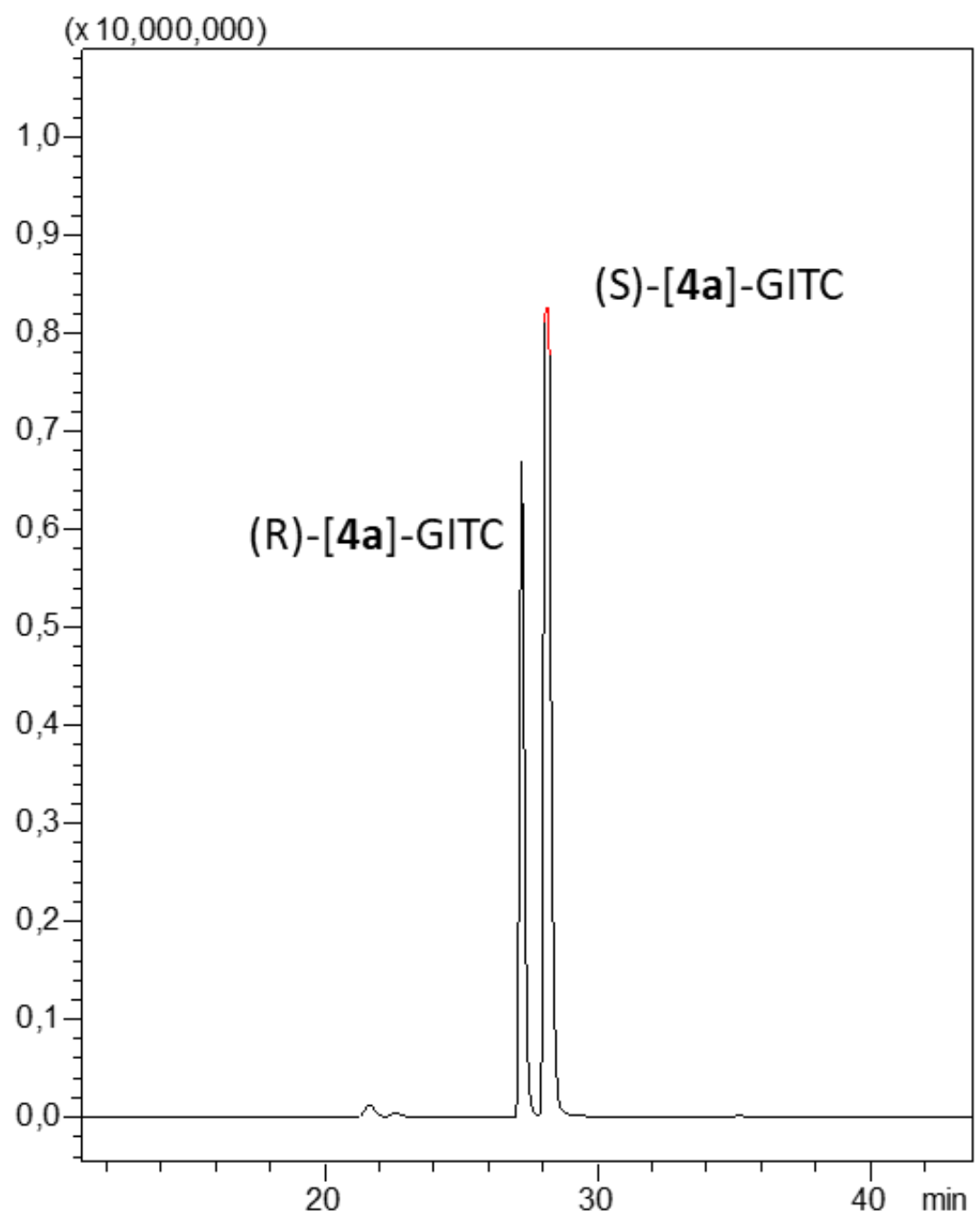

Figure S50. Chromatogram of the standard measurement of [**4a**]-GITC. The racemate used with a small addition of the (*S*)-enantiomer in order to tell the peaks apart. Measurement in SIM Mode.

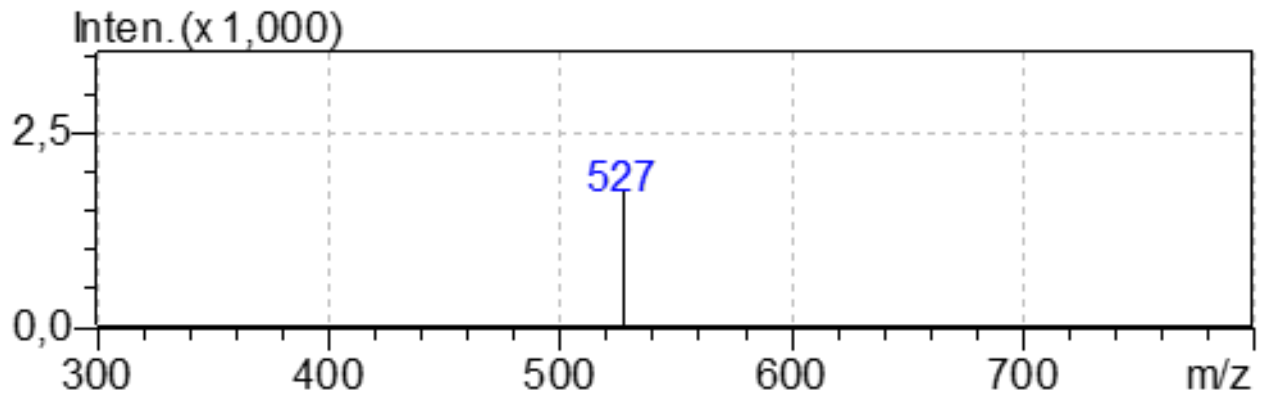

Figure S7. Mass spectrum of [**4a**]-GITC+H.

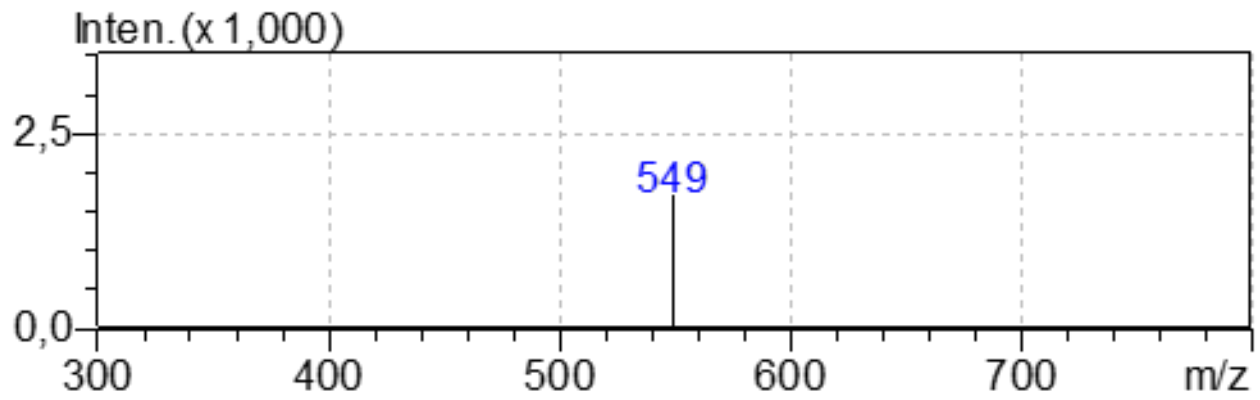

Figure S51. Mass spectrum of the Na-adduct of [**4a**]-GITC.

The enantiomeric excess (ee), calculated as the ratio of the difference between the peak areas to the sum of these areas, was 82% for the (*S*)-enantiomer in this reaction. The expected peaks in the mass spectrum for both [**4a**]-GITC+H and its sodium adduct confirm the identity of the compound. In addition, the retention times of the standards match those of the sample.

<u>Determination of the enantiomeric excess for **4e**</u>

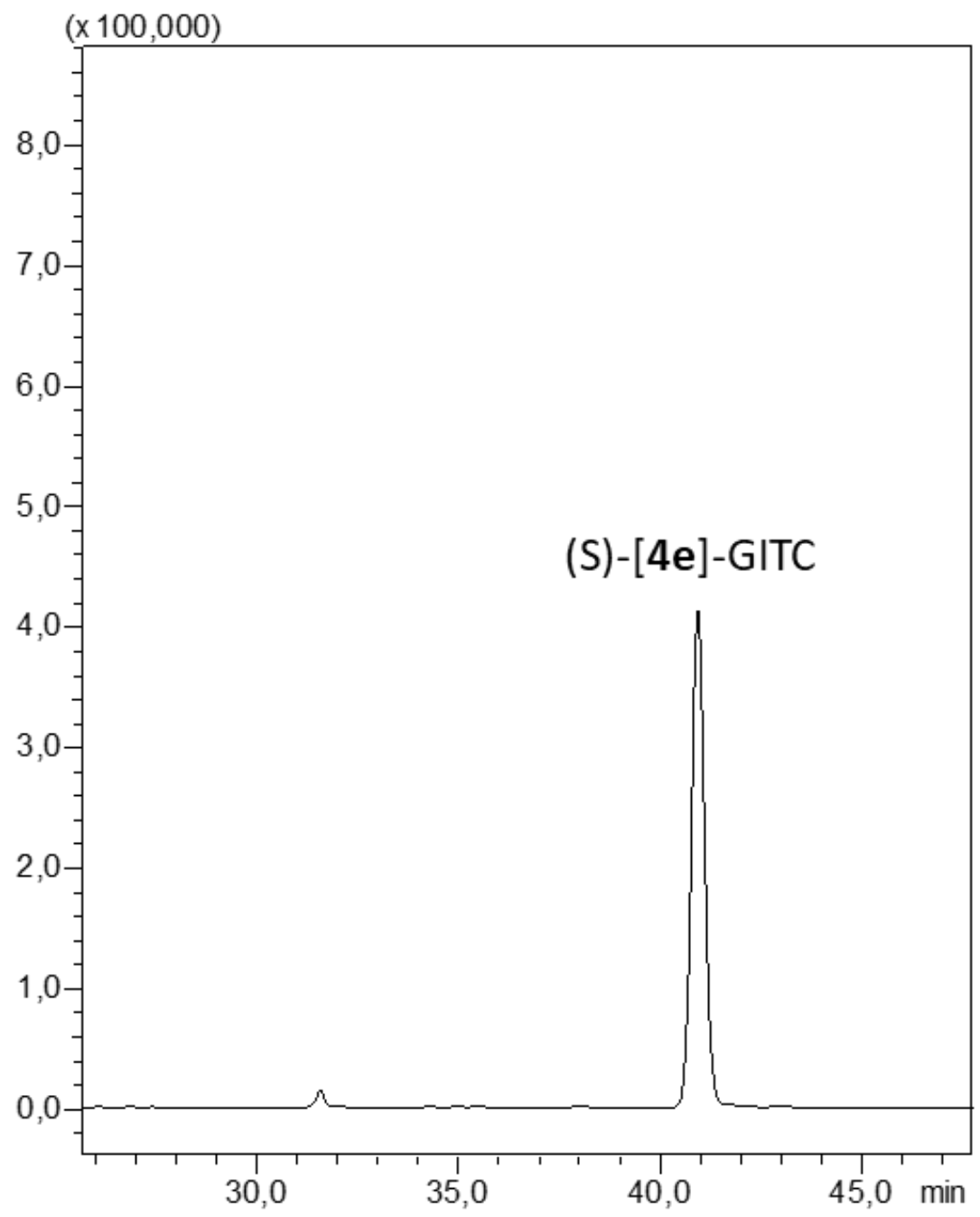

Figure S52. Chromatogram of a sample from the enzymatic reaction for the production of **4e**, after the derivatisation with GITC. Measurement in Scan Mode.

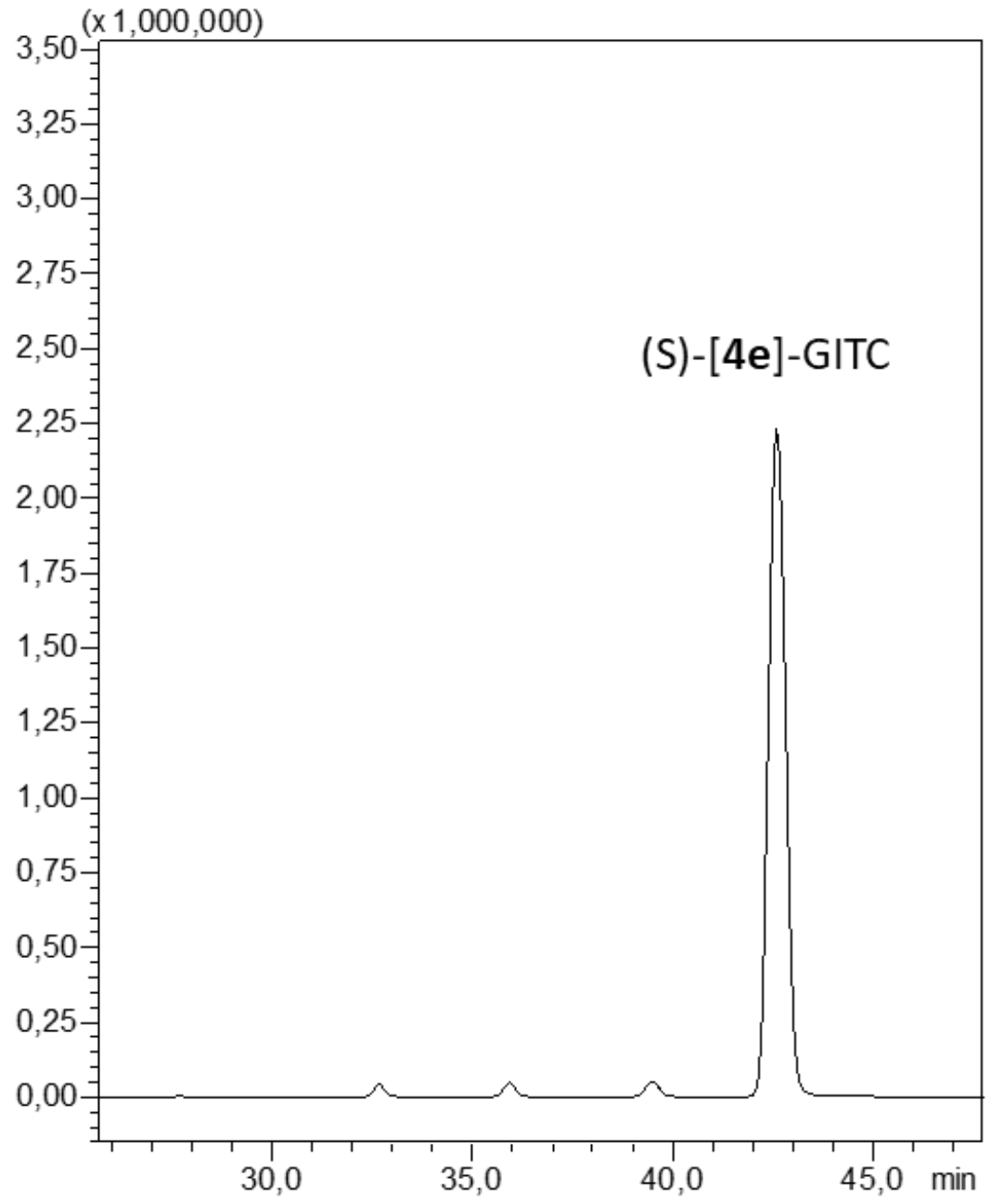

Figure S53. Chromatogram of the standard measurement of the (*S*)-enantiomer of [**4e**]-GITC. Measurement in Scan Mode.

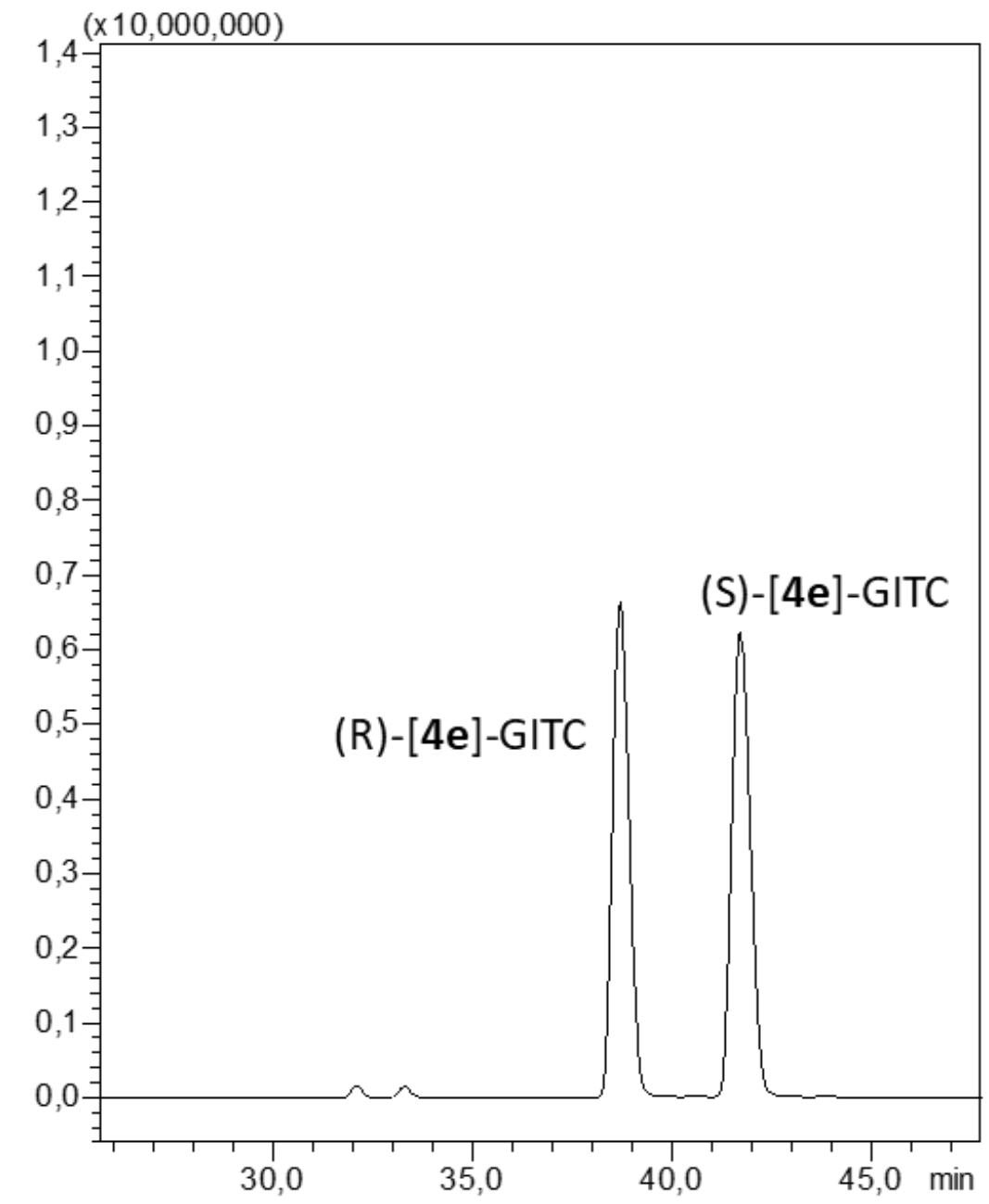

Figure S54. Chromatogram of the standard measurement of [**4e**]-GITC as a racemate. Measurement in SIM Mode.

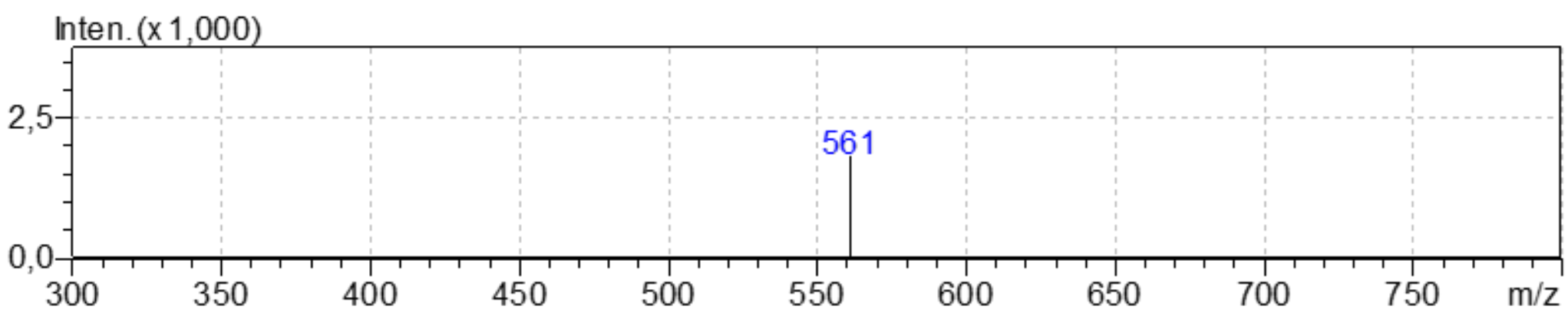

Figure S55. Mass spectrum of [**4e**]-GITC+H.

For **4e**, an enantiomeric excess of > 99.9% was observed for the (*S*)-enantiomer. The (R)-enantiomer could not even be detected. It should be noted that the retention times of the standards differ from those of the sample. This can be explained by differences in the matrix, which arise because the standards were not extracted from an aqueous solution containing the enzyme and residues or by-products from enzyme production. These additional components accumulate during the concentration of the enzyme solution and can influence the elution behavior of individual analytes, resulting in the deviations in retention times. The deviation amounts to approximately one minute; nevertheless, it can be concluded that the signal obtained for the sample corresponds to the (*S*)-enantiomer. Nonetheless, the identity of the analyte as 4e was unequivocally confirmed by mass spectrometry. Additional low-intensity signals appear in the chromatograms of the standards (Figures S10 and S12); these are due to the purity of the standards, which was only 96%. Unfortunately, however, it was not possible to obtain standards of higher purity. Nonetheless, this does not pose a problem for the identification of the enantiomers.

Determination of the enantiomeric excess for **4f**

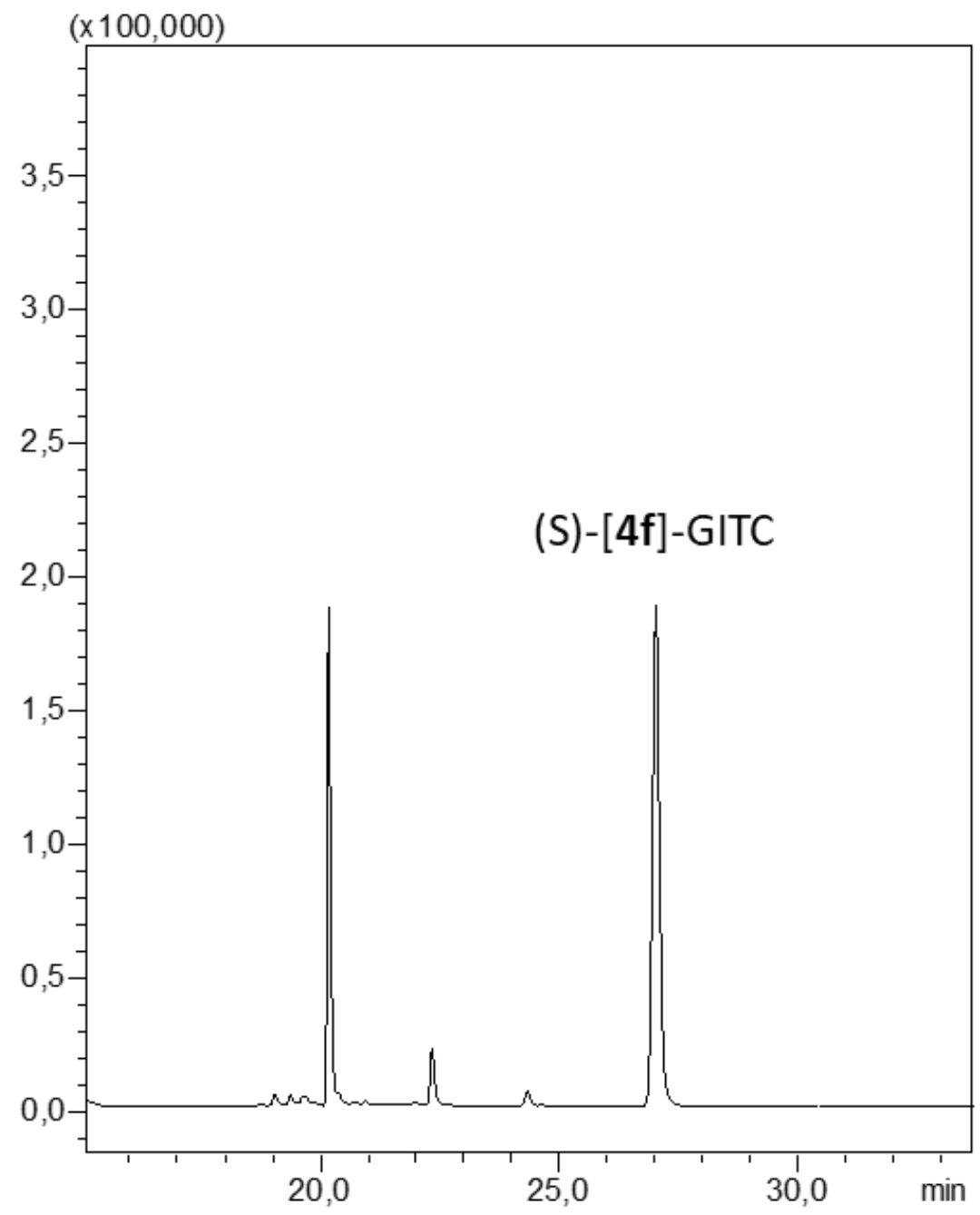

Figure S56. Chromatogram of a sample from the enzymatic reaction for the production of **4f**, after the derivatisation with GITC. Measurement in Scan Mode.

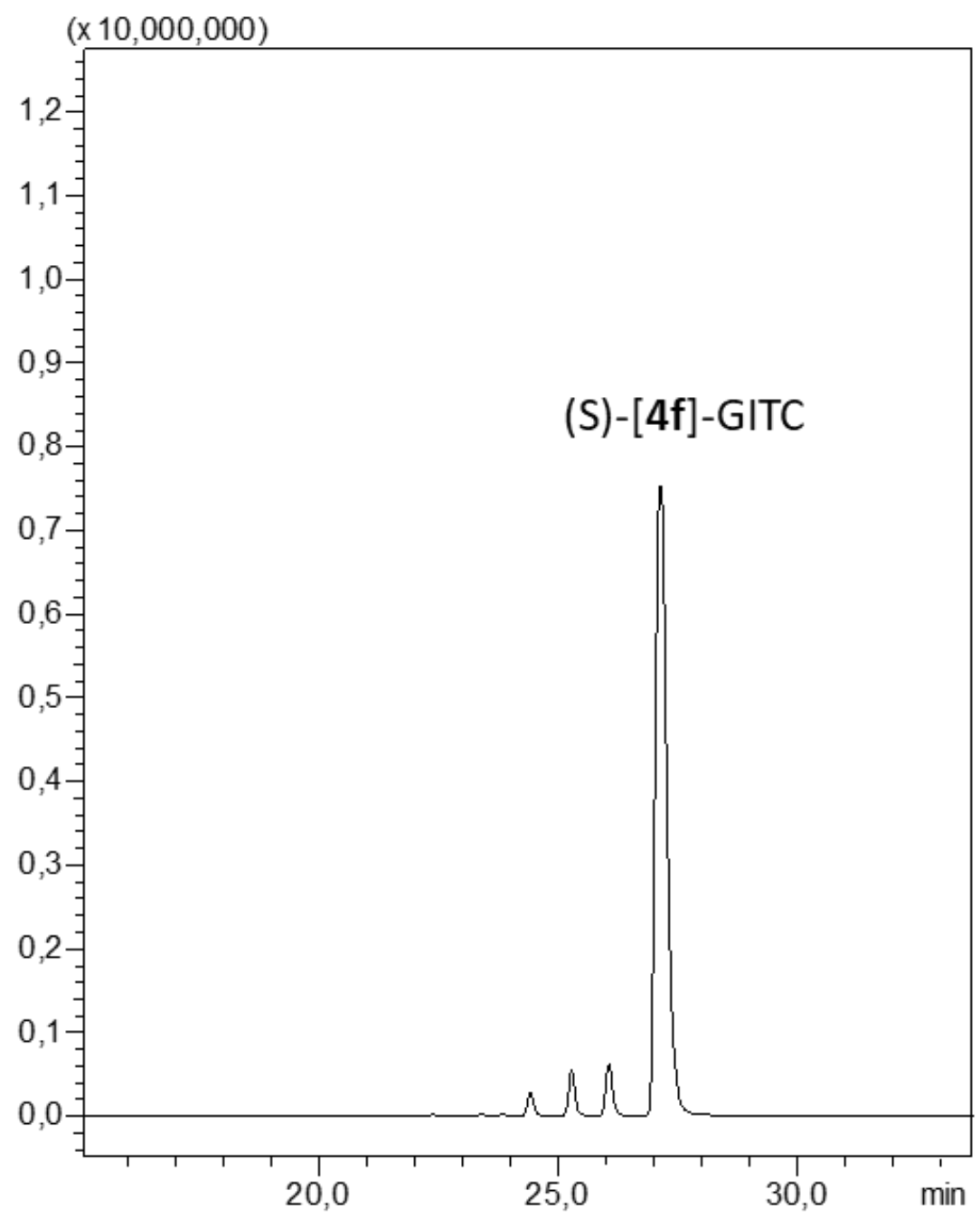

Figure S57. Chromatogram of the standard measurement of the (*S*)-enantiomer of [**4f**]-GITC. Measurement in Scan Mode.

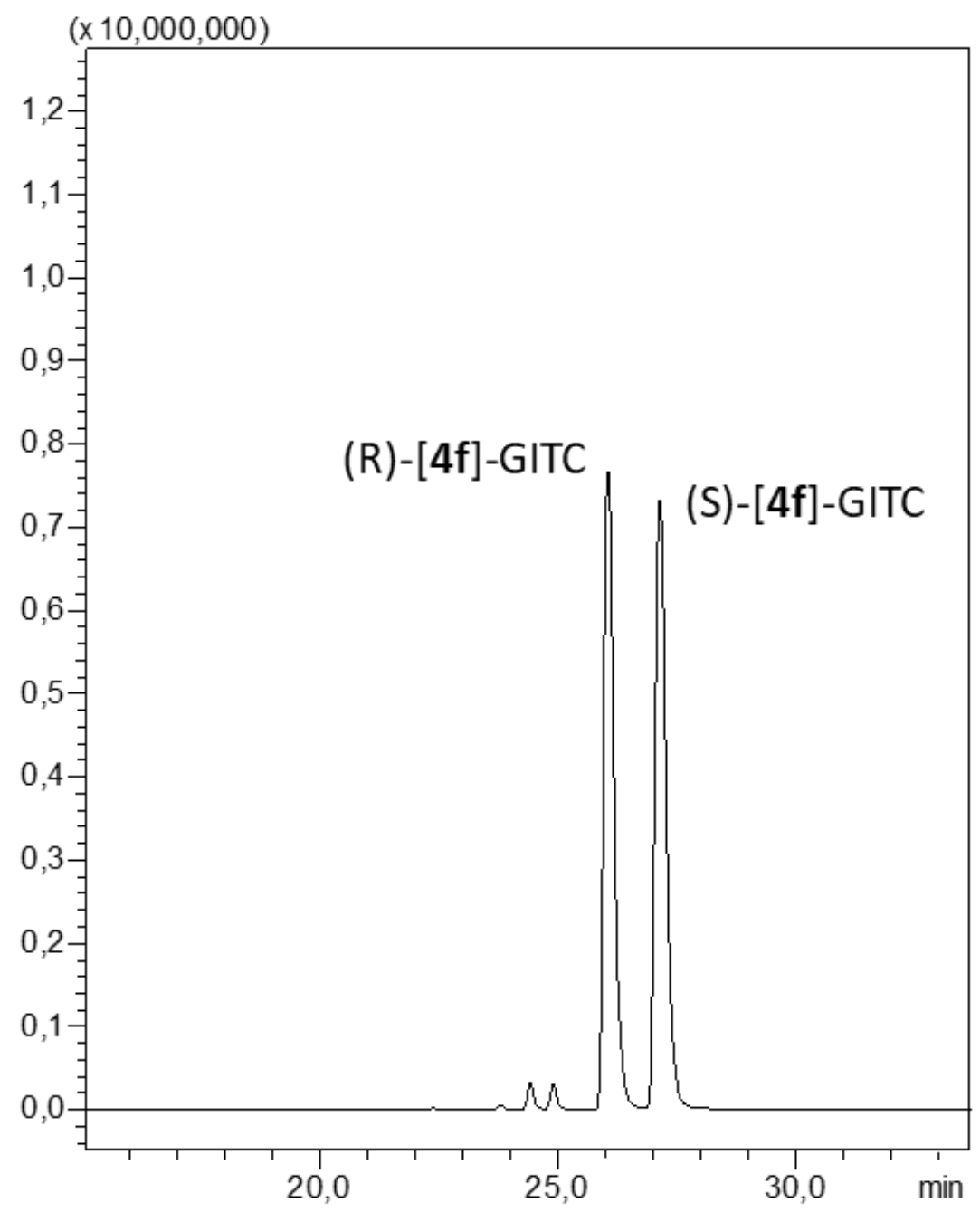

Figure S58. Chromatogram of the standard measurement of [**4e**]-GITC as a racemate. Measurement in Scan Mode.

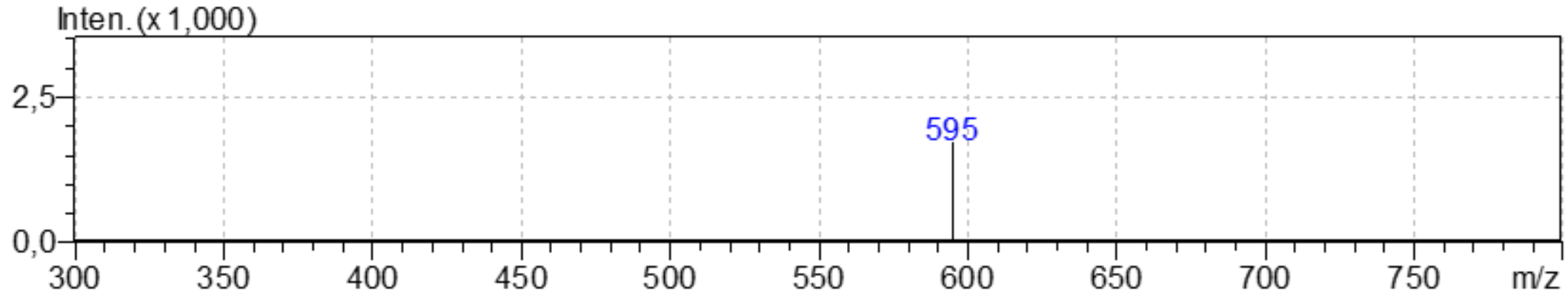

Figure S59. Mass spectrum of [**4f**]-GITC+H.

The enantiomeric excess for the (*S*)-enantiomer of **4f** was calculated to be 99.57%. It is noticeable that additional signals appear in the chromatograms for the standards of **4f**, which can be attributed to a purity of only 95%. Unfortunately, it was not possible to obtain standards of higher purity. Nevertheless, the signals can be identified beyond doubt, and the attached mass spectrum supports this conclusion. The additional peak in Figure S13 with a retention time is, in high probability, an isomer of the same mass, which is why it is also detected in SIM mode. It is likely to be an isomer in which the aromatic ring has been chlorinated at a different position and which could not be completely separated during the manufacturer's purification process. Due to the high sensitivity of the MS, the peak appears to be just as large as the signal from the compound of interest, [**4f**]-GITC.
